# Supplementary figures and images for: High-throughput mapping of the phage resistance landscape in E. coli
Source: PLoS Biol. 2020 Oct 13;18(10):e3000877. doi: 10.1371/journal.pbio.3000877 (PMC7553319; doi:10.1371/journal.pbio.3000877)

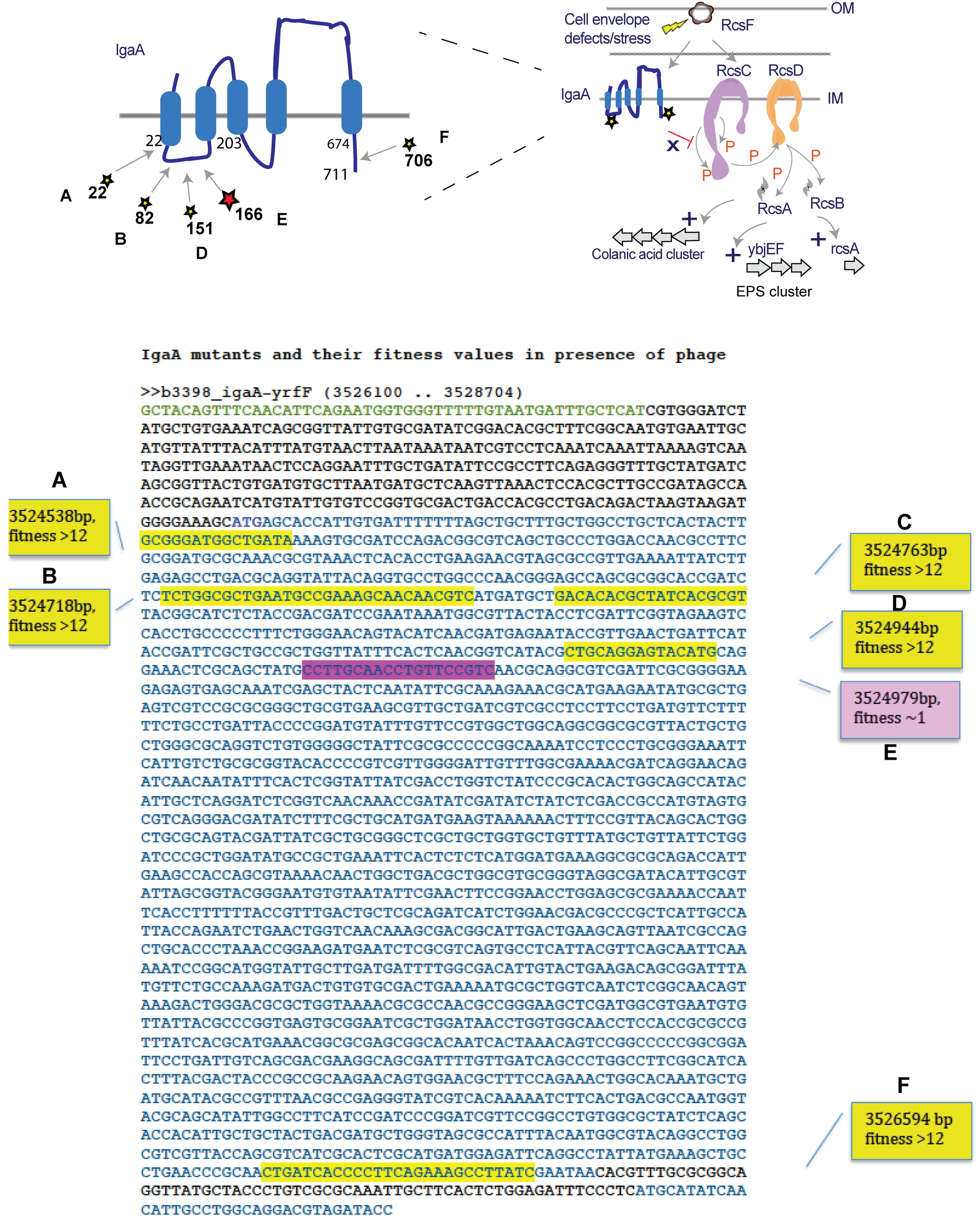

Supplement: S1 Fig — igaA mutants and their overall fitness scores from E. coli K-12 RB-TnSeq screen (S1 Table). Schematic of Rcs phosphorelay with the predicted topology of IgaA [165] is shown at the top. RB-TnSeq mutants in IgaA are mapped to the predicted topology of IgaA (top) and also mapped on to igaA nucleotide sequence, with mutant position and fitness scores. Mutants A, B, and D were constructed and their mucoidy and phage resistance phenotype were confirmed (mutant A data are shown in Fig 5A). Though full-length deletion of igaA has not been possible, our results indicate that disruption between amino acid 22 and 151 is dispensable. Strain with igaA mutant A was further subjected to EOP and RNA-seq analysis presented in the main text. The underlying data for this figure can be found in S1 Data. EOP, efficiency of plating; RB-TnSeq, random barcode transposon site sequencing; RNA-seq, RNA sequencing. (TIF) [file pbio.3000877.s001.tif]

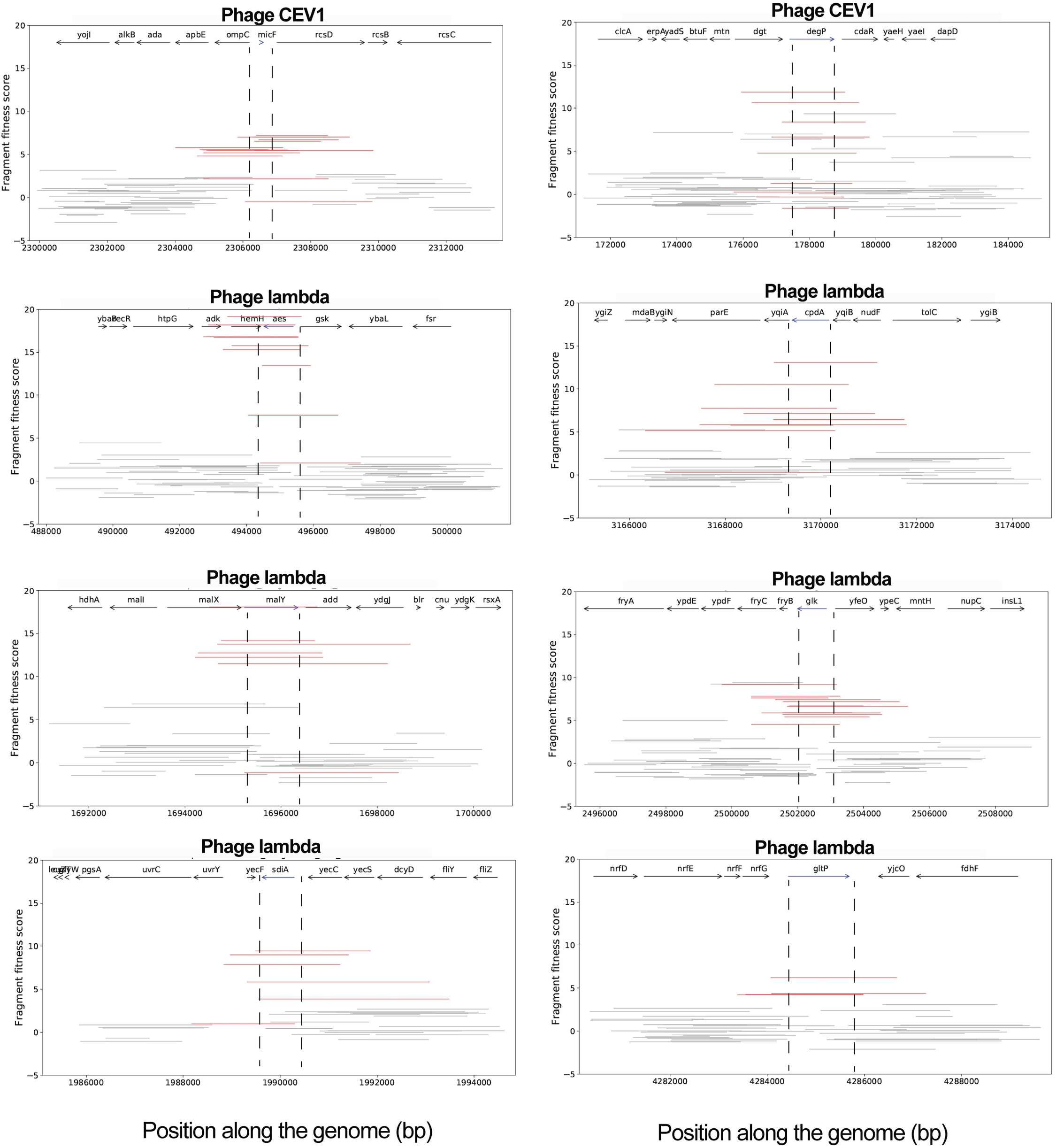

Supplement: S2 Fig — Following top candidates are shown: high-scoring fragments encoding micF and degP for CEV1 phage; aes, cpdA, malY, glk, sdiA, and gltP for λ phage cI857. Red lines represent fragments covering highlighted genes completely (start to stop codon), and gray-colored fragments either cover the highlighted gene partially or do not cover the highlighted gene completely. The underlying data for this figure can be found in S1 Data. Dub-seq, dual-barcoded shotgun expression library sequencing. (TIF) [file pbio.3000877.s002.tif]

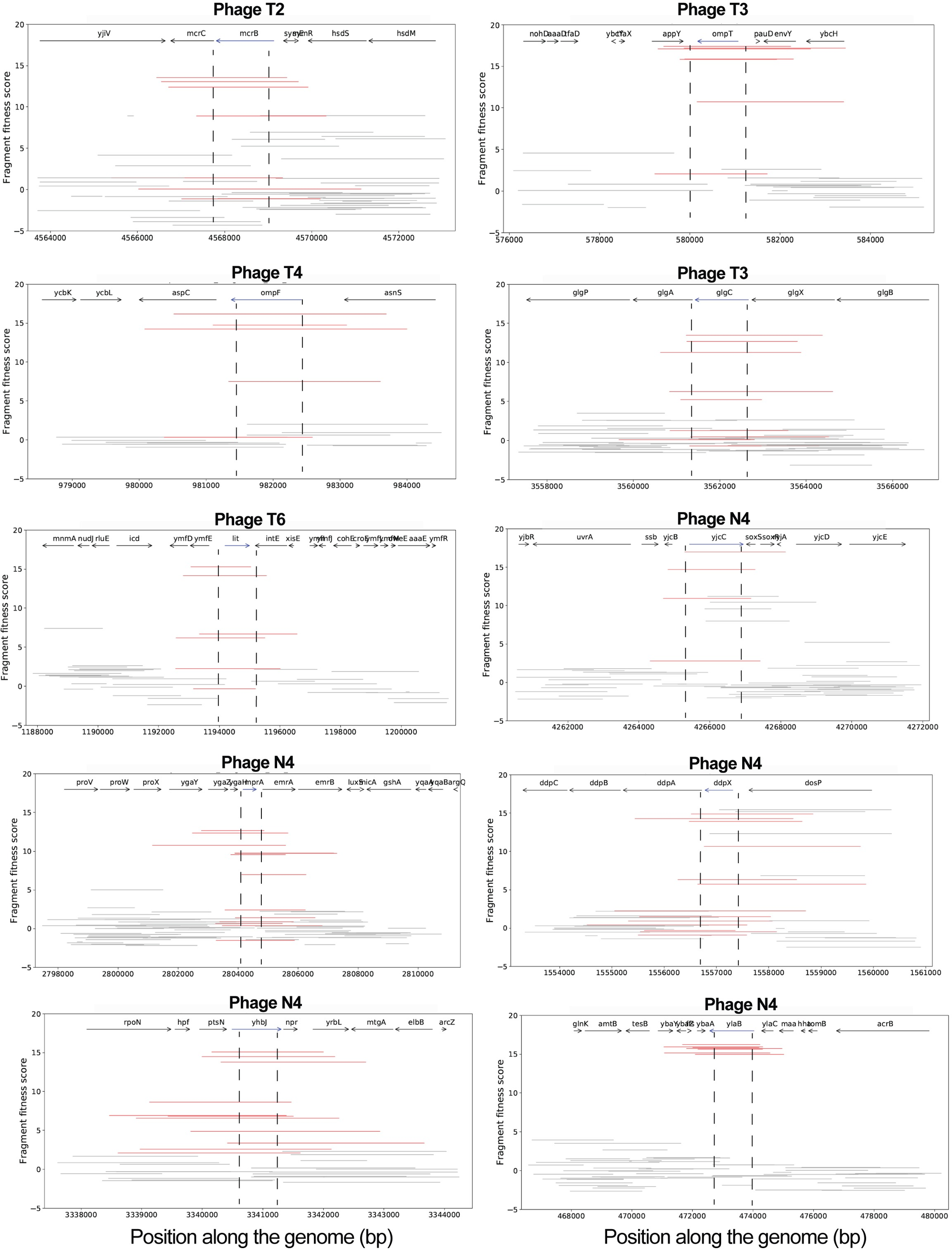

Supplement: S3 Fig — Following top candidates are shown: high-scoring fragments encoding mcrB for T2 phage; ompT and glgC for T3 phage; ompF for T4 phage; lit for T6 phage; yjcC (pdeC), mprA, ddpX, yhbJ (rapZ), ylaB (pdeB) for N4 phage. Red lines represent fragments covering highlighted genes completely (start to stop codon), whereas gray-colored fragments either cover the highlighted gene partially or do not cover the highlighted gene completely. The underlying data for this figure can be found in S1 Data. Dub-seq, dual-barcoded shotgun expression library sequencing. (TIF) [file pbio.3000877.s003.tif]

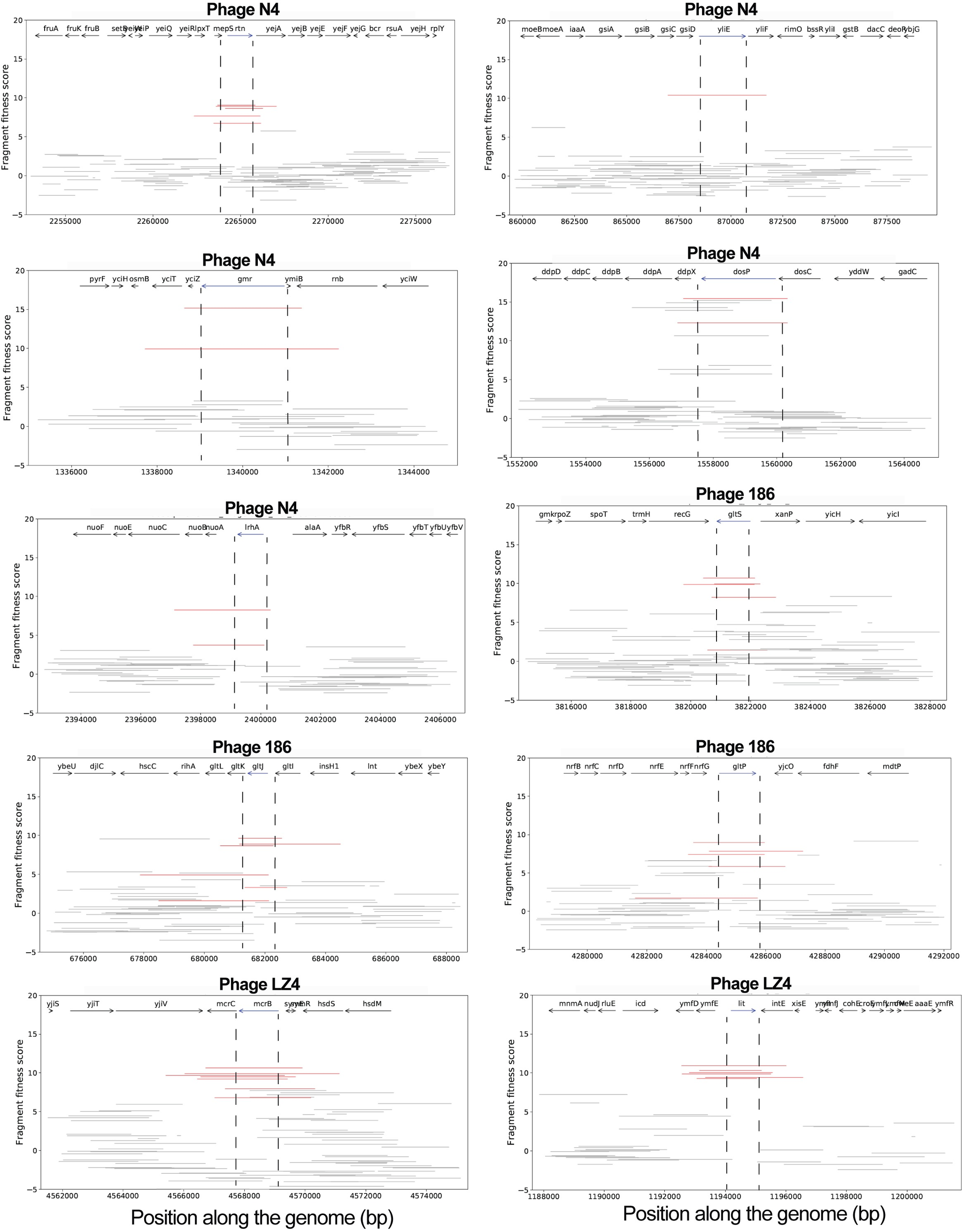

Supplement: S4 Fig — Following top candidates are shown: high-scoring fragments encoding rtn(pdeN), yliE (pdeI), gmr (pdeR), dosP (pdeO), and lrhA for N4 phage; gltS, gltJ, and gltP for 186 phage; mcrB and lit for LZ4 phage. Red lines represent fragments covering highlighted genes completely (start to stop codon), whereas gray-colored fragments either cover the highlighted gene partially or do not cover the highlighted gene completely. The underlying data for this figure can be found in S1 Data. Dub-seq, dual-barcoded shotgun expression library sequencing. (TIF) [file pbio.3000877.s004.tif]

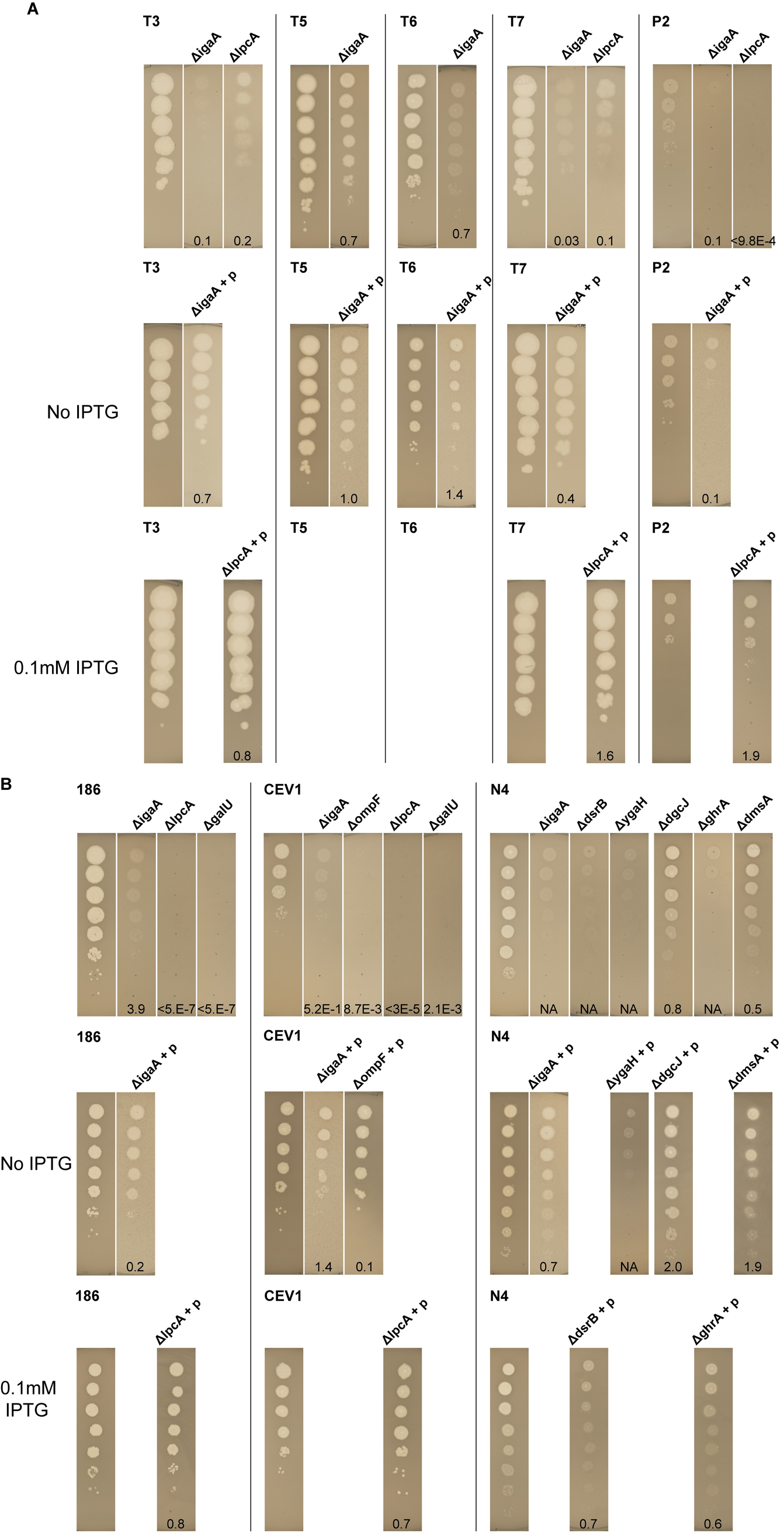

Supplement: S5 Fig — (A and B) EOP experiments with Keio strains and ASKA plasmid complementation of respective genes (indicated by +p) in the presence of different phages. We used no IPTG or 0.1 mM IPTG for inducing expression of genes from ASKA plasmid. All experiments were in E. coli K-12 BW25113 strain. EOP, efficiency of plating. (TIF) [file pbio.3000877.s005.tif]

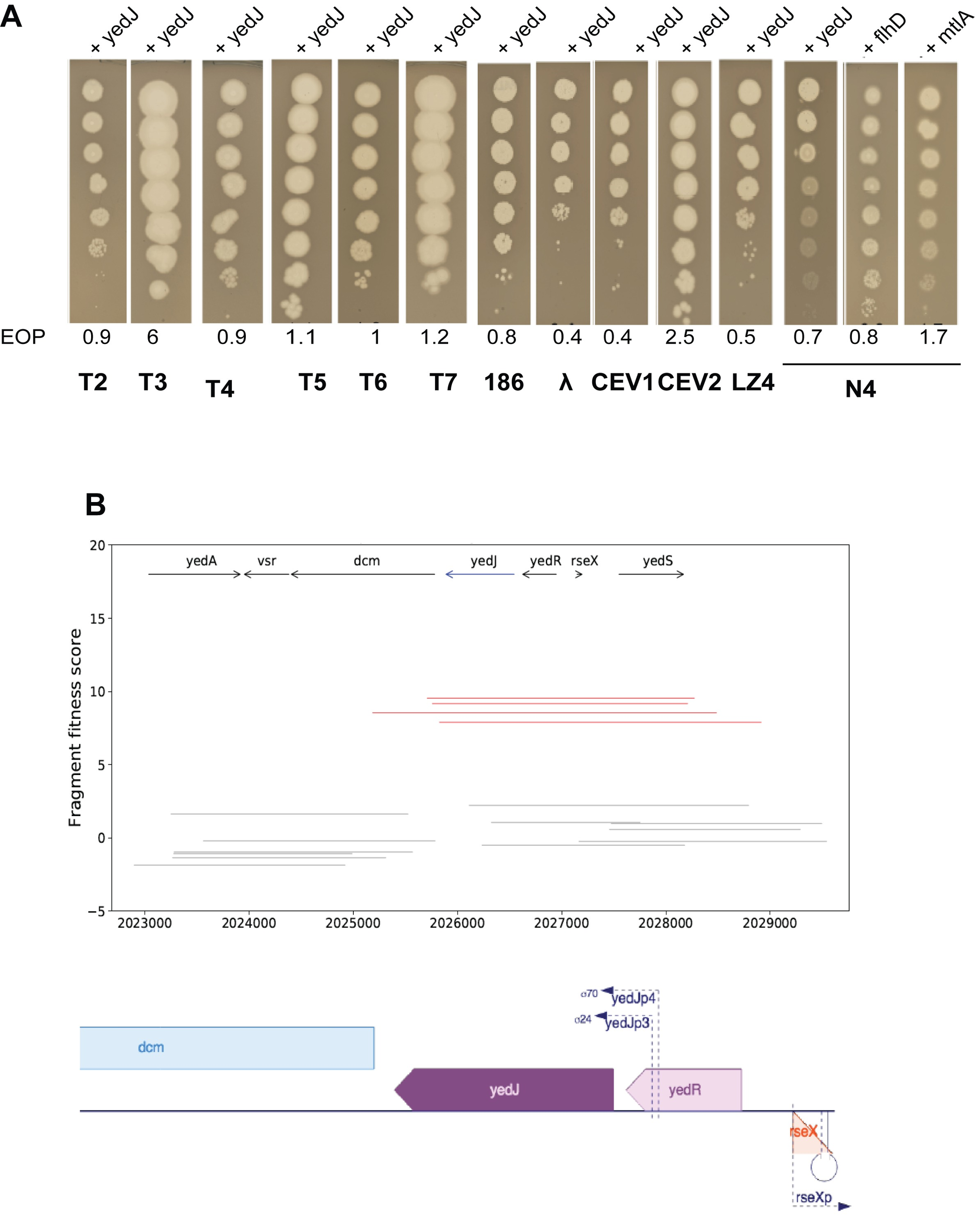

Supplement: S6 Fig — (A) EOP experiments with ASKA plasmid expressing genes (shown as +gene names) in the presence of different phages. We used no IPTG and 0.1 mM IPTG for inducing expression of genes from ASKA plasmid. We used E. coli K-12 BW25113 strain with an empty vector for EOP calculations. (B) Dub-seq viewer plots for high-scoring fragments (red bars) encoding yedJ, along with neighboring genes (gray bars) with fitness score in the presence of T4 phage on the y-axis. The Ecocyc operon [171] view for yedJ regions is on the bottom. The genomic fragments encoding yedJ also encode a small RNA rseX (RNA suppressor of extracytoplasmic stress protease) that binds to RNA binding protein Hfq (a global regulator) and specifically targets ompA and ompC mRNAs. Our library does not have genomic fragments that can resolve rseX contribution to fitness independently. Overexpression of RseX has also been shown to increase biofilm formation [260], indicating the role of yedJ-rseX locus on resistance to T4 phage and other phages. The underlying data for this figure can be found in S1 Data. Dub-seq, dual-barcoded shotgun expression library sequencing; EOP, efficiency of plating. (TIF) [file pbio.3000877.s006.tif]

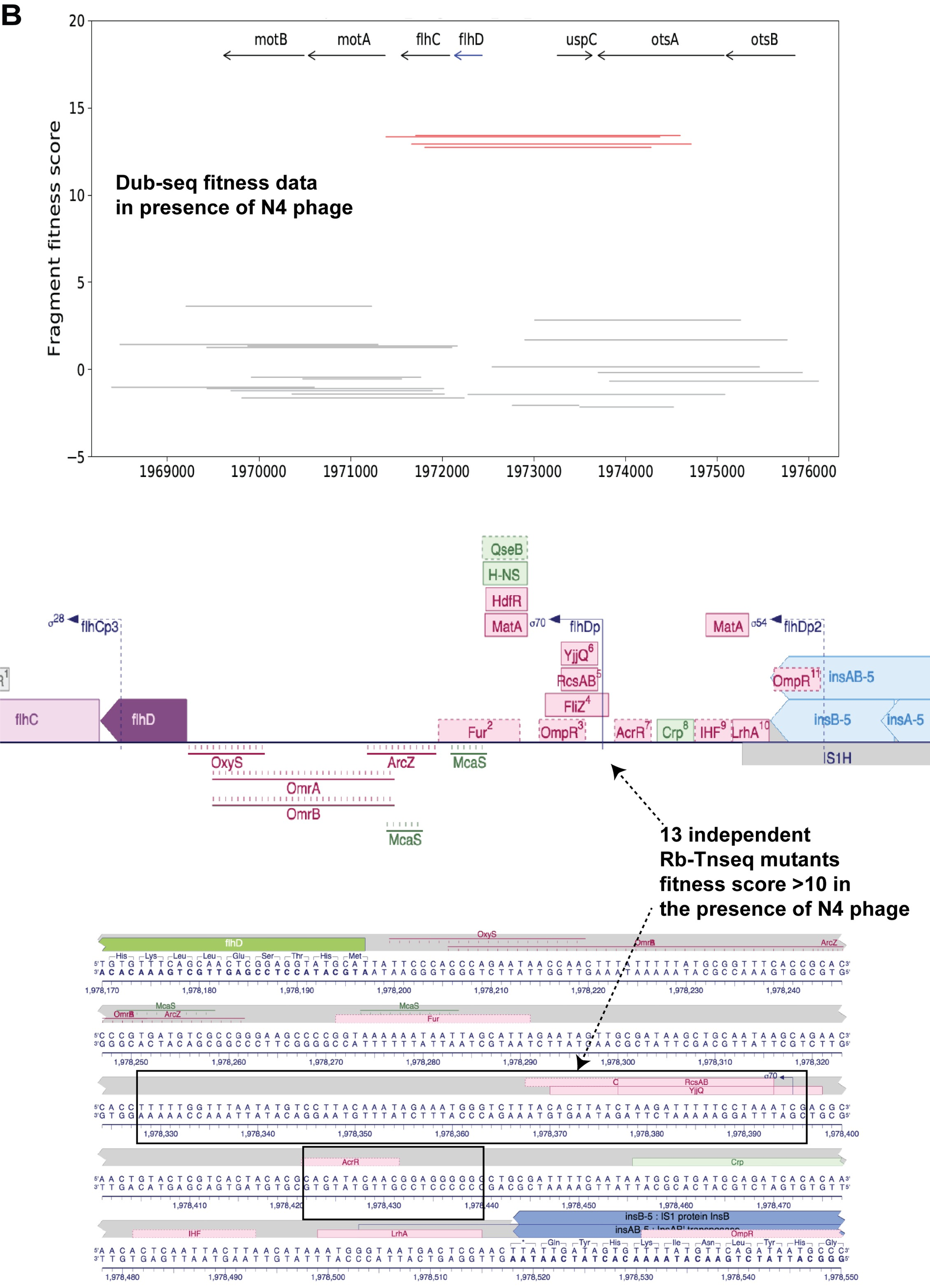

Supplement: S7 Fig — (A) Dub-seq viewer plots for high-scoring fragments (red bars) encoding flhD and upstream region along with neighboring genes (gray bars) with fitness score on the y-axis. Overexpression of flhD failed to demonstrate strong phage plating defects in our EOP validation experiments (S6A Fig). (B) The Ecocyc operon view of the regulatory region upstream of flhD with multiple transcription factor binding sites [171]. (C) Zoom-in view of the upstream region of flhD operon with arrows identifying the location of 13 RB-TnSeq insertion mutants that have fitness score >10 in the presence of N4 phage. These results indicate the role of flhD regulatory region on N4 phage growth. We speculate that these TnSeq mutants of flhD regulatory region may not be overexpressing flhD, as our EOP experiments failed to validate flhD overexpression as the cause of N4 phage resistance. The underlying data for this figure can be found in S1 Data. Dub-seq, dual-barcoded shotgun expression library sequencing; RB-TnSeq, random barcode transposon site sequencing. (TIF) [file pbio.3000877.s007.tif]

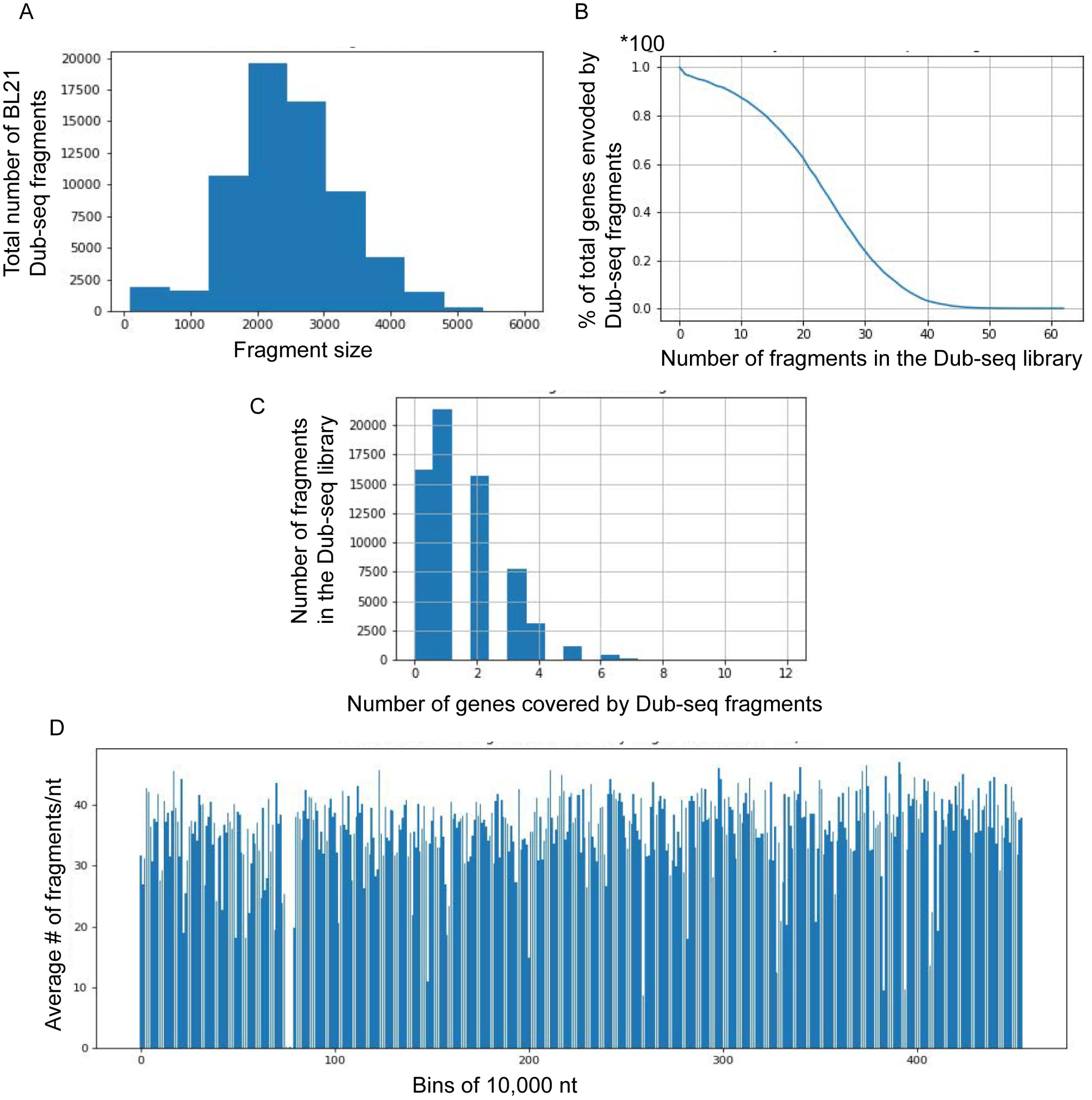

Supplement: S8 Fig — (A) The fragment insert size distribution in the E. coli BL21 Dub-seq library. (B) Cumulative distribution plot showing the percentage of genes in the E. coli BL21 genome (y-axis) covered by a number of independent genomic fragments (x-axis). (C) The distribution of the number of genes that are completely covered (start to stop codon) per genomic fragment in the E. coli BL21 Dub-seq library. (D) Genome coverage of E. coli BL21 Dub-seq library in 10,000-kB windows mapped to E. coli BL21-DE3. The underlying data for this figure can be found in S1 Data. Dub-seq, dual-barcoded shotgun expression library sequencing. (TIF) [file pbio.3000877.s008.tif]
