# Supplementary material for: High-throughput mapping of the phage resistance landscape in E. coli
Source: PLoS Biol. 2020 Oct 13;18(10):e3000877. doi: 10.1371/journal.pbio.3000877 (PMC7553319; doi:10.1371/journal.pbio.3000877)
Supplement: S2 Table — MOI, multiplicity of infection. (PDF) [file pbio.3000877.s010.pdf]

S2 Table

Summary of known and new high fitness score hits per phage per screen at any MOI.

E coli K-12 BW25113 RB-TnSeq screen: Total hits 354		
Phages	Unique host factors	Known
T2	9	1
T3	12	4
T4	11	4
T5	1	1
T6	1	1
T7	10	10
N4	8	3
c1857 Lambda	16	16
P1	11	1
P2	15	not known
186	21	13
CEV1	13	not known
CEV2	1	1
LZ4	7	not known
**E coli K-12 MG1655 CRISPRi screen: Total hits 542**		
Phages	Unique host factors	Known
T2	4	1
T3	139	2
T4	24	2
T5	9	1
T6	7	1
T7	ND	--
N4	46	2
c1857 Lambda	15	8
P1	ND	--
P2	ND	--
186	26	11
CEV1	40	not known

CEV2	17	1
LZ4	7	not known
**E coli K-12 BW25113 Dub-seq screen: Total hits 233**		
Phages	Unique host factors	Known
T2	7	not known
T3	8	not known
T4	6	not known
T5	4	not known
T6	4	not known
T7	5	1
N4	20	1
c1857 Lambda	21	not known
P1	3	--
P2	ND	--
186	21	not known
CEV1	12	not known
CEV2	3	not known
LZ4	12	not known
**E coli BL21 RB-TnSeq screen: Total hits 115**		
Phages	Unique host factors	Known
T2	5	1
T3	5	2
T4	3	3
T5	1	1
T6	1	1
T7	5	2
N4	NA	--
c1857 Lambda	18	--
P1	2	--
P2	ND	--
186	NA	--
CEV1	9	not known

CEV2	1	not known
LZ4	2	not known
**E coli BL21 Dub-seq screen: Total hits 39**		
Phages	Unique host factors	Known
T2	2	not known
T3	ND	not known
T4	1	not known
T5	2	not known
T6	2	not known
T7	2	not known
N4	NA	not known
c1857 Lambda	27	not known
P1	3	not known
P2	1	not known
186	NA	not known
CEV1	2	not known
CEV2	3	not known
LZ4	1	not known
