# Supplementary material for: High-throughput mapping of the phage resistance landscape in E. coli
Source: PLoS Biol. 2020 Oct 13;18(10):e3000877. doi: 10.1371/journal.pbio.3000877 (PMC7553319; doi:10.1371/journal.pbio.3000877)
Supplement: S3 Table — CRISPRi, CRISPR interference; Dub-seq, dual-barcoded shotgun expression library sequencing; RB-TnSeq, random barcode transposon site sequencing. (PDF) [file pbio.3000877.s011.pdf]

S3 Table: Mapping RB-TnSeq, CRISPRi and Dub-seq screen hits to the reported phage resistance data from literature. Each gene in this table is associated with an earlier reported data, and whether we see that gene uncovered in our genome-wide screen platform. Instances with 'Yes', indicate that our screens uncover that gene, 'no' means, our screens do not uncover that genes and 'NA' means the data is not applicable for that particular assay.

Phages	Bacterial Strain	host factor	function	References	RB-TnSeq	CRISPRi	Dub-seq
T2	E coli K-12	fadL	long-chain fatty acid outer membrane transporter	[1–3]	Yes	Yes	NA
T2	E coli B	ompF	outer membrane porin 1a	[1–4]	yes in BL21	NA	NA
T2	E coli B	LPS		[1–4]	yes, in BL21	NA	NA
T3	E coli B Berkeley	waaC	LPS biosynthesis enzyme ADP-heptose:LPS heptosyltransferase 1	[5]	yes, in BL21 & K-12	yes, in K-12	NA
T3	E coli B Berkeley	waaG	LPS biosynthesis enzyme lipopolysaccharide glucosyltransferase I	[5]	yes, in BL21 & K-12	No	NA
T3	E coli B Berkeley	waaF	LPS biosynthesis enzyme	[5]	yes, in BL21 & K-12	yes, in K-12	NA
T3	E coli B Berkeley	galU	UTP—glucose-1-phosphate uridylyltransferase	[6]	yes, in BL21 & K-12	no	NA
T4	E coli K-12 MG1655/B W25113	ompC	outer membrane porin protein C	[1–3]; [6–9]	Yes	Yes	NA
T4	E coli K-12 MG1655	ompC with LPS		[1–3]; [6–9]	Yes	NA	NA
T4	E coli K-12 MG1655	ompR	osmolarity response regulator	[10]	Yes	No	NA
T4	E coli K-12 MG1655	yrfF	Inner membrane protein IgaA	[10]	Yes	yes	NA
T4	E coli K-12 MG1655	rfaD	LPS biosynthesis enzyme	[10]	yes	no	NA

T4	E coli K-12 MG1655	yfjU	CP4-57 prophage; conserved protein	[10]	no	no	NA
T4	E coli K-12 MG1655	rseA	anti-sigma factor	[10]	no	no	NA
T4	E coli K-12 MG1655	hipB	DNA-binding transcriptional regulator	[10]	no	no	NA
T4	E coli K-12 MG1655	lit	e14 prophage; cell death peptidase Lit	[11]	NA	NA	No (yes for T6, CEV1 & LZ4)
T4	E coli B Berkeley	waaC	LPS biosynthesis enzyme	[5] [9]	yes, in BL21	NA	NA
T4	E coli B Berkeley	waaG	LPS biosynthesis enzyme	[5] [9]	yes, in BL21	NA	NA
T4	E coli B Berkeley	waaF	LPS biosynthesis enzyme	[5] [9]	yes, in BL21	NA	NA
T4	E coli B	galU	glucose-1-phosphate uridylyltransferase	[9,12][6]	Yes , in K-12	NA	NA
T4	E coli B	LPS		[1–3]; [6–9][13][14]	Yes	NA	NA
T5	E coli K-12 MG1655	fhuA	ferrichrome outer membrane transporter	[1–3]; [6–9][14]	Yes	Yes	NA
T5	E coli B	fhuA	ferrichrome outer membrane transporter	[1–3]; [6–9][14]	Yes	NA	NA
T6	E coli K-12 MG1655	tsx	nucleoside channel, receptor of phage T6	[1–3]; [6–9][15] [16,17]	Yes	Yes	NA
T6	E coli B	tsx		[1–3]; [6–9][15] [16,17]	Yes	NA	NA
T7	E coli K-12 BW25113	trxA	Thioredoxin A	[16–18]	Yes	no	NA
T7	E coli K-12 BW25113	LPS		[6–9][14]	Yes	NA	NA
T7	E coli K-12 BW25113	cmk	CML/dCAMP kinase	[19]	no	no	NA
T7	E coli K-12 BW25113	gmhA/lpcA	LPS biosynthesis enzyme	[19]	Yes	NA	NA

T7	E coli K-12 BW25113	gmhB	LPS biosynthesis enzyme	[19]	Yes	NA	NA
T7	E coli K-12 BW25113	waaC	LPS biosynthesis enzyme	[19]	Yes	NA	NA
T7	E coli K-12 BW25113	gmhD/rfaD/waaD	LPS biosynthesis enzyme	[19]	Yes	NA	NA
T7	E coli K-12 BW25113	gmhE/waaE	LPS biosynthesis enzyme	[19]	Yes	NA	NA
T7	E coli K-12 BW25113	waaF	LPS biosynthesis enzyme	[19]	Yes	NA	NA
T7	E coli K-12 BW25113	waaG	LPS biosynthesis enzyme	[19]	Yes	NA	NA
T7	E coli K-12 BW25113	galU	LPS biosynthesis enzyme	[19]	Yes	NA	NA
T7	E coli K-12 BW25113	waaR	LPS biosynthesis enzyme	[19]	Yes	NA	NA
T7	E coli K-12 BW25113	rcsA	DNA-binding transcriptional activator RcsA	[19]	NA	NA	Yes
T7	E coli B	LPS		[6–9][14]	Yes	NA	NA
N4	E coli K-12 BW25113	nfrA	bacteriophage N4 receptor, outer membrane subunit	[20]	Yes	yes	NA
N4	E coli K-12 BW25113	nfrB	bacteriophage N4 receptor,Inner membrane subunit	[20]	Yes	Yes	NA
N4	E coli K-12 BW25113	nfrC	UDP-N-acetylglucosamine 2-epimerase	[20]	Yes	no	NA
N4	E coli K-12	rtn	Resistant to N4	[21]	NA	NA	Yes
lambda	E coli K-12 BW25113	acrE	cytoplasmic membrane lipoprotein (NCBI)	[21,22]	no	no	NA
lambda	E coli K-12 BW25113	atpA	F0F1 ATP synthase subunit alpha (NCBI)	[21,22]	no	no	NA
lambda	E coli K-12 MG1655	bamA	part of the large multi-protein BAM complex	[10]	no	no	NA
lambda	E coli K-12 BW25113	bglG	transcriptional antiterminator of the bgl operon (NCBI)	[21,22] [10]	no	no	NA

lambda	E coli K-12 BW25113	cmk	cytidylate kinase (NCBI)	[21,22]	no	no	NA
lambda	E coli K-12 MG1655	crp	DNA-binding transcriptional dual regulator (NCBI)	[10]	no	no	NA
lambda	E coli K-12 BW25113	crr	glucose-specific PTS system enzyme IIA component (NCBI)	[21,22]	no	no	NA
lambda	E coli K-12 BW25113	cyaA	adenylate cyclase (NCBI)	[21,22][10]	Yes	no	NA
lambda	E coli K-12 BW25113	dnaJ	chaperone Hsp40, co-chaperone with DnaK (NCBI)	[21,22] [10]	Yes	no	NA
lambda	E coli K-12 MG1655	dnaK	molecular chaperone DnaK (NCBI)	[10]	Yes	no	NA
lambda	E coli K-12 MG1655	era	GTP-binding protein Era (NCBI)	[10]	no	no	NA
lambda	E coli K-12 MG1655	fmt	methionyl-tRNA formyltransferase (NCBI)	[10]	no	no	NA
lambda	E coli K-12 BW25113	fruR	DNA-binding transcriptional dual regulator (NCBI)	[21,22]	no	no	NA
lambda	E coli K-12 BW25113	fucA	L-fuculose phosphate aldolase (NCBI)	[21,22]	no	no	NA
lambda	E coli K-12 MG1655	galU	UTP—glucose-1-phosphate uridylyltransferase	[10]	Yes	no	NA
lambda	E coli K-12 BW25113	glnD	PII uridylyl-transferase (NCBI)	[21,22]	no	no	NA
lambda	E coli K-12 BW25113	gmhA/lpcA	phosphoheptose isomerase (NCBI)	[21,22]	Yes	no	NA
lambda	E coli K-12 BW25113	gmhB	hypothetical protein (NCBI)	[21,22][10]	no	no	NA
lambda	E coli K-12 BW25113	hflC	modulator for HflB protease specific for phage lambda cII repressor (NCBI)	[21,22]	no	no	NA

lambda	E coli K-12 BW25113	hflD	hypothetical protein (NCBI)	[21,22][10]	no	no	NA
lambda	E coli K-12 BW25113	hflK	modulator for HflB protease specific for phage lambda cII repressor (NCBI)	[21,22]	no	no	NA
lambda	E coli K-12 BW25113	ihfA	integration host factor subunit alpha (NCBI)	[21,22]	no	no	NA
lambda	E coli K-12 BW25113	ihfB	integration host factor subunit beta (NCBI)	[21,22]	no	no	NA
lambda	E coli K-12 BW25113	iscS	cysteine desulfurase (NCBI)	[21,22]	no	no	NA
lambda	E coli K-12 MG1655	kdsA	2-dehydro-3-deoxyphosph ooctonate aldolase (NCBI)	[10]	no	no	NA
lambda	E coli K-12 MG1655	kdsB	3-deoxy-manno-octuloson ate cytidylyltransferase (NCBI)	[10]	no	no	NA
lambda	E coli K-12 BW25113	lamB	maltoporin precursor (NCBI)	[10][23]	Yes	yes	NA
lambda	E coli K-12 MG1655	lpcA	phosphoheptose isomerase (NCBI)	[10]	Yes	no	NA
lambda	E coli K-12 MG1655	lysS	lysine tRNA synthetase, constitutive (NCBI)	[10]	no	no	NA
lambda	E coli K-12 BW25113	malI	DNA-binding transcriptional repressor (NCBI)	[21,22]	Yes	no	NA
lambda	E coli K-12 MG1655	malK	fused maltose transport subunit, ATP-binding component of ABC superfamily/regulatory protein (NCBI)	[10]	Yes	yes	NA
lambda	E coli K-12 BW25113	malT	transcriptional regulator MalT (NCBI)	[21,22][10]	Yes	yes	NA
lambda	E coli K-12 BW25113	manZ	mannose-specific enzyme IID component of PTS (NCBI)	[21,22]	no	no	NA

lambda	E coli K-12 BW25113	mnmA	tRNA-specific 2-thiouridylase	[21,22][10]	no	no	NA
lambda	E coli K-12 MG1655	mprA	DNA-binding transcriptional repressor of microcin B17 synthesis and multidrug efflux (NCBI)	[10]	no	no	NA
lambda	E coli K-12 MG1655	mtgA	monofunctional biosynthetic peptidoglycan transglycosylase (NCBI)	[10]	no	no	NA
lambda	E coli K-12 MG1655	nadD	nicotinic acid mononucleotide adenyltransferase (NCBI)	[10]	no	no	NA
lambda	E coli K-12 MG1655	nirD	nitrite reductase small subunit (NCBI)	[10]	no	no	NA
lambda	E coli K-12 BW25113	nlpI	hypothetical protein (NCBI)	[21,22]	no	no	NA
lambda	E coli K-12 MG1655	nrdR	NrdR transcriptional repressor	[10]	no	no	NA
lambda	E coli K-12 BW25113	nusB	transcription antitermination protein NusB (NCBI)	[21,22]	no	yes	NA
lambda	E coli K-12 BW25113	pabA	para-aminobenzoate synthase component II (NCBI)	[21,22]	no	no	NA
lambda	E coli K-12 BW25113	pdxH	pyridoxamine 5'-phosphate oxidase (NCBI)	[21,22]	no	no	NA
lambda	E coli K-12 BW25113	pepA	leucyl aminopeptidase (NCBI)	[21,22]	no	no	NA
lambda	E coli K-12 BW25113	pgi	glucose-6-phosphate isomerase (NCBI)	[21,22]	yes	no	NA
lambda	E coli K-12 BW25113	pgm	phosphoglucomutase (NCBI)	[21,22]	yes	no	NA
lambda	E coli K-12 BW25113	phoU	negative regulator of PhoR/PhoB	[21,22]	no	no	NA

			two-component regulator (NCBI)				
lambda	E coli K-12 MG1655	prmC	N5-glutamine S-adenosyl-L-methionine-dependent methyltransferase (NCBI)	[10]	no	no	NA
lambda	E coli K-12 MG1655	ptsI	PEP-protein phosphotransferase of PTS system (enzyme I) (NCBI)	[10]	no	no	NA
lambda	E coli K-12 MG1655	purB	adenylosuccinate lyase (NCBI)	[10]	no	no	NA
lambda	E coli K-12 BW25113	rfaH	transcriptional activator RfaH (NCBI)	[21,22]	no	no	NA
lambda	E coli K-12 MG1655	rimI	ribosomal-protein-alanine N-acetyltransferase (NCBI)	[10]	no	no	NA
lambda	E coli K-12 BW25113	rimP	conserved protein (RefSeq)	[21,22]	no	no	NA
lambda	E coli K-12 BW25113	rlmE	23S rRNA U2552 ribose 2'-O-methyltransferase, SAM-dependent (NCBI)	[21,22]	no	no	NA
lambda	E coli K-12 MG1655	rluD	23S rRNA pseudouridine synthase (NCBI)	[10]	no	no	NA
lambda	E coli K-12 MG1655	rnpA	ribonuclease P (NCBI)	[10]	no	no	NA
lambda	E coli K-12 MG1655	rnt	ribonuclease T (NCBI)	[10]	no	no	NA
lambda	E coli K-12 BW25113	rpoZ	DNA-directed RNA polymerase subunit omega (NCBI)	[21,22]	no	no	NA
lambda	E coli K-12 MG1655	rsgA	ribosome small subunit-dependent GTPase A	[10]	no	no	NA
lambda	E coli K-12 BW25113	rstA	DNA-binding response regulator in two-component regulatory system with RstB (RefSeq)	[21,22]	no	no	NA

lambda	E coli K-12 MG1655	rusA	DLP12 prophage; endonuclease RUS (NCBI)	[10]	no	no	NA
lambda	E coli K-12 BW25113	spr	predicted peptidase, outer membrane lipoprotein (NCBI)	[21,22]	no	no	NA
lambda	E coli K-12 BW25113	srmB	ATP-dependent RNA helicase (NCBI)	[21,22]	no	no	NA
lambda	E coli K-12 MG1655	sspA	stringent starvation protein A (NCBI)	[10]	no	no	NA
lambda	E coli K-12 MG1655	sspB	ClpXP protease specificity-enhancing factor (NCBI)	[10]	no	no	NA
lambda	E coli K-12 BW25113	talB	transaldolase B (NCBI)	[21,22]	no	no	NA
lambda	E coli K-12 BW25113	tpx	thiol peroxidase (NCBI)	[21,22]	no	no	NA
lambda	E coli K-12 MG1655	tsaB	N6-L-threonylcarbamoyladenine synthase, TsaB subunit	[10]	no	no	NA
lambda	E coli K-12 MG1655	tsaC	threonylcarbamoyl-AMP synthase	[10]	no	no	NA
lambda	E coli K-12 MG1655	tsaD	N6-L-threonylcarbamoyladenine synthase, TsaD subunit	[10]	no	no	NA
lambda	E coli K-12 MG1655	tsaE	N6-L-threonylcarbamoyladenine synthase, TsaE subunit	[10]	no	no	NA
lambda	E coli K-12 BW25113	tusA	sulfur transfer protein TusA	[21,22] [10]	no	no	NA
lambda	E coli K-12 MG1655	tusD	sulfurtransferase complex subunit TusD	[10]	no	no	NA
lambda	E coli K-12 BW25113	tusE	sulfur transfer protein TusE	[21,22]	no	no	NA
lambda	E coli K-12	LPS		[23]	Yes	na	NA
lambda	E coli K-12 BW25113	waaA	KDO transferase	[10]	Yes	no	NA
lambda	E coli K-12 BW25113	waaC/rfaC	ADP-heptose:LPS heptosyl transferase I (NCBI)	[21,22] [10]	Yes	no	NA

lambda	E coli K-12 MG1655	waaD/rfaD	ADP-L-glycero-D-mannoheptose-6-epimerase, NAD(P)-binding (NCBI)	[21,22][10]	Yes	no	NA
lambda	E coli K-12 MG1655	waaE/rfaE	fused heptose 7-phosphate kinase/heptose 1-phosphate adenyltransferase (NCBI)	[21,22] [10]	Yes	no	NA
lambda	E coli K-12 BW25113	waaF/rfaF	ADP-heptose:LPS heptosyltransferase II (NCBI)	[21,22][10]	Yes	no	NA
lambda	E coli K-12 BW25113	waaG	lipopolysaccharide glucosyltransferase I	[10]	no	no	NA
lambda	E coli K-12 BW25113	waaP/rfaP	lipopolysaccharide core heptose (I) kinase	[21,22]	no	no	NA
lambda	E coli K-12 MG1655	waaQ	lipopolysaccharide core heptosyltransferase 3	[10]	no	no	NA
lambda	E coli K-12 BW25113	ybeD	hypothetical protein (NCBI)	[21,22]	no	no	NA
lambda	E coli K-12 MG1655	ybeY	hypothetical protein (NCBI)	[10]	no	no	NA
lambda	E coli K-12 MG1655	ybeZ	predicted protein with nucleoside triphosphate hydrolase domain (RefSeq)	[10]	no	no	NA
lambda	E coli K-12 MG1655	ybfE	orf, hypothetical protein (VIMSS)	[10]	no	no	NA
lambda	E coli K-12 MG1655	yeaE	predicted oxidoreductase (NCBI)	[10]	no	no	NA
lambda	E coli K-12 BW25113	yecR	hypothetical protein (NCBI)	[21,22]	no	no	NA
lambda	E coli K-12 MG1655	yheO	orf, hypothetical protein (VIMSS)	[10]	no	no	NA
lambda	E coli K-12 MG1655	yjbJ	predicted stress response protein (NCBI)	[10]	no	no	NA
lambda	E coli K-12 BW25113	yneJ	predicted DNA-binding transcriptional regulator (NCBI)	[21,22]	no	no	NA

186	E coli K-12 MG1655	deaD	inducible ATP-independent RNA helicase (VIMSS)	[10]	no	no	NA
186	E coli K-12 MG1655	dnaJ	chaperone Hsp40, co-chaperone with DnaK (NCBI)	[10]	yes	no	NA
186	E coli K-12 MG1655	dnaK	molecular chaperone DnaK (NCBI)	[10]	no	no	NA
186	E coli K-12 MG1655	galU	glucose-1-phosphate uridylyltransferase (NCBI)	[10]	yes	no	NA
186	E coli K-12 MG1655	lpcA	phosphoheptose isomerase (NCBI)	[10]	yes	no	NA
186	E coli K-12 MG1655	lpxL	lipid A biosynthesis lauroyl acyltransferase (NCBI)	[10]	no	no	NA
186	E coli K-12 MG1655	lpxM	lipid A biosynthesis (KDO)2-(lauroyl)-lipid IVA acyltransferase (NCBI)	[10]	no	no	NA
186	E coli K-12 MG1655	nlpI	hypothetical protein (NCBI)	[10]	no	no	NA
186	E coli K-12 MG1655	prfA	peptide chain release factor 1 (NCBI)	[10]	no	yes	NA
186	E coli K-12 MG1655	prmC	N5-glutamine S-adenosyl-L-methionine-dependent methyltransferase (NCBI)	[10]	no	yes	NA
186	E coli K-12 MG1655	rfaH	transcriptional activator RfaH (NCBI)	[10]	yes	no	NA
186	E coli K-12 MG1655	rpoH	RNA polymerase sigma factor (NCBI)	[10]	no	no	NA
186	E coli K-12 MG1655	waaB	UDP-D-galactose:(glucosyl)lipopolysaccharide-1,6-D-galactosyltransferase	[10]	yes	yes	NA
186	E coli K-12 MG1655	waaC	ADP-heptose:LPS heptosyltransferase 1	[10]	yes	yes	NA
186	E coli K-12 MG1655	waaD/rfaD	ADP-L-glycero-D-mannoheptose 6-epimerase	[10]	yes	yes	NA

186	E coli K-12 MG1655	waaE/rfaE	fused heptose 7-phosphate kinase/heptose 1-phosphate adenyltransferase	[10]	yes	yes	NA
186	E coli K-12 MG1655	waaF	ADP-heptose—LPS heptosyltransferase 2	[10]	yes	yes	NA
186	E coli K-12 MG1655	waaG	lipopolysaccharide glucosyltransferase I	[10]	yes	yes	NA
186	E coli K-12 MG1655	waaJ	UDP-glucose:(glucosyl)LPS α-1,2-glucosyltransferase	[10]	yes	yes	NA
186	E coli K-12 MG1655	waaO	UDP-D-glucose:(glucosyl)LPS α-1,3-glucosyltransferase	[10]	yes	no	NA
186	E coli K-12 MG1655	waaP	lipopolysaccharide core heptose (I) kinase	[10]	yes	yes	NA
186	E coli K-12 MG1655	waaQ	lipopolysaccharide core heptosyltransferase 3	[10]	no	yes	NA
186	E coli K-12 MG1655	waaY	lipopolysaccharide core heptose (II) kinase	[10]	no	no	NA
186	E coli K-12 MG1655	yccU	putative CoA-binding protein with NAD(P)-binding Rossmann-fold domain	[10]	no	no	NA
186	E coli K-12 MG1655	yeaE	methylglyoxal reductase YeaE	[10]	no	no	NA
P1	E coli K-12 MG1655	galU	UTP—glucose-1-phosphate uridylyltransferase	[7,8][1–3]) [6–9] [24]	Yes	Yes	NA
P1	E coli B	LPS		[6–9][14]	Yes	NA	NA
P2	E coli K-12 MG1655	LPS		[6–9][14]	Yes	Yes	NA
P2	E coli B	LPS		[6–9][14]	Yes	NA	NA
CEV1	E coli K-12 MG1655	ompA	outer membrane protein A	[25]	No	No	NA
CEV2	E coli K-12 MG1655	fhuA	ferrichrome outer membrane transporter/phage receptor	[25,26]	Yes, both in K-12 and BL21	Yes	NA

1. Morona R, Henning U. New locus (ttr) in Escherichia coli K-12 affecting sensitivity to bacteriophage T2 and growth on oleate as the sole carbon source. J Bacteriol. 1986;168: 534–540.

2. Black PN. The fadL gene product of Escherichia coli is an outer membrane protein required for uptake of long-chain fatty acids and involved in sensitivity to bacteriophage T2. J Bacteriol. 1988;170: 2850–2854.

3. Schwartz M. Interaction of Phages with their Receptor Proteins. Virus Receptors. 1980. pp. 59–94. doi:10.1007/978-94-011-6918-9_4

4. Hantke K. Major outer membrane proteins of E. coli K12 serve as receptors for the phages T2 (protein Ia) and 434 (protein Ib). Mol Gen Genet. 1978;164: 131–135.

5. Prehm P, Jann B, Jann K, Schmidt G, Stirm S. On a bacteriophage T3 and T4 receptor region within the cell wall lipopolysaccharide of escherichia coli B. J Mol Biol. 1976;101: 277–281.

6. Lindberg AA. Bacteriophage receptors. Annu Rev Microbiol. 1973;27: 205–241.

7. Bertozzi Silva J, Storms Z, Sauvageau D. Host receptors for bacteriophage adsorption. FEMS Microbiol Lett. 2016;363. doi:10.1093/femsle/fnw002

8. Karam JD, Drake JW. Molecular biology of bacteriophage T4. American Society for Microbiology; 1994.

9. Washizaki A, Yonesaki T, Otsuka Y. Characterization of the interactions between Escherichia coli receptors, LPS and OmpC, and bacteriophage T4 long tail fibers. Microbiologyopen. 2016;5: 1003–1015.

10. Rousset F, Cui L, Siouve E, Becavin C, Depardieu F, Bikard D. Genome-wide CRISPR-dCas9 screens in E. coli identify essential genes and phage host factors. PLoS Genet. 2018;14: e1007749.

11. Cooley W, Sirotkin K, Green R, Synder L. A new gene of Escherichia coli K-12 whose product participates in T4 bacteriophage late gene expression: interaction of lit with the T4-induced polynucleotide 5’-kinase 3'-phosphatase. J Bacteriol. 1979;140: 83–91.

12. Hattman S, Fukasawa T. HOST-INDUCED MODIFICATION OF T-EVEN PHAGES DUE TO DEFECTIVE GLUCOSYLATION OF THEIR DNA. Proc Natl Acad Sci U S A. 1963;50: 297–300.

13. Wilson JH, Luftig RB, Wood WB. Interaction of bacteriophage T4 tail fiber components with a lipopolysaccharide fraction from Escherichia coli. J Mol Biol. 1970;51: 423–434.

14. Wright A, McConnell M, Kanegasaki S. Lipopolysaccharide as a Bacteriophage Receptor. Virus Receptors. 1980. pp. 27–57. doi:10.1007/978-94-011-6918-9_3

15. Heller KJ. Molecular interaction between bacteriophage and the gram-negative cell envelope. Arch Microbiol. 1992;158: 235–248.

16. Hantke K. Phage T6--colicin K receptor and nucleoside transport in Escherichia coli. FEBS Lett. 1976;70: 109–112.

17. Manning PA, Reeves P. Outer membrane proteins of Escherichia coli K-12: Isolation of a

common receptor protein for bacteriophage T6 and colicin K. MGG Molecular & General Genetics. 1978. pp. 279–286. doi:10.1007/bf00267199

18. Chamberlin M. Isolation and characterization of prototrophic mutants of Escherichia coli unable to support the intracellular growth of T7. J Virol. 1974;14: 509–516.

19. Qimron U, Marintcheva B, Tabor S, Richardson CC. Genomewide screens for Escherichia coli genes affecting growth of T7 bacteriophage. Proc Natl Acad Sci U S A. 2006;103: 19039–19044.

20. Kiino DR, Rothman-Denes LB. Genetic analysis of bacteriophage N4 adsorption. J Bacteriol. 1989;171: 4595–4602.

21. Hall BG. The rtn gene of Proteus vulgaris is actually from Escherichia coli. J Bacteriol. 1997;179: 2433–2434.

22. Maynard ND, Birch EW, Sanghvi JC, Chen L, Gutschow MV, Covert MW. A forward-genetic screen and dynamic analysis of lambda phage host-dependencies reveals an extensive interaction network and a new anti-viral strategy. PLoS Genet. 2010;6: e1001017.

23. Randall-Hazelbauer L, Schwartz M. Isolation of the Bacteriophage Lambda Receptor from Escherichia coli. Journal of Bacteriology. 1973. pp. 1436–1446. doi:10.1128/jb.116.3.1436-1446.1973

24. Franklin NC. Mutation in gal U gene of E. coli blocks phage P1 infection. Virology. 1969;38: 189–191.

25. Raya RR, Varey P, Oot RA, Dyen MR, Callaway TR, Edrington TS, et al. Isolation and characterization of a new T-even bacteriophage, CEV1, and determination of its potential to reduce Escherichia coli O157:H7 levels in sheep. Appl Environ Microbiol. 2006;72: 6405–6410.

26. Raya RR, Oot RA, Moore-Maley B, Wieland S, Callaway TR, Kutter EM, et al. Naturally resident and exogenously applied T4-like and T5-like bacteriophages can reduce Escherichia coli O157:H7 levels in sheep guts. Bacteriophage. 2011;1: 15–24.
