# Supplementary material for: High-throughput mapping of the phage resistance landscape in E. coli
Source: PLoS Biol. 2020 Oct 13;18(10):e3000877. doi: 10.1371/journal.pbio.3000877 (PMC7553319; doi:10.1371/journal.pbio.3000877)
Supplement: S1 Text — (DOCX) [file pbio.3000877.s022.docx]

**S1 Text**

**Detailed information on host factors important in phage infection and top-scoring hits for each screen per phage**

Here we summarize top scoring candidates in our RB-TnSeq, CRISPRi and Dub-seq screens in both *Escherichia coli* strains K-12 and BL21 against diverse phages. These phages include 11 canonical and well-studied Coliphages and 2 recently reported phages, which are known to kill pathogenic shiga-toxin producing *E. coli* (STEC, O157:H7), and one novel Coliphage. These 14 phages include original T-series T2, T3, T4, T5, T6, T7; N4 phage; 186 phage; Lambda cI857 phage; P1*vir* phage; P2 phage; and newer T-like phages (T6-like LZ4 phage; STEC infecting T4-like CEV1 phage; and T5-like CEV2 phage). None of these phages (except CEV1 and CEV2 phages) form plaques on smooth *E. coli* strains (for example *E. coli* O157:H7 strains, NCTC12900) that contain a complete outer core LPS (data not shown).

- The following phages were used in ***E. coli* K-12 RB-TnSeq assays**: T2, T3, T4, T5, T6, T7, N4, 186, λ cI857, P1*vir*, P2, LZ4, CEV1 and CEV2 phages; A detailed list of hits is provided in the Supplementary Data S1.
- The following phages were used in ***E. coli* K-12 CRISPRi assays**: T2, T3, T4, T5, T6, N4, 186, λ cI857, LZ4, CEV1 and CEV2 phages; A detailed list of hits is provided in the Supplementary Data S4.
- The following phages were used in ***E. coli* K-12 Dub-seq assays**: T2, T3, T4, T5, T6, T7, N4, 186, λ cI857, P1*vir*, LZ4, CEV1 and CEV2 phages; A detailed list of hits is provided in the Supplementary Data S5.
- The following phages were used in ***E. coli* BL21 RB-TnSeq assays**: T2, T3, T4, T5, T6, T7, λ cI857, P1*vir*, P2, LZ4, CEV1 and CEV2 phages; A detailed list of hits is provided in the Supplementary Data S8.
- The following phages were used in ***E. coli* BL21 Dub-seq assays**: T2, T3, T4, T5, T6, T7, λ cI857, P1*vir*, P2, LZ4, CEV1 and CEV2 phages; A detailed list of hits is provided in the Supplementary Data S9.

**Phage T2**

Phage T2 isolation and early studies have been reviewed [[4–7]](https://paperpile.com/c/Be81W5/CNLPc+NWySM+zwlTS+TiQj4). We obtained phage T2 from Calendar lab stock, UC Berkeley.

- ***Literature Summary of phage T2:***
  - Early studies pointed out through a number of different *E. coli* B strains that it is difficult to isolate mutants that are resistant to bacteriophage T2 [[4]](https://paperpile.com/c/Be81W5/CNLPc) and probably because these mutants occur at low frequencies [[8]](https://paperpile.com/c/Be81W5/VC5rt) [[9]](https://paperpile.com/c/Be81W5/tENak). Later on it was hypothesized that T2 phage probably adsorbs to more than one receptor[[9]](https://paperpile.com/c/Be81W5/tENak).
  - OmpF (protein Ia) was identified as a receptor for T2 and suggested that lipopolysaccharide was also required [[8]](https://paperpile.com/c/Be81W5/VC5rt). Later it was found that both FadL and OmpF act as T2 phage receptors in E coli K-12 [[10,11]](https://paperpile.com/c/Be81W5/WT26h+BZxLI). FadL is an outer membrane (OM), ligand gated channel that functions in the uptake of long chain (C12-C18) fatty acids, while OmpF is a general OM porin aids in the non-specific diffusion of small solutes such as sugars, ions and amino acids.
  - There are no reports on how host gene dosage impacts T2 phage infection and growth cycle.
- ***RB-TnSeq hits*: phage T2**
  - ***E. coli* K12-phage T2:**
    - Repeated RB-TnSeq fitness experiments showed that *fadL* mutants show strong fitness in the presence of T2, while *ompF* mutants did not show strong fitness scores. While this manuscript was under review, a new work using transposon sequencing to screen receptors for T2, T4, T6 and T7 phages came out [[12]](https://paperpile.com/c/Be81W5/XqEX). Our results are largely in agreement with this new study, with few differences that point to difference in the mutant library size (17,100 insertions in 3,253 bacterial genes compared to our RB-TnSeq library with 152,018 barcoded insertions in 3,728 genes). Specifically, this new work showed using low diversity transposon library and double deletion of *fadL* and *ompF* that T2 phage probably binds to both FadL and OmpF. Our assays may have missed detecting ompF because of stronger fitness by *yrfF* and *fadL* deletion strains.
    - Stronger fitness was also observed for *igaA* mutants (that participates in the Rcs phosphorelay pathway, [[13]](https://paperpile.com/c/Be81W5/WvDsP)). *igaA* is an essential gene in *E. coli* [*[14]*](https://paperpile.com/c/Be81W5/1XB9k), and our RB-TnSeq library contained 9 disruptions in *igaA*’s cytoplasmic domain which also overlapped with sgRNA target sites in our CRISPRi screen (Supplementary Fig S1). To validate that this domain is indeed dispensable for strain viability and also important in phage resistance, we successfully reconstructed the *igaA* insertion mutant. To gain further insight into the phage resistance mechanism, we performed RNA-seq analysis on the *igaA* disruption mutant. We found that multiple components of the Rcs pathway (including upregulation of *rcsA* itself) were upregulated, with 24 genes from the capsular biosynthesis-related operons *wca* and *yjb* significantly upregulated (log2FC > 2, q<0.001) (Fig. 6A). These results indicate that the *igaA* disruption mutant uncovered in this work activates colanic acid biosynthesis, leads to a mucoidy phenotype, and may be interfering with phage infection by blocking phage receptor accessibility. The IgaA residues we mutate in this work are between 18-164. Recently, it was reported that this N-terminal cytoplasmic domain of IgaA plays an important role in inhibiting Rcs in the absence of stress [[1]](https://paperpile.com/c/Be81W5/ISh1H), and the structure of this region has also been determined [[7,15]](https://paperpile.com/c/Be81W5/TiQj4+ikeSa). Distinct IgaA domains seem to coordinate different stress sensing and Rcs activation across the cytoplasmic membrane [[1]](https://paperpile.com/c/Be81W5/ISh1H).
    - We also observed a high fitness score for genes involved in LPS biosynthesis (*lpcA, waaC, waaD, waaE*) at an MOI of 1 indicating, T2 phage uses LPS as another binding region in addition to FadL. IpcA is Sedoheptulose 7-phosphate isomerase that catalyzes the first committed step in the biosynthesis of a core component of lipopolysaccharide, and gmhA mutants have LPS lacking heptose [[16]](https://paperpile.com/c/Be81W5/VrIv4); WaaD is the last enzyme in the pathway for synthesis of the ADP-heptose precursor of core LPS, and its mutant shows mucoidy phenotype[[17]](https://paperpile.com/c/Be81W5/zjtKi); WaaE catalyzes two key steps in LPS biosynthesis and disruptions to *waaE* yield heptoseless mutants; Finally, *waaC* mutants have defective LPS core heptose region or have heptoseless LPS causing a ‘deep-rough’ phenotype [[18]](https://paperpile.com/c/Be81W5/trHhW).
    - Heptoseless LPS truncations lead to destabilized membrane and shown to decrease outer membrane protein levels including FadL [[17,19–21]](https://paperpile.com/c/Be81W5/zjtKi+UaDKv+EwXfl+O7gxo)
    - Deletion of *waaC* and other genes involved in LPS core biosynthesis that lead to rough LPS induce the sigma-E extracytoplasmic stress response [[22]](https://paperpile.com/c/Be81W5/7exEh). Extracytoplasmic function sigma factor-E is known to drive the expression of small RNA RybB and which inturn downregulates *fadL* transcripts [[23]](https://paperpile.com/c/Be81W5/r4elt).
    - These results are in agreement with earlier reports on how disruptions in LPS, LPS transport pathways and OM protein biogenesis activate diverse stress responses, activate global network of small RNAs and transcription factors, and how this stress response then downregulates the level of OM porins [[13,22,24–29]](https://paperpile.com/c/Be81W5/JmSYX+bR2te+ajpGU+7exEh+eXB6b+lsVZX+bCtLU+WvDsP).
  - ***E. coli* BL21-phage T2:**
    - *fadL* mutants did not show a high fitness score in the presence of T2 and we confirmed this observation by measuring the EOP on a BL21 *fadL* deletion strain (Methods).
    - *ompF* and LPS biosynthesis genes (*waaF* and *waaG*) showed higher fitness in our screen, indicating T2 probably binds to truncated LPS R-core in BL21 and *ompF* (and probably also binds non-specifically to others, as we observe increased signal for *tsx* and *fhuA* too).
    - Absence of FadL requirement for T2 growth on BL21 is intriguing considering its 100% nucleotide identity with K-12 *fadL*, and there are no reports on differences in FadL expression between the two strains. It is possible that the conformational integrity of FadL is compromised in the absence of full length LPS (similar to OmpC, [[30]](https://paperpile.com/c/Be81W5/att6I)).
- ***K-12 CRISPRi hits:* Phage T2**
  - gRNAs targeting both *fadL* and *igaA* showed the highest fitness scores in the K-12 CRISPRi screen.
  - The putative cardiolipin transporter encoded by *yejM* and its upstream neighbor *yejL* both show enhanced fitness in the presence of T3, T4, T6, CEV1, CEV2 and LZ4 phages in the CRISPRi dataset (Fig. 3A, Fig. 3B). These genes have not been previously associated with phage resistance. Although the physiological role of cardiolipin is still emerging, it is known that cardiolipins play an important role in outer membrane protein translocation system and membrane biogenesis [[31–33]](https://paperpile.com/c/Be81W5/RfZKj+gPso1+61Sul). A recent study showed that decreased cardiolipin levels activate Rcs envelope stress response [[33–35]](https://paperpile.com/c/Be81W5/bbMaL+Tf0VK+61Sul). Our results suggest, strains with downregulation of cardiolipin transport probably display phage resistance via increased colanic acid biosynthesis.
  - Our CRISPRi screen also identified a number of *E. coli* tRNA related genes showing enhanced fitness in presence of diverse phages How the downregulation of host tRNA regions and genes encoding aminoacyl-tRNA synthetases impact host fitness, phage growth and infection cycle is not clear. We speculate that the downregulation of genes involved in protein synthesis compromises the efficient production of phage particles [[36–39]](https://paperpile.com/c/Be81W5/xUECw+qe6oe+fCjXn+ck6BE) (Joy Yang, Statistically inferring the mechanisms of phage-host interactions, PhD thesis, (2019) MIT). Follow up work is needed to systematically explore the role of host and phage encoded tRNA and aminoacyl-tRNA synthetase genes on phage growth and the cession of host translation process.
  - Among gRNAs targeting promoters, we observed a high scores for *fadL* and *igaA* (Supplementary Table S4)
  - Among gRNAs targeting transcription factor binding regions, cAMP-CRP binding sites in the promoter region of *fadL* showed high fitness scores (Supplementary Table S4).
  - gRNAs targeting neither the gene nor promoters driving *ompF* showed high scores. This result confirms our RB-TnSeq data.
- ***Dub-seq hits:* Phage T2**
  - ***E. coli* K12-phage T2:**
    - *rcsA* showed stronger fitness in the presence of T2 phage in the liquid assay format, while we observed dozens of high scoring candidates in solid assay format (in addition to *rcsA*). Strains with overexpression of *rcsA* are known to exhibit mucoidy phenotype and activate overproduction of capsule polysaccharide synthesis [[40,41]](https://paperpile.com/c/Be81W5/bQxjA+mb8vP) thereby probably prevent phage adsorption [[42]](https://paperpile.com/c/Be81W5/4iv6S). RcsA is a positive regulator of capsular polysaccharide synthesis, and is a core member of the Rcs stress response [[13,22,24–29]](https://paperpile.com/c/Be81W5/JmSYX+bR2te+ajpGU+7exEh+eXB6b+lsVZX+bCtLU+WvDsP).
    - These results are in agreement with early observations that *E. coli* K-12 strains when exposed to different phages display a mucoid phenotype [[43,44]](https://paperpile.com/c/Be81W5/pVSzy+CtXpi).
    - Solid plate assays showed higher fitness scores for Dub-seq fragments encoding these following genes:
      - *asnB,* AsnB, Asparagine synthetase B involved in catalyzing the ATP-dependent conversion of aspartate to asparagine using either glutamine or ammonium as a nitrogen source [[2,45]](https://paperpile.com/c/Be81W5/iRuyP+lQTbX).
      - *yedJ,* gene with unknown function. The genomic fragments encoding *yedJ* also encode a small RNA *rseX* (RNA suppressor of extracytoplasmic stress protease) that binds to RNA-binding protein Hfq (a global regulator) and specifically targets *ompA* and *ompC* mRNAs [[46]](https://paperpile.com/c/Be81W5/zS8Oe). RseX is not known to regulate *fadL* expression. However, overexpression of RseX has also been shown to increase biofilm formation [[3]](https://paperpile.com/c/Be81W5/X7221) indicating the role of *yedJ-rseX* locus on resistance to diverse phages.
      - *mrcA,* PBP1A, the product of the *mrcA* gene, inner membrane enzyme catalyzing the transglycosylation and transpeptidation of murein (peptidoglycan or PG) precursors during formation of the murein sacculus [[2]](https://paperpile.com/c/Be81W5/lQTbX). PBP1A role on phage resistance is not known, though we note here that *mrcA* is upstream of *igaA (yrfF)* and a key gene that shows enhanced high scores in our loss-of-function screens.
      - *yjcS* codes putative alkyl sulfatase secreted into the periplasm, and its role in T2 phage infection is not known.
      - *fimD.* FimD a member of the Fimbrial Usher Porin (FUP) family and the usher constituent in the type 1 pilus (fimbrial) chaperone-usher pathway. Role of FimD on T2 phage infection is unclear.
      - *mcrB*. McrB protein which together with McrC forms the McrBC restriction system) and is known to be capable of restriction of T-even phage DNA [[45]](https://paperpile.com/c/Be81W5/iRuyP)
  - ***E. coli* BL21-phage T2:**
    - Dub-seq fragments encoding *mlc* (*dgsA*) show increased fitness in the presence of T2 phage. Mlc controls the expression of a number of genes encoding enzymes of the *Escherichia coli* phosphotransferase and phosphoenolpyruvate systems [[2,47]](https://paperpile.com/c/Be81W5/N9PF3+lQTbX). Specifically, Mlc negatively regulates the expression of *manXYZ* which encodes a PTS transporter with broad sugar specificity including mannose, glucose, fructose, GlcNAc, and GlcN [[48–52]](https://paperpile.com/c/Be81W5/Y71H5+XYLmz+Mzc5Q+Af05I+Ra26Y). Decreased expression of PTS transporter probably impacts the LPS structure, impacts stress response pathways and may lead to the phage resistance.
    - On solid plate assays, *nirB* (encodes nitrite reductase large subunit) showed high fitness scores. How deletion in *nirB* contributes to T2 resistance is unclear.

**Phage T3**

Phage T3 belonged to the classical T-series phages [[4–6]](https://paperpile.com/c/Be81W5/CNLPc+NWySM+zwlTS). Phage T3 used in this work is from our lab stock and can infect both *E. coli* BW25113 and BL21 strains.

- ***Literature Summary on phage T3:***
  - Early studies pointed out through a number of different *E. coli* B Berkeley (BB) strains that T3 binds to *rough* LPS strains specifically LPS that terminates with heptose and has no hexose side chains [[53]](https://paperpile.com/c/Be81W5/X47wS).
  - *galU*, *waaC*, *waaG* and *waaF* mutants of *E. coli* BB showed resistance to T3 phage. These genes code enzymes involved in LPS core biosynthesis.
  - Interestingly, it was also observed that T3 does not infect all K-12 strains including BW25113 strain efficiently [[54]](https://paperpile.com/c/Be81W5/PBGXH)[[55]](https://paperpile.com/c/Be81W5/pGZwS) and the reason behind this is unclear.
  - Coevolution experiments using *E. coli* B strain (*E. coli* REL607) and T3 showed that *waaG* was the first-order resistant mutant [[56]](https://paperpile.com/c/Be81W5/mQjYZ).
  - There are no reports on how host gene dosage impacts T3 phage infection and growth cycle.
- ***RB-TnSeq hits for phage T3***
  - ***E. coli* K12-phage T3:**
    - Among the top significant hits included, *IpcA* (*gmhA*), *waaC*, *waaD*, *waaE*, *waaF* and *waaG* involved in LPS core biosynthesis. IpcA/gmhA is Sedoheptulose 7-phosphate isomerase that catalyzes the first committed step in the biosynthesis of a core component of lipopolysaccharide, and gmhA mutants have LPS lacking heptose [[16]](https://paperpile.com/c/Be81W5/VrIv4); WaaD is the last enzyme in the pathway for synthesis of the ADP-heptose precursor of core LPS, and its mutant shows mucoidy phenotype[[16,17]](https://paperpile.com/c/Be81W5/zjtKi+VrIv4); WaaE catalyzes two key steps in LPS biosynthesis and disruptions to *waaE* yield heptoseless mutants; Finally, *waaC* mutants also have defective LPS core heptose region or have heptoseless LPS causing a deep-rough phenotype [[18]](https://paperpile.com/c/Be81W5/trHhW). Deletion of *waaC* and other genes involved in LPS core biosynthesis induce the sigma-E extracytoplasmic stress response and may lead to LPS modifications [[22]](https://paperpile.com/c/Be81W5/7exEh). WaaG (UDP-glucose:(heptosyl)lipopolysaccharide α-1,3-glucosyltransferase) adds the first glucose to the HepII residue [[57]](https://paperpile.com/c/Be81W5/SY1TE). It is known that *rfaE/waaE*, *waaF*, *waaC and waaG* mutants are known to have enhanced biofilm formation, especially, *waaF* mutant overproduces the colanic acid [[58–61]](https://paperpile.com/c/Be81W5/O6KRR+pjqJk+s460O+aJewe). The reason for phage resistance is either due to enhanced biofilm formation, overproduction of colanic acid or LPS truncations is not clear.
    - Like all other phages tested in this work, in the presence of T3 phages, we observed stronger fitness for *igaA* mutants (that activates in the Rcs phosphorelay pathway). We have validated *igaA* mutant data (Main text).
    - We also noticed high fitness scoring for *wcaF* that encodes acetyltransferase involved in the biosynthesis of the extracellular polysaccharide colanic acid. It is known that *wcaF* mutants have significantly reduced colanic acid production and show impaired biofilm architecture [[62]](https://paperpile.com/c/Be81W5/NlGbv). This result of *wcaF* mutants showing high fitness in the presence of T3 phage is intriguing. Specifically, as it contradicts *igaA* mutant data, wherein activation of colanic acid production pathway shows higher fitness in the presence of diverse phages including T3. We speculate, disruption of the *wcaF* coding region with RB-TnSeq marker probably increases colanic acid production.
  - ***E. coli* BL21-phage T3:** Screens with T3 phage showed high fitness for *waaG*, *waaF* and *waaQ* confirming earlier observation about waaG as the first order resistance mutant [[56]](https://paperpile.com/c/Be81W5/mQjYZ).
- ***E. coli K-12 CRISPRi hits-phage T3***
  - gRNAs targeting *igaA, yedY* and *waaC* showed the highest fitness scores in the K-12 CRISPRi screen as well as a large number of genes involved in LPS transportation and peptidoglycan synthesis. YedY/MsrP is a periplasmic methionine sulfoxide reductase, and its role in the T3 phage infection cycle is unclear.
  - The putative cardiolipin transporter encoded by *yejM* and its upstream neighbor *yejL* both show enhanced fitness in the presence of T3, T4, T6, CEV1, CEV2 and LZ4 phages in the CRISPRi dataset (Fig. 3A and 3B). These genes have not been previously associated with phage resistance. Although the physiological role of cardiolipin is still emerging, it is known that cardiolipins play an important role in outer membrane protein translocation system and membrane biogenesis [[31–33]](https://paperpile.com/c/Be81W5/RfZKj+gPso1+61Sul). A recent study showed that decreased cardiolipin levels activate Rcs envelope stress response [[33–35]](https://paperpile.com/c/Be81W5/bbMaL+Tf0VK+61Sul). Our results suggest, strains with downregulation of cardiolipin transport probably display phage resistance via increased colanic acid biosynthesis.
  - gRNAs targeting *igaA* promoters showed the highest fitness scores confirming RB-TnSeq data and CRISPRi data on *igaA* (Supplementary Table S4).
- ***Dub-seq hits:* phage T3**
  - ***E. coli* K12-phage T3:** Genome fragments with *rcsA* showed stronger fitness in the presence of T3 phage in the liquid assay format, as well as *ompT* (outer membrane protease) and *hupB* (DNA binding global regulator). Role of ompT on T3 phage growth is not clear, but it has been shown to cleave T7 RNA polymerase [[63,64]](https://paperpile.com/c/Be81W5/UfcLn+hFGcl). The upstream region of *hupB* codes for *lon*, an ATPdependent protease that is known to degrade misfolded proteins and is involved in diverse regulatory pathways including Rcs pathway [[2]](https://paperpile.com/c/Be81W5/lQTbX).
  - Strains with overexpression of *rcsA* are known to exhibit mucoidy phenotype and enhanced colanic acid production. Overexpression of *rcsA* activates overproduction of capsule polysaccharide synthesis [[40,41,65]](https://paperpile.com/c/Be81W5/bQxjA+mb8vP+3oHoP) and probably prevents phage adsorption [[42]](https://paperpile.com/c/Be81W5/4iv6S). Early observations were made that *E. coli* K-12 strains when exposed to different phages display a mucoid phenotype [[43]](https://paperpile.com/c/Be81W5/pVSzy). RcsA is a positive regulator of capsular polysaccharide synthesis, and is a core member of the Rcs stress response [[13,22,24–29]](https://paperpile.com/c/Be81W5/JmSYX+bR2te+ajpGU+7exEh+eXB6b+lsVZX+bCtLU+WvDsP). We also observed high fitness scores for *dsrB* (protein of unknown function downstream of *rcsA*), which probably activate rcsA expression or play a role in Rcs pathway.
  - Dub-seq fragments encoding *glgC* (Glucose-1-phosphate adenylyltransferase catalyzes the rate-limiting first step in the biosynthesis of glycogen) showed higher fitness in the presence of T3. Role of glycogen biosynthesis on T3 phage infection is unclear. Overexpression of *glgC* (that encodes Glucose-1-phosphate adenylyltransferase) is known to increase glycogen accumulation [[66,67]](https://paperpile.com/c/Be81W5/M9cr0+trP2H)and titrate out the global carbon storage regulator CsrA [[68]](https://paperpile.com/c/Be81W5/LEFO1). We speculate that the interaction of GlgC and CsrA probably impacts biofilm formation [[69–72]](https://paperpile.com/c/Be81W5/bCSw7+ZbwJC+E2Fso+Rlcr2) and leads to alternations in the LPS profile [[73,74]](https://paperpile.com/c/Be81W5/3o0BT+ZQ0JW) leading to phage resistance phenotype.
  - Earlier studies have shown that alteration in outer membrane composition in *E coli* strains that lack histone-like proteins HupB. Specifically, these *hupB* mutants showed the deep-rough phenotype characterized by hypersensitivity to diverse antibiotics [[75]](https://paperpile.com/c/Be81W5/lAzs8). We speculate by overexpressing hupB, these effects are reversed and there might be overall modification of LPS and downregulation of outer membrane porins leading phage resistance. In addition, *lon* is the upstream gene to *hupB*, and is known to be involved in the regulation of diverse pathways including rcs pathway [[2]](https://paperpile.com/c/Be81W5/lQTbX). More studies are needed to understand the connection between bacterial chromosomal protein HupB and the outer membrane composition, and how that impacts fitness in the presence of phages.
  - ***E. coli* BL21-phage T3:** No stronger fitness effects were seen using the BL21 Dub-seq library (colanic acid pathway and *ompT* are missing in BL21).

**Phage T4**

Phage T4 isolation and early studies have been reviewed [[7,76,77]](https://paperpile.com/c/Be81W5/TiQj4+WZB9c+iR1tV) [[4–6]](https://paperpile.com/c/Be81W5/CNLPc+NWySM+zwlTS). We obtained phage T4 from Elizabeth Kutter Lab, The evergreen state College, Olympia.

- ***Literature Summary on Phage T4:***
  - T4 phage is one of the most well studied virulent phages. There is a huge amount of information about how cell state/growth conditions impact T4 phage receptors, replication, transcription, development and overall infection cycle [[78,79]](https://paperpile.com/c/Be81W5/DutHj+xFwOI)[[48,49,77,80,81]](https://paperpile.com/c/Be81W5/Y71H5+XYLmz+SchVT+iR1tV+AlFT2).
  - T4 phage is known to infect a limited number of *E coli* strains using two different tail fibers. Outer membrane protein *ompC* and LPS of *E. coli*-K12 are known T4 phage receptors [[82–84]](https://paperpile.com/c/Be81W5/ZCqOk+vd82I+a86UE).
  - A number of *in vitro* studies also showed how LPS might be contributing to OmpC assembly and overall helps in efficient T4 phage binding [[30,85]](https://paperpile.com/c/Be81W5/att6I+poN19).
  - The *E coli* B strain has deletion in *ompC* and T4 phage is known to use only the terminal glucose of truncated LPS [[48,86,87]](https://paperpile.com/c/Be81W5/G3M2m+KVoC4+Y71H5). These early studies on *E coli* B Berkeley showed that T4 fails to bind to *galU*, *waaC*, *waaG* and *waaF* mutants [[49,53,88]](https://paperpile.com/c/Be81W5/X47wS+bNiK8+XYLmz).
  - Recently, it was shown that T4 has no specificity for sugar sequence of LPS in the presence of *ompC*, but in the absence of *ompC*, T4 can adsorb to sugar residues on LPS [[30]](https://paperpile.com/c/Be81W5/att6I). That is, T4 shows two distinct modes of recognition, one is OmpC dependent and another one is OmpC independent.
  - It is known that T4 growth on *E coli* is impacted by the nutritional and growth phase of the host [[79]](https://paperpile.com/c/Be81W5/xFwOI). For example, an increased level of arginine is known to inhibit T4 growth while tryptophan has been shown to be necessary for T4 phage infection [[89]](https://paperpile.com/c/Be81W5/vtTQV)[[90]](https://paperpile.com/c/Be81W5/60HN2).
  - Strains overproducing colanic acid are known to show resistance to T4 phage [[91]](https://paperpile.com/c/Be81W5/vSxPk). Early studies have indicated that strains with mucoid phenotype show resistance to T4 phage [[43]](https://paperpile.com/c/Be81W5/pVSzy).
  - In a recent CRISPRi genome-wide screen study on *E coli* K-12 strain BW25113 identified key host factors important in T4 infection. These include: *ompC* and its regulator *ompR*, and LPS biosynthetic genes *waaD* and *waaF*. gRNAs targeting *igaA* also ranked high in fitness in the presence of T4 phage [[92]](https://paperpile.com/c/Be81W5/t3Lxq).
  - Another recent work described genome-wide screen of host factors important in T4 growth on *E. coli* O157, and found (other than *ompC*) 114 genes including all genes in the *sap* or susceptibility to antimicrobial peptides operon play an important role in phage infection cycle [[93]](https://paperpile.com/c/Be81W5/Wu2mh).
- ***RB-TnSeq hits: Phage T4***
  - ***E. coli* K12-phage T4:** RB-TnSeq mutants of *ompC*, and *OmpC* regulators EnvZ-OmpR two component systems encoded by *envZ* and *ompR* showed the strongest fitness in the presence of T4 phage. OmpC is a general bacterial porin and requires the EnvZ for maximal production of OmpC.
  - We also observed stronger fitness for *igaA* mutants (that participates in the Rcs phosphorelay pathway). We have validated *igaA* mutant data. IgaA is a core member of the Rcs stress response, and may also activate sigma-E response [[13,22,25–29]](https://paperpile.com/c/Be81W5/bR2te+ajpGU+7exEh+eXB6b+lsVZX+bCtLU+WvDsP).
  - In addition, we also observed high fitness scores for genes involved in LPS biosynthesis (*galU*, *lpcA, waaC, waaD, waaE, waaF*) on solid plate format assays. IpcA is Sedoheptulose 7-phosphate isomerase that catalyzes the first committed step in the biosynthesis of a core component of lipopolysaccharide, and gmhA mutants have LPS lacking heptose [[16]](https://paperpile.com/c/Be81W5/VrIv4); WaaD is the last enzyme in the pathway for synthesis of the ADP-heptose precursor of core LPS, and its mutant shows mucoidy phenotype[[17]](https://paperpile.com/c/Be81W5/zjtKi); WaaE catalyzes two key steps in LPS biosynthesis and disruptions to *waaE* yield heptoseless mutants; Finally, *waaC* mutants have defective LPS core heptose region or have heptoseless LPS causing a ‘deep-rough’ phenotype [[18]](https://paperpile.com/c/Be81W5/trHhW). Heptoseless LPS truncations lead to destabilized membrane and may decrease outer membrane protein levels including OmpC [[17,19–21]](https://paperpile.com/c/Be81W5/zjtKi+UaDKv+EwXfl+O7gxo) Deletion of *waaC* and other genes involved in LPS core biosynthesis that lead to rough LPS induce the sigma-E extracytoplasmic stress response [[22]](https://paperpile.com/c/Be81W5/7exEh). Extracytoplasmic function sigma factor-E is known to drive the expression of small RNA RybB and which inturn downregulates *ompC* transcripts [[23]](https://paperpile.com/c/Be81W5/r4elt). Reports on how disruptions in LPS, LPS transport pathways and OM protein biogenesis activate diverse stress responses, activate global network of small RNAs and transcription factors, and how that regulates the level of OM porins have been reviewed in detail [[24]](https://paperpile.com/c/Be81W5/JmSYX)

- - ***E. coli* BL21-phage T4:** Screens with T4 phage showed high fitness scores for *waaG*, *waaF* and *waaQ*, and a gene of unknown function *ybdF* that encodes DUF419 family protein (*ompC* is missing in BL21). The LPS biosynthesis gene hits are in agreement with early literature on T4 growth on *E. coli B* strains, and that the genetic determinants of T4 phage resistance seem to be same as T3 and T7 phages for *E. coli* B cells [[94]](https://paperpile.com/c/Be81W5/gdynZ)[[95]](https://paperpile.com/c/Be81W5/xqjem); [[94]](https://paperpile.com/c/Be81W5/gdynZ).
- ***E. coli*  *K-12 CRISPRi hits-phage T4:***
  - gRNAs targeting *ompC* and *igaA* showed the highest fitness scores in the presence of T4 phages in the K-12 CRISPRi screen.
  - The putative cardiolipin transporter encoded by *yejM* and its upstream neighbor *yejL* both show enhanced fitness in the presence of T3, T4, T6, CEV1, CEV2 and LZ4 phages in the CRISPRi dataset (Fig. 3A and 3B). These genes have not been previously associated with phage resistance. Although the physiological role of cardiolipin is still emerging, it is known that cardiolipins play an important role in outer membrane protein translocation system and membrane biogenesis [[31–33]](https://paperpile.com/c/Be81W5/RfZKj+gPso1+61Sul). A recent study showed that decreased cardiolipin levels activate Rcs envelope stress response [[33–35]](https://paperpile.com/c/Be81W5/bbMaL+Tf0VK+61Sul). Our results suggest, strains with downregulation of cardiolipin transport probably display phage resistance via increased colanic acid biosynthesis.
  - gRNAs targeting genes encoding components of LPS transport system (for example *lptA*, *lptC*) also showed higher fitness.
  - gRNAs targeting promoters of *ompC* and *igaA,* and LRP binding sites upstream of *ompC* (overlapping with *ompC* promoters) showed the highest fitness scores in the presence of T4 phages in the K-12 CRISPRi screen.
- ***Dub-seq hits: Phage T4***
  - ***E. coli* K12-phage T4:**  Among the top scoring candidates, Dub-seq fragments encoding *rcsA,* showed strongest fitness in the presence of T4 phage. Strains with overexpression of *rcsA* are known to exhibit mucoidy phenotype and enhanced colanic acid production. Overexpression of *rcsA* activates overproduction of capsule polysaccharide synthesis [[40,41]](https://paperpile.com/c/Be81W5/bQxjA+mb8vP) and probably prevents phage adsorption [[42]](https://paperpile.com/c/Be81W5/4iv6S). Early observations were made that *E. coli* K-12 strains when exposed to different phages display a mucoid phenotype [[43]](https://paperpile.com/c/Be81W5/pVSzy). RcsA is a positive regulator of capsular polysaccharide synthesis, and is a core member of the Rcs stress response [[24]](https://paperpile.com/c/Be81W5/JmSYX)[[13,22,25–29]](https://paperpile.com/c/Be81W5/bR2te+ajpGU+7exEh+eXB6b+lsVZX+bCtLU+WvDsP).
  - Other top scoring hits included *ygbE, ompF, ykfC, deaD and yedJ*. We followed up with *ygbE* overexpression (presented in the main text) and our EOP data confirmed that T4 phage shows strong plating defects on *ygbE* overexpressing strains. YgbE encodes DUF3561 domain-containing inner membrane protein, and has not been associated with T4 phage infection before. RNAseq of *ygbE* overexpressing revealed strong downregulation of *ompC,* the primary T4 phage receptor, though the mechanism of *ompC* downregulation in the *ygbE* overexpression strain remains to be determined. In addition to *ompC*, we also noticed a strong upregulation of *arnBCADT* operon involving lipid-A modification [[96]](https://paperpile.com/c/Be81W5/sikSh). The downregulation of *ompC* and upregulation of LPS modification genes probably impacts T4 adsorption and its growth cycle.
  - Our results on Dub-seq fragments encoding *ompF* showing stronger fitness in the presence of T4 phages can be explained by earlier observations. For example, it is known that expression of outer membrane porins *ompC* and *ompF* are regulated antagonistically by *ompR*, and increased *ompF* level does reduce T4 phage receptor *ompC* levels.
  - One of the top hits *ykfC*, encodes PF00078 domain-containing protein and part of CP4-6 prophage; Though role of YkfC in T4 phage resistance has not been reported earlier, CP4-6 prophage has been implicated in resistance to nalidixic acid [[97]](https://paperpile.com/c/Be81W5/Y1Txa)*.*
  - Our EOP validation experiments showed that *deaD* and *yedJ* overexpression failed to show increased fitness in the presence of T4 phages. Our library had only one Dub-seq fragment covering deaD and therefore got filtered out while data analysis. The RNA helicase DeaD is known to enhance expression of a number of global regulators including UvrY, a response regulator of BarA/UvrY two-component system [[98,99]](https://paperpile.com/c/Be81W5/tOaJi+la5Y5). The genomic fragments encoding *yedJ* also encode a small RNA *rseX* (RNA suppressor of extracytoplasmic stress protease) that binds to RNA-binding protein Hfq (a global regulator) and specifically targets *ompA* and *ompC* mRNAs [[46]](https://paperpile.com/c/Be81W5/zS8Oe). RseX is not known to regulate *ompC* expression. Overexpression of RseX has also been shown to increase biofilm formation [[3]](https://paperpile.com/c/Be81W5/X7221) indicating the role of *yedJ-rseX* locus on resistance to diverse phages.
  - ***E. coli* BL21-phage T4:** Dub-seq fragments encoding *mlc (dgsA)* and *argG* showed moderate fitness in the presence of T4 phages. Mlc/DgsA controls the expression of a number of genes encoding enzymes of the *Escherichia coli* phosphotransferase and phosphoenolpyruvate systems, and gets titrated away by PtsG [[2,47]](https://paperpile.com/c/Be81W5/N9PF3+lQTbX). The role of Mlc/DgsA and PtsG on T4 growth is unclear.
  - *argG* encoding Argininosuccinate synthetase enzyme that catalyzes the penultimate step of arginine biosynthesis showed strong fitness in the presence of T4 phages. It is known that increased arginine levels can inactivate T4 phages specifically [[90]](https://paperpile.com/c/Be81W5/60HN2).

**Phage T5**

Phage T5 belonged to the classical T-series phages [[4–6,78]](https://paperpile.com/c/Be81W5/CNLPc+NWySM+zwlTS+DutHj). Phage T5 used in this work is from E coli Genetic Stock center, CGSC#: 12144

- ***Literature Summary on phage T5:***
  - Early studies pointed out through a number of different *E. coli* strains that T5 phage specifically binds to FhuA involved in the transport of ferrichrome across the outer membrane [[100–102]](https://paperpile.com/c/Be81W5/wAllY+YpGfV+fnwPr); [[48,49,77,80]](https://paperpile.com/c/Be81W5/Y71H5+XYLmz+SchVT+iR1tV). There are no reports on how gene dosage impacts T5 phage infection and growth cycle.
- ***RB-TnSeq hits: phage T5***
  - ***E. coli* K12-phage T5:** *fhuA* mutants showed strong fitness in the presence of T5.
  - In addition, we also observed improved fitness *igaA* mutants (that participates in the Rcs phosphorelay pathway). We have validated *igaA* mutant data.
  - ***E. coli* BL21-phage T5:** *fhuA* mutants showed strong fitness in the presence of phage T5.
- ***E. coli* K12 *CRISPRi hits for phage T5***
  - gRNAs targeting *fhuA* and *igaA* showed the highest fitness scores in the presence of T5 phages in the K-12 CRISPRi screen.
  - gRNAs targeting *fhuA,* *igaA* and the intergenic region between *igaA* and *nudE* showed the highest fitness scores in the presence of T5 phages in the K-12 CRISPRi screen (Fig. 3).
- ***Dub-seq hits: phage T5***
  - ***E. coli* K12-phage T5:** *rcsA* showed stronger fitness in the presence of T5 phage.
  - We also observed a high fitness for Dub-seq fragments encoding rcsA loci including *dsrB* that codes for downstream (from *rcsA*) region B. Strains with overexpression of *rcsA* are known to exhibit mucoidy phenotype and enhanced colanic acid production. Overexpression of *rcsA* activates overproduction of capsule polysaccharide synthesis [[40,41]](https://paperpile.com/c/Be81W5/bQxjA+mb8vP) and probably prevents phage adsorption [[42]](https://paperpile.com/c/Be81W5/4iv6S). Early observations were made that *E. coli* K-12 strains when exposed to different phages display a mucoid phenotype [[43]](https://paperpile.com/c/Be81W5/pVSzy). RcsA is a positive regulator of capsular polysaccharide synthesis, and is a core member of the Rcs stress response [[24]](https://paperpile.com/c/Be81W5/JmSYX)[[13,22,25–29]](https://paperpile.com/c/Be81W5/bR2te+ajpGU+7exEh+eXB6b+lsVZX+bCtLU+WvDsP).
  - **BL21:** Dub-seq fragments encoding the ferrous iron uptake system (FeoB) and heme trafficking protein (YdiE) yield strong fitness in the presence T5 phage. Both *feoB* and *ydiE* belong to Fur regulon [[103]](https://paperpile.com/c/Be81W5/hTIhc). Induction of *feoB* is known to increase ferrous iron uptake and leads to

Repression of Fur regulon. This *feoB* overexpression leading to increased Fur-ferrous iron occupancy and further Fur-mediated repression of *fhuA* is known [*[104–108]*](https://paperpile.com/c/Be81W5/TS9sS+lNyD6+k8XXk+a5m9w+anqTv). Our data indicate, overexpression of *feoB* or *ydiE* increase Fur-ferrous iron occupancy and Fur-mediated repression of *fhuA* yielding stronger fitness in the presence of T5 phages. We speculate that these top-scoring candidates were missing in our K-12 Dub-seq dataset, probably because of stronger selection of *rcsA* overexpressing strains in all of our K-12 Dub-seq experiments.

**Phage T6**

Phage T6 isolation and early studies have been reviewed [[7,76,77]](https://paperpile.com/c/Be81W5/TiQj4+WZB9c+iR1tV) [[4–6]](https://paperpile.com/c/Be81W5/CNLPc+NWySM+zwlTS). We received phage T6 from ATCC 11303-B6.

- ***Literature Summary on phage T6:***
  - Early studies pointed out through a number of different *E. coli* strains that T6 phage specifically binds to Tsx involved in the uptake of nucleosides and deoxynucleosides[[109,110]](https://paperpile.com/c/Be81W5/0XfX4+hgdPr).
  - There are no reports on how gene dosage impacts T6 phage infection and growth cycle.
- ***RB-TnSeq hits:Phage T6***
  - ***E. coli* K12-phage T6:** RB-TnSeq fitness experiments showed that *tsx* mutants show strong fitness in the presence of T6.
  - Stronger fitness was observed for *igaA* mutants (that participates in the Rcs phosphorelay pathway). We have validated *igaA* mutant data.
  - ***E. coli* BL21-phage T6:** *tsx* mutants showed strong fitness in the presence of T6 phages.
- ***E. coli K-12 CRISPRi hits-phage T6***
  - gRNAs targeting *tsx* and *igaA* showed the highest fitness scores in the presence of T6 phages in the K-12 CRISPRi screen.
  - The putative cardiolipin transporter encoded by *yejM* and its upstream neighbor *yejL* both show enhanced fitness in the presence of T3, T4, T6, CEV1, CEV2 and LZ4 phages in the CRISPRi dataset (Fig. 3B). These genes have not been previously associated with phage resistance. Although the physiological role of cardiolipin is still emerging, it is known that cardiolipins play an important role in outer membrane protein translocation system and membrane biogenesis [[31–33]](https://paperpile.com/c/Be81W5/RfZKj+gPso1+61Sul). A recent study showed that decreased cardiolipin levels activate Rcs envelope stress response [[33–35]](https://paperpile.com/c/Be81W5/bbMaL+Tf0VK+61Sul). Our results suggest, strains with downregulation of cardiolipin transport probably display phage resistance via increased colanic acid biosynthesis.
  - gRNAs targeting promoters of *tsx* and *igaA* and the intergenic region between *nudE* and *igaA* showed the highest fitness scores in the presence of T6 phages in the K-12 CRISPRi screen.
- ***Dub-seq hits: phage T6***
  - ***E. coli* K12-phage T6:** *rcsA* showed stronger fitness in the presence of T6 phage. Strains with overexpression of *rcsA* are known to exhibit mucoidy phenotype and enhanced colanic acid production. Overexpression of *rcsA* activates overproduction of capsule polysaccharide synthesis [[40,41]](https://paperpile.com/c/Be81W5/bQxjA+mb8vP) and probably prevents phage adsorption [[42]](https://paperpile.com/c/Be81W5/4iv6S). Early observations were made that *E. coli* K-12 strains when exposed to different phages display a mucoid phenotype [[43]](https://paperpile.com/c/Be81W5/pVSzy). RcsA is a positive regulator of capsular polysaccharide synthesis, and is a core member of the Rcs stress response [[24]](https://paperpile.com/c/Be81W5/JmSYX)[[13,22,25–29]](https://paperpile.com/c/Be81W5/bR2te+ajpGU+7exEh+eXB6b+lsVZX+bCtLU+WvDsP).
  - We also observed increased fitness for *yedJ* (putative phosphohydrolase) containing fragments, *msrP* (formerly *yedY*) a periplasmic methionine sulfoxide reductase gene, *lit* a gene that is part of the defective prophage element e14, and ymdE a gene of unknown function. Lit overexpression is known to show resistance to T4 phages [[111,112]](https://paperpile.com/c/Be81W5/shQT3+aGhKW), though its interference with T6 adsorption and infection is not clear, it indicates that Lit plays an important role in T-even phage cycle.
  - ***E. coli* BL21-phage T6:** Dub-seq fragments encoding *dgsA* (*mlc*) show increased fitness in the presence of T6 phage. DgsA controls the expression of a number of genes encoding enzymes of the *Escherichia coli* phosphotransferase and phosphoenolpyruvate systems [[2,47]](https://paperpile.com/c/Be81W5/N9PF3+lQTbX). Dub-seq fragments encoding *ydfI*, a mannonate oxidoreductase also show higher fitness in the presence of T6 phage.

**Phage T7**

Phage T7 belonged to the classical T-series phages [[4–6]](https://paperpile.com/c/Be81W5/CNLPc+NWySM+zwlTS). Phage T7 used in this work is from E coli Genetic Stock center, CGSC#: 12146

- ***Literature Summary on phage T7:***
  - T7 phage is one of the most well studied Coliphages [[55,78,79,113,114]](https://paperpile.com/c/Be81W5/DutHj+xFwOI+pGZwS+QRfc4+bK5yq).
  - Early screen for host factors that are important for T7 infection found that the *trxA* gene that encodes for thioredoxin 1, a processivity factor crucial for T7 DNA polymerase [[115]](https://paperpile.com/c/Be81W5/l1jYW). T7 phage can bind and lyse delta *trxA* strains, though T7 phage propagation is severely compromised [[115]](https://paperpile.com/c/Be81W5/l1jYW).
  - Through a number of different *E. coli* B Berkeley (BB) strains that T3 phage, that is very closely related to T7 phage, binds to *rough* LPS strains specifically LPS that terminates with heptose and has no hexose side chains [[53]](https://paperpile.com/c/Be81W5/X47wS). Specifically, *galU*, *waaC*, *waaG* and *waaF* mutants of *E. coli* BB showed resistance to T3 phage [[53]](https://paperpile.com/c/Be81W5/X47wS).
  - Most recently, *E coli* K-12 BW25113 single gene knockout collection and ORF overexpression collection were used to study host factors that affect growth of T7 phage, and found that *trxA*, *cmk* (encodes CMP kinase that converts CMP and dCMP into CDP and dCDP), and genes encoding LPS pathway enzyme, *lpcA* (*gmhA*), *gmhB*, *waaC*, *waaD* (*rfaD*/*gmhD*), *waaE* (*rfaE*/*gmhE*), *waaF*, *waaG*, *galU* and *waaR* are essential for T7 growth [[42]](https://paperpile.com/c/Be81W5/4iv6S).
  - Overexpression of *rcsA* was also shown to resist T7 phage indicating overproduction of capsule prevents phage adsorption [[42]](https://paperpile.com/c/Be81W5/4iv6S) and confirmed by early observation that strains with mucoid phenotype [[43]](https://paperpile.com/c/Be81W5/pVSzy) or overproducing colanic acid are known to show resistance to T4 phage [[43,91]](https://paperpile.com/c/Be81W5/vSxPk+pVSzy).
  - Another study found that *E. coli* strain EV36 (a K-12/K1 hybrid) produces a K1-polysialic acid capsule and resists T7 infection [[116]](https://paperpile.com/c/Be81W5/FmdM3).
- ***RB-TnSeq hits: phage T7***
  - ***E. coli* K12-phage T7:** Among the top significant hits included, *IpcA* (*gmhA*), *waaC*, *waaD*, *waaE*, *waaF* and *waaG* involved in LPS core biosynthesis.
  - Stronger fitness was observed for *igaA* mutants (that activates in the Rcs phosphorelay pathway). We have validated *igaA* mutant data.
  - *trxA,* encoding thioredoxin 1 scored high in the solid plate assays but not in our planktonic growth assays.
  - We also noticed high fitness scoring for *wcaF* that encodes acetyltransferase involved in the biosynthesis of the extracellular polysaccharide colanic acid. It is known that *wcaF* mutants have significantly reduced colanic acid production and show impaired biofilm architecture [[62]](https://paperpile.com/c/Be81W5/NlGbv). This result of *wcaF* mutants showing high fitness in the presence of T7 phage is intriguing. Specifically, it contradicts *igaA* mutant data, wherein activation of colanic acid production pathway shows higher fitness in the presence of diverse phages including T7. We speculate, disruption of the wcaF region with RB-TnSeq marker probably increases colanic acid production.
  - Reports on how disruptions in LPS, LPS transport pathways and OM protein biogenesis activate diverse stress responses, activate global network of small RNAs and transcription factors, and how that regulates the level of OM porins and LPS homeostasis have been reviewed in detail [[24]](https://paperpile.com/c/Be81W5/JmSYX)
  - ***E. coli* BL21-phage T7:** Screens with T7 phage showed high fitness for *waaG*, *waaF* and *waaQ* coding for enzymes involved in LPS core biosynthesis.
  - In addition, we noticed high fitness for *yaeH* (DUF3461 domain-containing protein) and *ybdF* (PF04237 family protein) who have not been associated with T7 phage infection before.
- ***Dub-seq hits* -phage T7**
  - ***E. coli* K12-phage T7:** In agreement with literature, *rcsA* showed stronger fitness in the presence of T7 phage. Strains with overexpression of *rcsA* are known to exhibit mucoidy phenotype and enhanced colanic acid production. Overexpression of *rcsA* activates overproduction of capsule polysaccharide synthesis [[40,41]](https://paperpile.com/c/Be81W5/bQxjA+mb8vP) and probably prevents phage adsorption [[42]](https://paperpile.com/c/Be81W5/4iv6S). Early observations were made that *E. coli* K-12 strains when exposed to different phages display a mucoid phenotype [[43]](https://paperpile.com/c/Be81W5/pVSzy). RcsA is a positive regulator of capsular polysaccharide synthesis, and is a core member of the Rcs stress response [[24]](https://paperpile.com/c/Be81W5/JmSYX)[[13,22,25–29]](https://paperpile.com/c/Be81W5/bR2te+ajpGU+7exEh+eXB6b+lsVZX+bCtLU+WvDsP).
  - We also found stronger fitness for *ompT* that encoding outer membrane protease.
  - ***E. coli* BL21-phage T7:** Strains with Dub-seq fragments encoding *glgC* and *dgsA* show higher fitness in the BL21 Dub-seq library. *glgC* encodes Glucose-1-phosphate adenylyltransferase that catalyzes the rate-limiting first step in the biosynthesis of glycogen while DgsA controls the expression of a number of genes encoding enzymes of the *Escherichia coli* phosphotransferase and phosphoenolpyruvate systems [[2,47]](https://paperpile.com/c/Be81W5/N9PF3+lQTbX). Overexpression of *glgC* is known to increase glycogen accumulation [[62,117]](https://paperpile.com/c/Be81W5/NlGbv+PeGEK), and that *glgC* mRNA titrates out CsrA, the global carbon storage regulator [[47]](https://paperpile.com/c/Be81W5/N9PF3). The role of these enzymes and carbohydrate metabolism on T7 phage infection is unclear and needs further study.

**Phage N4**

Phage N4 isolation and early studies have been reviewed [[118,119]](https://paperpile.com/c/Be81W5/sqB9P+9B0Jz). We obtained phage N4 from Rothman-Denes Lab, The Univ of Chicago, IL.

- ***Literature Summary on phage N4:***
  - N4 phage binds to *E coli* K-12 outer membrane protein NfrA and inner membrane protein NfrB [[120–123]](https://paperpile.com/c/Be81W5/jpMbz+xRfyu+pMYyW+p7m22). The function of NfrA and NfrB is unknown.
  - A study in search of K-12 mutants that are defective in bacteriophage N4 adsorption found wecB/rffE/nfrC mutant [[120–123]](https://paperpile.com/c/Be81W5/jpMbz+xRfyu+pMYyW+p7m22). WecB encodes UDP-*N*-acetylglucosamine 2-epimerase that catalyzes the first step in UDP-*N*-acetylmannosaminuronate (UDP-ManNAcUA) biosynthesis, which is one of the building blocks for the cell surface polysaccharide enterobacterial common antigen. The role of enterobacterial common antigen on N4 phage is not known.
  - Another study found the *rtn* region when overexpressed showed resistance to N4 phage [[124,125]](https://paperpile.com/c/Be81W5/XWZXI+O4TNu). Rtn is renamed as PdeN and is a c-di-GMP phosphodiesterase, PDE [[126]](https://paperpile.com/c/Be81W5/UGNb0). There are no follow up studies reported investigating how *rtn* region is involved in N4 phage infection. In this study we confirm these early observations and also detail the entire network of PDE enzymes playing an important role in N4 phage infection.
- ***RB-TnSeq hits-Phage N4***
  - ***E. coli* K12-phage N4:** Among the top significant hits that show increased fitness in the presence of N4 phages included, *nfrA*, *nfrB*, *wecB*, *ygaH,* *yeaJ*/*dgcJ* and *pdeH*. The top scoring *nfrAB* and *wecB* confirm earlier known hits, whereas *ygaH, dgcJ* and *pdeH* have not been associated with N4 phage resistance before. *ygaH* encodes a component of the L-valine exporter complex [[126,127]](https://paperpile.com/c/Be81W5/UGNb0+zIM2K). How *ygaH* deletion and the valine exporter system impacts N4 resistance is not clear.
  - Similarly, strains with mutations in *pdeH* that encode c-di-GMP phosphodiesterase showed increased fitness in the presence of N4 phages. It is known that *pdeH* mutants show strongly reduced motility on soft agar and increased curli expression [[128,129]](https://paperpile.com/c/Be81W5/WTLyw+n3G1g). However, we would like to highlight the complexity of *pdeH* and other members of cyclic-Di-GMP network [[130]](https://paperpile.com/c/Be81W5/NvIP6).
  - Likewise, mutations in *dgcJ* that encode GGDEF domain with predicted diguanylate cyclase activity [[131]](https://paperpile.com/c/Be81W5/RCtb0) involved in the production of cyclic-Di-GMP show increased fitness in the presence of N4 phages. Interestingly, among known 12 diguanylate cyclases [[130]](https://paperpile.com/c/Be81W5/NvIP6), why only strains with *dgcJ* mutations show enhanced fitness in the presence of N4 phage is unclear. More follow up studies are needed to gain a deeper understanding of how valine levels and cyclic-Di-GMP levels (see below) regulate N4 phage infection and resistance.
  - Finally, moderate fitness was observed for *igaA* mutants (that participates in the Rcs phosphorelay pathway).
  - ***E. coli* BL21-phage N4:** Phage N4 does not bind to BL21 due to lack of *nfrA* and *nfrB*.
- ***CRISPRi hits for phage N4***
  - gRNAs targeting *nfrA*, *nfrB*, *yeaJ*, *igaA*/*yrfF* all showed the highest fitness scores in the presence of N4 phage in the K-12 CRISPRi screen, validating RB-TnSeq results.
  - In addition, we also observed a high scoring *ydfW* (unknown function, Qin prophage) *yecT (unknown function), dsrB* (downstream from *rcsA*), *glmU* and a number of other hits that show significant fitness. *glmU* encodes an enzyme that catalyzes biosynthesis of UDP-*N*-acetyl-D-glucosamine (UDP-GlcNAc), an essential precursor of cell wall peptidoglycan, lipopolysaccharide and enterobacterial common antigen.
  - We observed high fitness scores for gRNAs that target 22 *E. coli* aminoacyl-tRNA synthetases and a number of tRNA related genes as compared to no-phage control assays. Aminoacyl-tRNA synthetases play a pivotal role in protein synthesis as they catalyze aminoacylation of specific tRNAs with corresponding amino acids, and many of them are essential for cell viability. How the downregulation of host tRNA regions and genes encoding aminoacyl-tRNA synthetases impact host fitness, phage growth and infection cycle is not clear. We speculate that the downregulation of genes involved in protein synthesis compromises the efficient production of phage particles [[36]](https://paperpile.com/c/Be81W5/xUECw)[[37–39]](https://paperpile.com/c/Be81W5/qe6oe+fCjXn+ck6BE) (Joy Yang, Statistically inferring the mechanisms of phage-host interactions, PhD thesis, (2019) MIT).
  - gRNAs targeting promoter regions of *dgcJ/yeaJ*, *igaA-nudE*, *glmS*, *asnS*, *tyrS and ddpX* showed high fitness scores. Here, *asnS* and *tyrS* encode aminoacyl-tRNA synthetases: Asparagine—tRNA ligase (AsnRS) and Tyrosine-tRNA ligase (TyrRS) respectively. *glmS* is a L-glutamine:D-fructose-6-phosphate aminotransferase, that catalyzes the first step in hexosamine biosynthesis. We also observed a high scores for gRNA targeting *mrr* restriction enzyme system (methylated adenine and cytosine restriction enzyme) as well as *cheB* region, a response regulator whose mutants exhibit excessive tumbling [[2]](https://paperpile.com/c/Be81W5/lQTbX)
- ***Dub-seq hits: phage N4***
  - ***E. coli* K12-phage N4:** *rcsA* showed stronger fitness in the presence of N4 phage. That indicates, the presence of high colanic acid interferes with N4 phage interaction and its receptor. Strains with overexpression of *rcsA* are known to exhibit mucoidy phenotype and enhanced colanic acid production. Overexpression of *rcsA* activates overproduction of capsule polysaccharide synthesis [[40,41]](https://paperpile.com/c/Be81W5/bQxjA+mb8vP) and probably prevents phage adsorption [[42]](https://paperpile.com/c/Be81W5/4iv6S). Early observations were made that *E. coli* K-12 strains when exposed to different phages display a mucoid phenotype [[43]](https://paperpile.com/c/Be81W5/pVSzy). RcsA is a positive regulator of capsular polysaccharide synthesis, and is a core member of the Rcs stress response [[24]](https://paperpile.com/c/Be81W5/JmSYX)[[13,22,25–29]](https://paperpile.com/c/Be81W5/bR2te+ajpGU+7exEh+eXB6b+lsVZX+bCtLU+WvDsP).
  - We found overexpression of *pdeO(dosP)*, *pdeR (gmr)*, *pdeN (rtn)*, *pdeL* (*yahA)*, *pdeC* (*yjcC)*, *pdeB (ylaB)*, *pdeI (yliE)*, that encode cyclic-di-GMP phosphodiesterases (PDEs). The PDEs are a highly conserved group of proteins in bacteria that regulate cyclic-Di-GMP levels, a key secondary signaling molecule involved in biofilm formation, motility, virulence and other cellular processes. Though the signaling network of cyclic-di-GMP is complex, overexpression of PDEs are known to reduce c-di-GMP levels, inhibit curli and biofilm formation, while increasing cellular motility. We validated the phage resistance phenotype by estimating EOP on strains overexpressing PDEs in the presence of N4 phage. Our EOP estimations with N4 phage showed severe plating defect (EOP <8E-8) on *pdeO (dosP)*, *pdeR (gmr)*, *pdeN (rtn)*, *pdeL* (*yahA)*, *pdeB (ylaB)*, *pdeI (yliE)* overexpressing strains (Suppl Fig S3), RNAseq analysis of *pdeL* overexpression, and overexpression of the other four PDEs did not show substantially different expression of *nfrA* and *nfrB*, suggesting that N4 resistance phenotype in these instances does not appear to be via transcriptional regulation of the N4 phage receptor genes. The interconnection between PDE levels and N4 phage infection that is independent of N4 phage receptors, opens up multiple avenues including cyclic-Di-GMP regulated riboswitches regulating expression of genes important for N4 phage infection and effect of motility on N4 phage resistance.
  - *In addition, flhD, ddpX* and *yhbJ*/*rapZ* all confer resistance to N4 phage. *ddpX*, *flhD* and *yhbJ*/*rapZ*, which encode D-Ala-D-Ala dipeptidase involved in peptidoglycan biosynthesis, flagellar transcriptional regulator and an RNase adaptor protein (more discussion in the main text).
  - Our differential RNA-seq experiments (standard growth conditions, in the absence of phage) on five c-di-GMP phosphodiesterases (*pdeL*, *pdeB*, *pdeC*, *pdeN* and *pdeO*) overexpressing strains, each of which shows resistance to N4 phage (Methods) revealed large changes in the *E. coli* transcriptome relative to the wild-type BW25113 strain. N4 phage receptor genes *nfrA* and *nfrB* were not differentially expressed. The *ddpX* transcript that codes for D-Ala-D-Ala dipeptidase involved in peptidoglycan biosynthesis exhibits upregulation (log2FC = 2.11, q=2e-17) in the *pdeL* overexpression strain. Though the role of DdpX in imparting N4 phage resistance is unclear, *ddpX* is oOne of the high scoring Dub-seq screen hits in N4 phage assays is *ddpX* (Fig. 4A), though its role in imparting N4 phage resistance is not clear. We note that the fitness effect of Dub-seq fragments encoding *ddpX* might also be because of its upstream *pdeO* (*dosP*) overexpression (Supplementary Fig. S3). Similar to *pdeL* overexpression, overexpression of the other four PDEs also did not show substantially different expression of *nfrA* and *nfrB*, and therefore phage resistance phenotype in PDE overexpression strains appears to be independent of N4 phage receptor expression.

**Phage Lambda**

Phage λ discovery and early studies have been reviewed [[132]](https://paperpile.com/c/Be81W5/VYHv4) [[63]](https://paperpile.com/c/Be81W5/UfcLn)[[78,133–138]](https://paperpile.com/c/Be81W5/DXnDz+9Gljv+2bomO+SLeHX+7uFMd+Rnbxg+DutHj) [[139]](https://paperpile.com/c/Be81W5/l1efl). We obtained λ c1857 from Calendar Lab stock, UC Berkeley.

- ***Literature Summary for phage λ phage:***
  - λ is one of the well studied temperate bacteriophages that has been the foundation of the field of genetic engineering, and has been reviewed extensively [[132]](https://paperpile.com/c/Be81W5/VYHv4) [[63]](https://paperpile.com/c/Be81W5/UfcLn)[[78,133–138]](https://paperpile.com/c/Be81W5/DXnDz+9Gljv+2bomO+SLeHX+7uFMd+Rnbxg+DutHj) [[140]](https://paperpile.com/c/Be81W5/dmSbh).
  - Early studies pointed out through a number of different *E. coli* strains that bacteriophage λ binds to LamB, a sugar porin that specifically facilitates the diffusion of maltose and other maltodextrins [[141]](https://paperpile.com/c/Be81W5/hjaHf) [[142]](https://paperpile.com/c/Be81W5/XWSVO) and expressing LamB in different enteric bacteria can facilitate λ phage adsorption [[143]](https://paperpile.com/c/Be81W5/QfOyl).
  - In a series of follow up studies uncovered a number of host factors (*malT*, *malI*, *cyaA,* and genes encoding enzymes involved in LPS biosynthesis) and conditions that regulate *lamB* expression and impact λ phage infection and growth [[144–147]](https://paperpile.com/c/Be81W5/RcS01+zS5Vr+llYmK+SOWlZ). Other factors known to impact phage λ adsorption, replication, transcription, and integration into the genome were also uncovered [[78,133–138]](https://paperpile.com/c/Be81W5/DXnDz+9Gljv+2bomO+SLeHX+7uFMd+Rnbxg+DutHj).
  - The protein interaction network of bacteriophage λ with its host elucidated 62 high-confidence interactions and how these interactions shape the infection cycle [[148]](https://paperpile.com/c/Be81W5/50A2B).
  - Early observations were also made that strains with mucoid phenotype show resistance to diverse phages [[43]](https://paperpile.com/c/Be81W5/pVSzy), and overproducing colanic acid are shown to be resistant to λ phages [[43,91]](https://paperpile.com/c/Be81W5/vSxPk+pVSzy).
  - Most recently, two genome-wide loss-of-function screens, one using KEIO single gene deletion strain library [[149]](https://paperpile.com/c/Be81W5/53tT0) and another, CRISPRi library uncovered an extended network of host factors impacting host fitness in the presence of λ phage infection [[92]](https://paperpile.com/c/Be81W5/t3Lxq).
  - Though no systematic studies have been done to interrogate host factor gene dosage or overexpression effect on phage infection, it is known from detailed genetic studies that host gene dosage/overexpression might titrate out key phage proteins needed for infection cycle progression [[63,148]](https://paperpile.com/c/Be81W5/UfcLn+50A2B); [[78,133–138]](https://paperpile.com/c/Be81W5/DXnDz+9Gljv+2bomO+SLeHX+7uFMd+Rnbxg+DutHj)[[63]](https://paperpile.com/c/Be81W5/UfcLn).
  - Recently it was also shown that overexpression of small protein DicB protects *E coli* from λ phage infection [[150]](https://paperpile.com/c/Be81W5/Mkx2c).
- ***RB-TnSeq hits: phage λ***
  - ***E. coli* K12-phage λ:** Among the top significant hits that showed increased fitness in the presence of λ phages included, *lamB* encoding receptor LamB and genes encoding LamB regulators, *malT*, *cyaA* and *malI.* Disruptions in *malT*, *cyaA* and *malI* impacting LamB expression and thereby affecting λ infection agrees with the literature [[63]](https://paperpile.com/c/Be81W5/UfcLn). [[133,144–147]](https://paperpile.com/c/Be81W5/RcS01+zS5Vr+llYmK+SOWlZ+DXnDz).
  - We also observed high fitness scores for *dnaJ* and genes involved in LPS biosynthesis (*lpcA, waaC, waaD, waaE, waaF*). The effect of LPS on λ phage infection efficiency is well documented [[137,151,152]](https://paperpile.com/c/Be81W5/7uFMd+emvSP+xJpst)[[63,148]](https://paperpile.com/c/Be81W5/UfcLn+50A2B)[[133,153]](https://paperpile.com/c/Be81W5/OLfy7+DXnDz+UfcLn). Strains with truncated LPS have shown to have reduced level of LamB [[137]](https://paperpile.com/c/Be81W5/7uFMd)
  - Stronger fitness was also observed for *igaA* mutants. IgaA participates in the Rcs phosphorelay pathway, and insertion mutations in *igaA* are shown in this work to enhance colanic acid production pathway.
  - Reports on how disruptions in LPS, LPS transport pathways and OM protein biogenesis activate diverse stress responses, activate global network of small RNAs and transcription factors, and how that regulates the level of OM porins have been reviewed in detail [[24]](https://paperpile.com/c/Be81W5/JmSYX)
  - ***E. coli* BL21-phage λ:** Screens with λ phage showed high fitness for dozens of genes including top candidates: *lamB, malT, cyaA, malK, dnaJ, dnaK, rpoZ, treC, ptsG, pfkA, rne and ybdF*. *rpoZ* is a RNA polymerase subunit, *ybdF* codes for PF04237 family protein, *treC* codes for trehalose-6-hydrolase, *ptsG* codes for glucose-specific PTS enzyme IIBC component and mediates uptake with concomitant phosphorylation of glucose; *rne* codes for ribonuclease E and *pfkA* codes for 6-phosphofructokinase I, and have not been associated with λ phage infection before.
  - It is also known that PtsG and PfkA levels are regulated by Mlc, cAMP-CRP complex, and RNaseE [[2]](https://paperpile.com/c/Be81W5/lQTbX). Also, it is well documented that cAMP-CRP regulate expression of *lamB*, and cAMP-CRP levels are regulated by glycolytic flux, and also on expression of *ptsG* and *pfkA* [[2,154]](https://paperpile.com/c/Be81W5/lQTbX+CZbEr).
  - Recently, it was shown that λ-like phage HK97 (which also binds to LamB) requires the inner membrane glucose transporter protein, PtsG for its genome injection [[155]](https://paperpile.com/c/Be81W5/MpYAH).
- ***E. coli* K12 *CRISPRi hits-phage λ***
  - gRNAs targeting *lamB, malT, secE, yidC, nusB, nusG, ribE, igaA/yrfF, malK, csrA, ftsH* and a number of enzymes encoding tRNA related functions (*glnS, argU, ileY, leuS, proS)* showed the highest fitness scores in our K-12 CRISPRi screen. The full list of hits are given in Supplementary Table S5.
  - High fitness scores for *lamB*, *malT*, *igaA* and *malK* validated our RB-TnSeq results and are in agreement with the literature data [[156]](https://paperpile.com/c/Be81W5/RDYvv).
  - Mutations in genes whose products are known to be involved in transcription antitermination (*nusB, nusG*) and Sec translocon subunit E (*secE*) impact λ phage growth cycle [[156–158]](https://paperpile.com/c/Be81W5/bqCE9+7t17V+RDYvv), show higher fitness scores in our screen.
  - Another high scoring essential gene *yidC* that is known to encode a component of Sec translocon system, and participates in membrane insertion/assembly of inner membrane proteins [[159]](https://paperpile.com/c/Be81W5/xNEoH). These results indicate that the components of the Sec translocon system are crucial for λ phage infection.
  - *ribE* codes for an enzyme catalysing the penultimate step in the riboflavin biosynthesis pathway and how it impacts λ infection is unclear.
  - *csrA* codes for a protein involved in global carbon source metabolism and probably has pleiotropic effects when downregulated [[160]](https://paperpile.com/c/Be81W5/XQGwZ).
  - *ftsH* codes for a protease component and part of the FtsH/HflKC complex, degrades both soluble and inner membrane proteins, and has been shown to regulate proper membrane structure and components of LPS [[2,161,162]](https://paperpile.com/c/Be81W5/83PNw+lQTbX+6muC0). The role of *ftsH* in λ phage growth development has been well studied [[63]](https://paperpile.com/c/Be81W5/UfcLn).
  - Our observation of improved fitness in the presence of gRNA targeting genes encoding tRNA related functions (*glnS, argU, ileY, leuS, proS)* agrees with the recent CRISPRi screen on λ phage that identified a number of aminoacyl-tRNA synthetase genes showing higher fitness [[92]](https://paperpile.com/c/Be81W5/t3Lxq).
  - We also note that, in the presence of highly fit phage receptor mutants (mutants of *lamB* in this case), the strains with downregulation of most essential host functions might suffer severe resource limitation and these strains with relatively poor or neutral fitness fitness scores will be swept from the population. Follow up work is needed to systematically explore the role of host and phage encoded tRNA and aminoacyl-tRNA synthetase genes on phage growth and the cession of host translation process. A number of *E. coli* tRNA related genes showing enhanced fitness in presence of diverse phages How the downregulation of host tRNA regions and genes encoding aminoacyl-tRNA synthetases impact host fitness, phage growth and infection cycle is not clear. We speculate that the downregulation of genes involved in protein synthesis compromises the efficient production of phage particles [[36]](https://paperpile.com/c/Be81W5/xUECw)[[37–39]](https://paperpile.com/c/Be81W5/qe6oe+fCjXn+ck6BE) (Joy Yang, Statistically inferring the mechanisms of phage-host interactions, PhD thesis, (2019) MIT).
  - Among gRNAs targeting promoters, we found gRNAs targeting the intergenic region of *malE* and *malK-lamB* probably downregulate LamB expression and enhance fitness in the presence of λ phage.
  - We also observed enhanced fitness of strains with gRNA targeting the promoter of *igaA* (that probably enhances Colanic acid production and resists λ phage infection).
  - sgRNAs targeting CRP, Mlc and MalT transcription binding sites located in the intergenic promoter region of *malE* and *malK-lamB* showed the highest fitness scores in the presence of phage λ.
- ***Dub-seq hits phage λ***
  - ***E. coli* K12-phage λ:** Like all other phages used in this work, strains with Dub-seq fragments encoding *rcsA* showed strongest fitness in the presence of λ phage. Strains with overexpression of *rcsA* are known to exhibit mucoidy phenotype and enhanced colanic acid production. Overexpression of *rcsA* activates overproduction of capsule polysaccharide synthesis [[40,41]](https://paperpile.com/c/Be81W5/bQxjA+mb8vP) and probably prevents phage adsorption [[42]](https://paperpile.com/c/Be81W5/4iv6S). Early observations were made that *E. coli* K-12 strains when exposed to different phages display a mucoid phenotype [[43]](https://paperpile.com/c/Be81W5/pVSzy). RcsA is a positive regulator of capsular polysaccharide synthesis, and is a core member of the Rcs stress response [[24]](https://paperpile.com/c/Be81W5/JmSYX)[[13,22,25–29]](https://paperpile.com/c/Be81W5/bR2te+ajpGU+7exEh+eXB6b+lsVZX+bCtLU+WvDsP).
  - In addition, we observed Dub-seq fragments encoding *mlc*, *aes*, *glk*, *cpdA*, *gadY*, *sdiA*, *hupB*, number of *mal* regulon members (*malP*, *malY*, *malK*, *malE, malM*), and outer membrane proteins (*ompF*, *ompR*, *ompC*) showed high fitness scores in the presence of λ phage.
  - This is the first systematic study of host gene overexpression or increased host gene dosage effects on λ phage infection, and there is a lot of data to support some of the top hits that regulate *ma*l operon system and λ phage infection [[148]](https://paperpile.com/c/Be81W5/50A2B)[[63,133,153]](https://paperpile.com/c/Be81W5/OLfy7+DXnDz+UfcLn).
  - Mlc a global regulator of carbohydrate metabolism that is involved in the regulation of phosphoenolpyruvate and phosphotransferase system [[2,92]](https://paperpile.com/c/Be81W5/t3Lxq+lQTbX), is known to negatively regulate the maltose regulon and mannose permease system (via regulating the activator MalT), both of which are known to play a crucial role in phage λ DNA penetration and infection [[51,163,164]](https://paperpile.com/c/Be81W5/Af05I+duVND+0YJIS). *aes* encoding acetylesterase and *malY* encoding a regulatory enzyme have been shown to bind MalT, and inhibits MalT transcriptional activation activity in competition with its inducer, maltotriose (and thereby negatively regulating *lamB* expression and growth on maltose) [[145,165,166]](https://paperpile.com/c/Be81W5/Yrqp4+rhZ52+zS5Vr). Similarly, genetic interaction between MalT and Glk (glucokinase encoded by *glk*) has been demonstrated and shown that overexpression of *glk* causes reduction in *Mal* regulon [[167]](https://paperpile.com/c/Be81W5/utMDp).
  - Overexpression of *cpdA* (encoding cAMP phosphodiesterase) is known to reduce cyclic AMP and negatively influence the level of transcription of genes regulated by cAMP-CRP [[2]](https://paperpile.com/c/Be81W5/lQTbX). Role of CRP and cAMP effect on λ phage infection cycle is well studied [[63,133,153]](https://paperpile.com/c/Be81W5/OLfy7+DXnDz+UfcLn).
  - Interestingly, Dub-seq fragments encoding *mal* regulon members, maltodextrin phosphorylase (*malP)* and maltose ABC transporter components (*malK*, *malE*) showed high fitness scores in the presence of λ phage. These genes are regulated by MalT, the transcriptional activator of *lamB* expression. We speculate that these fragments probably titrate out the MalT and thereby reduce the expression of λ phage receptor *lamB*.
  - We also note, Dub-seq fragments encoding *malM-lamB* show negative fitness score, probably by enhanced *lamB* expression and increased sensitivity to λ phage. The function of MalM is unknown [[2]](https://paperpile.com/c/Be81W5/lQTbX).
  - Dub-seq fragments with small RNA *gadY* showing high fitness score, though its role in expression of *lamb* is unclear.
  - Finally, we noticed overexpression of a number of outer membrane proteins (*ompF*, *ompR*, *ompC*) show high fitness in the presence of λ phage. It is known that overexpression of OMPs induces sigma-E response and activates expression of small RNA *micA* to repress *lamB* expression[[2,168]](https://paperpile.com/c/Be81W5/lQTbX+aYSmP). These results indicate that λ phage infection is dependent on outer membrane homeostasis.
  - Earlier studies have shown alteration in outer membrane composition in *E coli* strains that lack histone-like proteins HupA and HupB. Specifically, these mutants showed the well-characterized deep-rough phenotype characterized by hypersensitivity to diverse antibiotics and also showed enhanced OmpF levels, and decreased level of small RNA *micF* [*[75]*](https://paperpile.com/c/Be81W5/lAzs8). We speculate by overexpressing hupA and hupB, these effects are reversed and there might be overall downregulation of outer membrane porins leading phage resistance. On another note, ATP dependent protease Lon is encoded upstream of hupB and is known to regulate Rcs pathway involved in colonic acid production. More studies are needed to understand the connection between bacterial chromosomal protein and the outer membrane composition.
  - ***E. coli* BL21-phage λ:** Dub-seq fragments encoding *dgsA* (*mlc*) show highest fitness in the presence of λ phage. As presented above, Mlc/DgsA negatively regulates the maltose regulon and mannose permease system (via regulating the activator MalT), both of which are known to play a crucial role in phage λ DNA penetration and infection [[51,163,164]](https://paperpile.com/c/Be81W5/Af05I+duVND+0YJIS).
  - In addition to *mlc/dgsA*, we observed overexpression of a large number of genes yielding enhanced fitness including *glk*, *cpdA*, *gadX*, *hupA*, number of *mal* regulon members (*malP*, *malY*, *malK*, *malE*), and outer membrane protein (*ompW*, *ompR*), similar to K-12 Dub-seq screen, showed high fitness scores in the presence of λ phage.
  - We noticed high scores for *bolA*, *dnaK*, *and ydfL*, where BolA is involved in regulating morphology of the cell in the presence of stress, and known to reduce cAMP-CRP when overexpressed (and thereby probably reduces LamB levels) [[2]](https://paperpile.com/c/Be81W5/lQTbX). High fitness score for *dnaK* is intriguing considering our BL21 RB-TnSeq screen also yielded *dnaK* as one of the top hits.
  - We speculate that Dub-seq fragments encoding *dnaK* might be titrating out key phage factors that are important for the growth cycle [[63]](https://paperpile.com/c/Be81W5/UfcLn). Finally, overexpression of *ydfL/pinQ* that codes for putative site specific recombinase from Qin prophage shows high fitness and its role in λ phage is unclear.

**Phage P1**

Phage P1 discovery and early studies have been reviewed [[169]](https://paperpile.com/c/Be81W5/ezQ4q). We received P1vir from Jason Gill Lab, Texas A&M University.

- ***Literature Summary:***
  - Coliphage P1 is one of the well studied temperate phage and one of the key foundational transduction tools in molecular biology and genetic engineering [[78,170–175]](https://paperpile.com/c/Be81W5/WBTTD+DutHj+8NU36+2iboa+YgQH0+0zxzi+3UMki).
  - Bacteriophage P1 phage has a wide host range. P1 infects and lysogenizes a number of enteric bacteria including *E. coli* K-12 and *E. coli* B. The host range is defined by its DNA inversion system, which rarely functions in the lytic growth [[78,170–173]](https://paperpile.com/c/Be81W5/WBTTD+DutHj+8NU36+2iboa+YgQH0).
  - Early studies found that *E. coli* LPS mutants are resistant to P1 phage. The P1 receptor has been identified to be the inner region of the LPS core [[48,176]](https://paperpile.com/c/Be81W5/DA68H+Y71H5), specifically, a terminal glucose moiety of the LPS core [[176–178]](https://paperpile.com/c/Be81W5/JimCz+xOmFP+DA68H). These studies indicated that a Glc-Hep-Hep-KDO core lacking both the terminal and the penultimate glucose residue and the branched galactose residue still functioned as PI receptor (weaker) compared to Hep-Hep-KDO core lacking all of its glucose residues showed no receptor activity.
  - Host factors that interfere with phage access to LPS core seem to inhibit phage P1. For example, *S. enterica serovar Typhimurium* has similar LPS core oligosaccharide similar to E. coli K-12, and it was found that the long O antigen interferes with P1 adsorption by obscuring the core oligosaccharide. *Gal* mutants (O-antigen mutants) of *S. enterica serovar Typhimurium* have been shown to be P1 sensitive [[179]](https://paperpile.com/c/Be81W5/QAs2O). Similarly, *E. coli* 0157:H7 is insensitive to P1 phage (though it has similar LPS core as *E. coli* K-12) while *E. coli* 0157:H7 *gal* mutants (that is they lack O-antigen) are sensitive to P1 phage [[180]](https://paperpile.com/c/Be81W5/Zseza).
  - Though P1 phage has been the workhorse of genetic engineering laboratories worldwide and has provided diverse molecular biology tools, it has not been subjected to genome-wide screens to uncover host factors important for infection and resistance. Here we used P1*vir* phage for LOF and GOF genome-wide screens in *E. coli* K-12 and BL21 strains.
- ***RB-TnSeq hits-phage P1***
  - ***E. coli* K12-phage P1:** Among the top significant hits that showed high fitness scores in the presence of P1 phages included *galU*, *pgm*, *gmhA/lpcA*, *gmhB*, *rfaD*, *rfaE*, *waaC*, *waaF*, *waaJ*, *waaO*, *waaQ*, and *wcaF*.
  - Stronger fitness was also observed for *igaA* mutants (that participate in the Rcs phosphorelay pathway).
  - Our results agree with earlier observations that *galU* mutants are resistant to P1 phage [[176]](https://paperpile.com/c/Be81W5/DA68H). GalU (UTP—glucose-1-phosphate uridylyltransferase) catalyzes the synthesis of UDP-D-glucose, a central precursor for synthesis of cell envelope components, including LPS core, colanic acid and membrane-derived oligosaccharides [[2,16,181]](https://paperpile.com/c/Be81W5/pQEBo+lQTbX+VrIv4).
  - Our results indicate that other components of LPS core are also important in efficient P1 binding and growth. For example, high scoring *lpcA*/*gmhA, gmhB, rfaD and rfaE* code for enzymes that are involved in biosynthesis of ADP-heptose precursor of core LPS, and their mutants were shown to contain lipopolysaccharides lacking heptose (rough LPS).
  - Furthermore, *pgm* that codes for phosphoglucomutase involved in the biosynthesis of UDP-a-D-glucose (a key precursor to lipid-A core biosynthesis), glycogen breakdown, metabolism of galactose and maltose also shows high fitness scores [[2]](https://paperpile.com/c/Be81W5/lQTbX). Disruptions in components involved in synthesis of core oligosaccharide-lipid A (*waaC*, *waaF*, *waaJ*, *waaO*, *waaQ*) all show resistance to P1 phage indicating the diverse possibilities of P1 phage adsorption to K-12 strains.
  - *waaJ/waaR* mutants are known to be resistant to phage Mu, which shows a similar resistance pattern to phage P1 and P2 [[59]](https://paperpile.com/c/Be81W5/pjqJk).
  - Interestingly, compared to phage 186 RB-TnSeq screen hits, our P2 and P1 phage screens did not yield high fitness scores for *waaG* and *waaP*. The reason for this difference in fitness hits between phage 186, and phage P1 and P2 is not clear.
  - It is known that *rfaD* mutants show mucoidy phenotype [[2,62,181]](https://paperpile.com/c/Be81W5/pQEBo+lQTbX+NlGbv)[[16]](https://paperpile.com/c/Be81W5/pQEBo+lQTbX+VrIv4), and *rfaE/waaE*, *waaF*, *waaC* mutants are known to have enhanced biofilm formation, especially, *waaF* mutant overproduces the colanic acid [[58–60]](https://paperpile.com/c/Be81W5/O6KRR+pjqJk+s460O). The reason for phage resistance is either due to the overproduction of colanic acid or LPS truncations is not clear. One way to deconvolute this phenotype is to study the phage resistance in the absence of colanic acid production pathway. We show from studying the P1 phage resistance in BL21 strain (that is deficient in colanic acid pathway, see below) that *waaF* mutants are resistant to P1 phage.
  - One of the interesting hits in our study from P1 phage resistance is the *wcaF* mutant. *wcaF* is located within a cluster of genes that are responsible for production of Colanic acid (CA), predicted to encode acetyltransferase. *wcaF* mutant has significantly reduced CA production and additionally shows impaired biofilm architecture [[2,62,181]](https://paperpile.com/c/Be81W5/pQEBo+lQTbX+NlGbv).
  - As we have observed with P1 phage and all other phages screened in this work, increased colanic acid production (overexpression of *rcsA* and mutants of *igaA*) increases host fitness in the presence of phages, the reason for *wcaF* mutants (that show reduced colanic acid/biofilm formation) displaying increase host fitness in the presence of phage P1 is unclear. We speculate either LPS glycoform (for example, [[182]](https://paperpile.com/c/Be81W5/pC3mj)) may also serve as phage receptor, or WcaF probably also functions in the LPS core biosynthesis pathway.
  - Reports on how disruptions in LPS, LPS transport pathways and OM protein biogenesis activate diverse stress responses, activate global network of small RNAs and transcription factors, and how that regulates the level of OM porins and LPS homeostasis have been reviewed in detail [[13,22,24–29]](https://paperpile.com/c/Be81W5/JmSYX+bR2te+ajpGU+7exEh+eXB6b+lsVZX+bCtLU+WvDsP).
  - ***E. coli* BL21-phage P1:** Among the top significant hits that showed high fitness scores in the presence of P1 phages included *waaF* and *waaG*. This result indicates that P1 phage is able to bind to BL21 despite its truncated LPS. Also, as BL21 does not have a functional *rcs* pathway and colanic acid production pathway [[183]](https://paperpile.com/c/Be81W5/gPYXx), *waaF* mutants that show resistance to P1 is not due to overproduction of colanic acid. These results define how P1 phage can recognize different LPS architectures and exhibit broad host specificity. These observations are in agreement with earlier discussions on this topic of LPS as P1 phage receptor [[48,49]](https://paperpile.com/c/Be81W5/XYLmz+Y71H5).
  - We also noticed high fitness for T6 phage receptor *tsx* and T5 phage receptor *fhuA* mutants, and the functional significance of this result is unclear. Though, how disruptions/deletions in *tsx* or *fhuA* impact LPS structure of BL21 is also not obvious, the interdependence of outer membrane proteins and LPS structure are well reported [[48,49]](https://paperpile.com/c/Be81W5/Y71H5+XYLmz).
- ***Dub-seq hits-phage P1***
  - ***E. coli* K12-phage P1:** Dub-seq fragments encoding *rcsA* and *dsrB* (downstream gene of *rcsA*) showed strongest fitness in the presence of P1 phage. These results indicate that increased colanic acid exopolysaccharide production increases *E. coli* K-12 fitness in the presence of P1 phage. Strains with overexpression of *rcsA* are known to exhibit mucoidy phenotype and enhanced colanic acid production. Overexpression of *rcsA* activates overproduction of capsule polysaccharide synthesis [[40,41]](https://paperpile.com/c/Be81W5/bQxjA+mb8vP) and probably prevents phage adsorption [[42]](https://paperpile.com/c/Be81W5/4iv6S). Early observations were made that *E. coli* K-12 strains when exposed to different phages display a mucoid phenotype [[43]](https://paperpile.com/c/Be81W5/pVSzy). RcsA is a positive regulator of capsular polysaccharide synthesis, and is a core member of the Rcs stress response [[24]](https://paperpile.com/c/Be81W5/JmSYX)[[13,22,25–29]](https://paperpile.com/c/Be81W5/bR2te+ajpGU+7exEh+eXB6b+lsVZX+bCtLU+WvDsP).
  - We also observed high scores for Dub-seq fragments encoding small RNA *micF*, *yedR-YedJ* loci and *uvrY*. Both *yedR* and *YedJ* code for inner membrane proteins with known function, while *uvrY* is a response regulator of BarA/UvrY two-component system and known to play a role in central carbon metabolism by regulating the global regulator *csrA*. The functional role of *yedR-yedJ* and and BarA/UvrY system on P1 phage infection is not clear. Recent study indicates that *uvrY* may be involved in modulating the LPS biosynthesis gene cluster and results in differential expression of LPS [[184]](https://paperpile.com/c/Be81W5/bCYQK). Though the role of *uvrY*, *csrA* and carbohydrate metabolism on P1 phage infection is unclear, it is known that mutation/downregulation of *csrA* enhances biofilm formation by regulating glycogen biosynthesis and metabolism [[185,186]](https://paperpile.com/c/Be81W5/15YwA+4nadk) and has highest concentration of LPS [[184]](https://paperpile.com/c/Be81W5/bCYQK). Though mutations in *csrA* are known to show highly pleiotropic phenotypes, the role of glycogen metabolism and *csrA* on biofilm formation is well studied [[186–189]](https://paperpile.com/c/Be81W5/50Taw+4nadk+3s1K1+pSdx2).
  - The high fitness scores for fragments encoding *micF*, a small RNA that downregulates translation of *ompF* porin in presence of phage P1 is intriguing one. As *micF* is encoded within the intergenic region of *rcsD* (activator of Rcs pathway and colanic acid biosynthesis) and *ompC*, and also contains OmpR operator sites, the resistance-causing Dub-seq fragments containing *micF* could be acting via a combination of effects that cannot be resolved in our screen.
  - Finally, high fitness score *rcsA* encoding fragments agrees with *igaA* mutant data in the RB-TnSeq screen and also agrees with observations with other phages screened in this work.
  - ***E. coli* BL21-phage P1:**  Strains with Dub-seq fragments encoding *dgsA* (*mlc*) show increased fitness in the presence of P1 phage. Mlc/DgsA controls the expression of a number of genes encoding enzymes of the *Escherichia coli* phosphotransferase and phosphoenolpyruvate systems (ecocyc.org). Specifically, Mlc negatively regulates the expression of *manXYZ* which encodes a PTS transporter with broad sugar specificity including mannose, glucose, fructose, GlcNAc, and GlcN [[48–52]](https://paperpile.com/c/Be81W5/Y71H5+XYLmz+Mzc5Q+Af05I+Ra26Y). Decreased expression of PTS transporter probably impacts the LPS structure and may lead to the phage resistance. However the effect of Mlc on LPS makes sense, we do not see high fitness scores *mlc* for all phages.

**Phage P2**

Phage P2 discovery and early studies have been reviewed [[190]](https://paperpile.com/c/Be81W5/KiErV). We received P2 phage from Calendar Lab stock, UC Berkeley.

.

- ***Literature Summary:***
  - Phage P2 belongs to a family of temperate phages that are commonly found in the genomes of gram-negative bacteria. About 30% of the *E. coli* strains in the Escherichia coli reference collection (ECOR) collection contain a P2-like phages [[191,192]](https://paperpile.com/c/Be81W5/BhNi7+l92cq).
  - The life cycle of P2 and P2-like phages has been extensively reviewed [[48,78,193,194]](https://paperpile.com/c/Be81W5/76Xhx+3Ee2w+DutHj+Y71H5).
  - Early on it was recognized that P2 tail fibers are similar to phage P1 and phage Mu, and that strains and mutants resistant to P2 are also resistant to P1 and Mu phages [[48,195,196]](https://paperpile.com/c/Be81W5/Y71H5+14ERa+AQ22x). This indicated that P1, P2 and Mu phages recognize similar moieties in the LPS core. As mentioned in the P1 section above, phage P1 needs the terminal glucose moiety of the K-12 LPS core [[176–178]](https://paperpile.com/c/Be81W5/JimCz+xOmFP+DA68H)[[48]](https://paperpile.com/c/Be81W5/DA68H+Y71H5). These studies indicated that Hep-Hep-KDO of K-12 LPS core lacking all of its glucose residues showed no P1 receptor activity.
  - Similar to P1 phage, P2 has not been subjected to genome-wide screens to uncover host factors important for infection and resistance. Here we used P2 phage for LOF and GOF genome-wide screens in *E. coli* K-12 and BL21 strains.
- ***RB-TnSeq hits: Phage P2***
  - ***E. coli* K12-phage P2:** RB-TnSeq screen hits for P2 phages were the same as P1 phage. This result is in agreement with earlier observations [[48,195,196]](https://paperpile.com/c/Be81W5/Y71H5+14ERa+AQ22x).
  - Among the top significant hits that showed high fitness scores in the presence of P2 phages included *gmhA/lpcA*, *rfaD*, *rfaE*, *waaC*, *waaF*, *waaJ*, *waaO*, *waaY, waaU, waaO, waaQ*, and *wcaF*.
  - Stronger fitness was also observed for *igaA* mutants (that participates in the Rcs phosphorelay pathway),
  - *dnaJ* (chaperone protein) and *deaD* (DEAD-box RNA helicase involved in mRNA stability and translation process) mutants also showed stronger fitness.
  - Our results indicate that all components of K-12 LPS core are important in efficient P2 binding and growth. For example, high scoring *lpcA*/*gmhA, gmhB, rfaD and rfaE* code for enzymes that are involved in biosynthesis of ADP-heptose precursor of core LPS, and their mutants were shown to contain lipopolysaccharides lacking heptose (deep rough LPS). Furthermore, *pgm* that codes for phosphoglucomutase involved in the biosynthesis of UDP-a-D-glucose (a key precursor to lipid-A core biosynthesis), glycogen breakdown, metabolism of galactose and maltose also shows high fitness scores [[2]](https://paperpile.com/c/Be81W5/lQTbX).
  - Disruptions in components involved in synthesis of core oligosaccharide-lipid A (*waaC*, *waaF*, *waaJ*, *waaO*, *waaU, waaY, waaQ*) all show resistance to P1 phage indicating the diverse possibilities of P1 phage adsorption to K-12 strains. *waaJ* activity is known to be dependent on *waaB* and *waaJ/waaR* mutants are known to be resistant to phage Mu, and that phage Mu shows a similar resistance pattern to phage P1 and P2 [[59]](https://paperpile.com/c/Be81W5/pjqJk)[[176–178]](https://paperpile.com/c/Be81W5/JimCz+xOmFP+DA68H).
  - Interestingly, compared to phage 186 RB-TnSeq screen hits, our P2 and P1 phage screens did not yield high fitness scores for *waaG* and *waaP*. The reason for this difference in fitness hits between phage 186, and phage P1 and P2 is not clear.
  - As mentioned in P1 phage section above, it is known that *rfaD* mutants show mucoidy phenotype [[2,62,181]](https://paperpile.com/c/Be81W5/pQEBo+lQTbX+NlGbv)[[16]](https://paperpile.com/c/Be81W5/pQEBo+lQTbX+VrIv4), and *rfaE/waaE*, *waaF*, *waaC* mutants are known to have enhanced biofilm formation, especially, *waaF* mutant overproduces the colanic acid [[58–60]](https://paperpile.com/c/Be81W5/O6KRR+pjqJk+s460O).
  - The reason for phage resistance is either due to the overproduction of colanic acid or LPS truncations is not clear. One way to deconvolute this phenotype is to study the phage resistance in the absence of colanic acid production pathway. We show from studying the P2 phage resistance in BL21 strain (that is deficient in colanic acid pathway, see below) that *waaF* mutants are resistant to P2 phage.
  - Like P1 phage screen hits, one of the interesting hits in our study from P2 phage resistance is the *wcaF* mutant. *wcaF* is located within a cluster of genes that are responsible for production of Colanic acid (CA), predicted to encode acetyltransferase. *wcaF* mutant has significantly reduced CA production and additionally shows impaired biofilm architecture [[2,62,181]](https://paperpile.com/c/Be81W5/pQEBo+lQTbX+NlGbv).
  - As we have observed with P2 phage and all other phages screened in this work, increased colanic acid production (overexpression of *rcsA* and mutants of *igaA*) increases host fitness in the presence of phages, the reason for *wcaF* mutants (that show reduced colanic acid/biofilm formation) displaying increase host fitness in the presence of phage P2 is unclear. We speculate either LPS glycoform (for example, [[182]](https://paperpile.com/c/Be81W5/pC3mj)) may also serve as phage receptor, or WcaF probably also functions in the LPS core biosynthesis pathway.
  - Reports on how disruptions in LPS, LPS transport pathways and OM protein biogenesis activate diverse stress responses, activate global network of small RNAs and transcription factors, and how that regulates the level of OM porins and LPS homeostasis have been reviewed in detail [[24]](https://paperpile.com/c/Be81W5/JmSYX)
  - ***E. coli* BL21-phage P2:** Same as in P1 phage screens, the top significant hits that showed high fitness scores in the presence of P2 phages included *waaF* and *waaG*. This result indicates the P2 phage is able to bind to BL21 despite its truncated LPS. These results define how P2 phage can recognize different LPS architectures and exhibit broad host specificity [[48,49]](https://paperpile.com/c/Be81W5/XYLmz+Y71H5).
- ***Dub-seq hits: Phage P2***
  - ***E. coli* BL21-phage P2:** Strains with Dub-seq fragments encoding *glgC, yhbX, gcd,* and *nhaB* show higher fitness in the presence of P2 phages and none of these enzymes have been associated with P2 phage resistance before. *nhaB* codes for Na^+^/H^+^ antiporter and *yhbX* codes for putative hydrolase, and their role in P2 infection is unclear.
  - *glgC* encodes Glucose-1-phosphate adenylyltransferase that catalyzes the rate-limiting first step in the biosynthesis of glycogen. Overexpression of *glgC* is known to increase glycogen accumulation [[62,117]](https://paperpile.com/c/Be81W5/NlGbv+PeGEK), and that *glgC* mRNA titrates out CsrA, the global carbon storage regulator impacting diverse processes [[47]](https://paperpile.com/c/Be81W5/N9PF3).
  - *gcd* codes for quinoprotein glucose dehydrogenase, and overexpression of *gcd* may alter sugar utilization preferences [[185]](https://paperpile.com/c/Be81W5/15YwA).
  - Though the role of *glgC*, *gcd*, *csrA* and carbohydrate metabolism on P2 phage infection is unclear and needs further study, it is known that mutation/downregulation of *csrA* enhances biofilm formation by regulating glycogen biosynthesis and metabolism [[185,186]](https://paperpile.com/c/Be81W5/15YwA+4nadk) and has alternations in the LPS profile [[184]](https://paperpile.com/c/Be81W5/bCYQK)[[187]](https://paperpile.com/c/Be81W5/50Taw). Though mutations in *csrA* are known to show highly pleiotropic phenotypes, the role of glycogen metabolism and *csrA* on biofilm formation is well studied [[186–189]](https://paperpile.com/c/Be81W5/50Taw+4nadk+3s1K1+pSdx2).

**Phage 186**

Phage 186 discovery and early studies have been reviewed [[197]](https://paperpile.com/c/Be81W5/VAKdo). We received 186 phage from Calendar Lab stock, UC Berkeley.

- ***Literature Summary:***
  - Bacteriophage 186 is a temperate phage and belongs to the P2 group of bacteriophages. About 30% of the *E. coli* strains in the Escherichia coli reference collection (ECOR) collection contain P2-like phages [[191,192]](https://paperpile.com/c/Be81W5/BhNi7+l92cq).
  - The life cycle of P2 and P2-like phages has been extensively reviewed [[48,78,193,194,198]](https://paperpile.com/c/Be81W5/76Xhx+3Ee2w+DutHj+Y71H5+gB8B3).
  - Morphologically phage 186 and P2 are indistinguishable, and their serological cross-reactivity suggests that their tail proteins are highly similar [[78,194,199,200]](https://paperpile.com/c/Be81W5/3Ee2w+YlX6j+DutHj+7o4uq)[[48,193]](https://paperpile.com/c/Be81W5/76Xhx+3Ee2w+DutHj+Y71H5).
  - There are some interesting differences between P2 and 186 phage including their host range [[201]](https://paperpile.com/c/Be81W5/ImMyQ).
  - Early studies by Egan and coworkers showed that phage 186 does not adsorb *E. coli* C but is capable of infecting divserse *E. coli* K-12 strains [[201]](https://paperpile.com/c/Be81W5/ImMyQ). They also found that bacterial mutants that are resistant to 186 fell into two groups, one group being sensitive to P2 phage while another resistant to P2 phage [[201]](https://paperpile.com/c/Be81W5/ImMyQ), though the reason for this difference in phage resistance profile and details of these mutant genotypes were not characterized.
  - Recently, CRISPRi genome-wide screen study on *E. coli* K-12 strain BW25113 identified a number of host factors important in 186 infection [[92]](https://paperpile.com/c/Be81W5/t3Lxq). Some of these factors included: *lpcA, galU, rfaD, rfaE, waaC, waaB, waaG, waaJ, waaO, waaY, waaP, waaQ, waaF, rfaH, lpxL, lpxM, dnaK* and *dnaJ.* Most of the top scoring genes encoded enzymes involved in LPS core biosynthesis, indicating 186 phage uses LPS-core as the receptor.
  - Here we use phage 186 to screen host factors in *E. coli* K-12 strains using both LOF and GOF methods.
- ***RB-TnSeq hits: Phage 186***
  - ***E. coli* K12-phage 186:** Among the top significant hits that showed high fitness scores in the presence of phage 186 included *dnaJ*, *galE*, *galU*, *pgm*, *gmhA/lpcA*, *gmhB*, *rfaD*, *rfaE*, *waaC*, *waaF*, *waaJ*, *waaO*, *waaB, waaP, waaG, waaQ*, *rfaH, rnb, and rep*.
  - Stronger fitness was also observed for *igaA* mutants (that participate in the Rcs phosphorelay pathway).
  - Our results agree with earlier observations that resistance to phage 186 is similar in pattern to phage P1 and P2, and largely from mutants that impact LPS architecture [[176]](https://paperpile.com/c/Be81W5/DA68H)[[78,194,199,200]](https://paperpile.com/c/Be81W5/3Ee2w+YlX6j+DutHj+7o4uq)[[201]](https://paperpile.com/c/Be81W5/ImMyQ). These results are also in agreement with the recent CRISPRi screen dataset [[92]](https://paperpile.com/c/Be81W5/t3Lxq).
  - Interestingly, compared to prior [[92]](https://paperpile.com/c/Be81W5/t3Lxq) CRISPRi screen, our screens did not yield high fitness scores for *waaG* and *waaP* in the presence of phage 186.
  - Among the top hits, GalE (UDP-glucose 4-epimerase) catalyzes a hydride transfer in the interconversion of UDP-galactose and UDP-glucose and GalU (UTP—glucose-1-phosphate uridylyltransferase) catalyzes the synthesis of UDP-D-glucose, a central precursor for synthesis of cell envelope components, including LPS core, colanic acid and membrane-derived oligosaccharides [[2,16,181]](https://paperpile.com/c/Be81W5/pQEBo+lQTbX+VrIv4).
  - Our results indicate that other deeper LPS core components are important in efficient phage 186 binding and growth. For example, high scoring *lpcA*/*gmhA, gmhB, rfaD and rfaE* code for enzymes that are involved in biosynthesis of ADP-heptose precursor of core LPS, and their mutants contain lipopolysaccharides lacking heptose (rough LPS).
  - Furthermore, *pgm* that codes for phosphoglucomutase involved in the biosynthesis of UDP-a-D-glucose (a key precursor to lipid-A core biosynthesis), glycogen breakdown, metabolism of galactose and maltose also shows high fitness scores [[2]](https://paperpile.com/c/Be81W5/lQTbX).
  - Disruptions in components involved in synthesis of core oligosaccharide-lipid A (*waaC*, *waaF*, *waaG*, *waaJ*, *waaO*, *waaB, waaP, waaQ*) all show resistance to 186 phage indicating the diverse possibilities of 186 phage adsorption to K-12 strains. *waaJ/waaR* mutants are known to be resistant to phage Mu, which shows a similar resistance pattern to phage P1 and P2, and which intern show similar resistance pattern to phage 186 [[201]](https://paperpile.com/c/Be81W5/ImMyQ)[[78,194,199,200]](https://paperpile.com/c/Be81W5/3Ee2w+YlX6j+DutHj+7o4uq)[[59]](https://paperpile.com/c/Be81W5/pjqJk).
  - In addition to genes that code for enzymes of LPS core biosynthesis pathway, we observed strong fitness scores for *rfaH* which codes for transcriptional antiterminator, a global regulator required for the production of extracellular components such as lipopolysaccharide [[202,203]](https://paperpile.com/c/Be81W5/twUnU+fQAUa)[[204,205]](https://paperpile.com/c/Be81W5/HUQ7J+B8J7h).
  - Similar to the results obtained in RB-TnSeq screen for temperate phages λ, P1 and P2, we also observed strong fitness for *dnaJ* mutants in phage 186 screen. These results agree with earlier observations with the requirement of *dnaJ* in phage λ, P1 and P2 growth [[63,206–209]](https://paperpile.com/c/Be81W5/l8dAO+Lg9EH+SZZvI+UfcLn+1V95F). DnaJ codes for cochaperone protein important in replication of diverse temperate phages and plasmids [[207,210–212]](https://paperpile.com/c/Be81W5/8es8W+Lg9EH+swt39+RVPbW).
  - *rep* encoding ATP-dependent helicase, a component of the replisome and is required for replication of a number of phages [[201]](https://paperpile.com/c/Be81W5/ImMyQ) shows high fitness in our K-12 RB-TnSeq screen of phage 186. This result is in agreement with the earlier data that phage 186 does not grow on *rep* mutants [[201]](https://paperpile.com/c/Be81W5/ImMyQ). *rep* mutants also resist phage P2 growth, as phage P2 can inject its DNA into *rep* mutants, and the phage DNA can circularize, but replication of the DNA is blocked [[213]](https://paperpile.com/c/Be81W5/ZrHug). We would like to note here that our P2 phage screen results did not return a significant fitness score for *rep* mutants.
  - Finally, rnb that codes for Ribonuclease II (RNase II), a single-strand-specific exoribonuclease important in the maturation, turnover and quality control of RNA, shows high fitness in the presence of phage 186. RNase II has been known to be important for T4 phage nucleic acid metabolism and λ phage growth cycle [[214,215]](https://paperpile.com/c/Be81W5/NorSl+lKWb9), but not associated with phage 186 growth earlier.
- ***E. coli* K12 *CRISPRi hits: phage 186***
  - gRNAs targeting components involved in synthesis of core oligosaccharide-lipid A (*waaC*, *waaG*, *waaJ*, *waaI*, *waaB, waaP, waaQ*), transcription antitermination (*nusG*) and Sec translocon subunit E (*secE*), catalytic RNA that is capable of cleaving other RNAs (rnpB), protein chain elongation factor (*tsf*) and a number of enzymes encoding tRNA related functions (*ileX, ileY, leuZ, phoU, serT, serX, thrU)* showed high fitness scores in our K-12 CRISPRi screen with phage 186. The full list of hits are given in Supplementary Table S4.
  - High fitness scores for genes coding for enzymes involved in LPS core biosynthesis validated our RB-TnSeq results and are in agreement with the earlier CRISPRi screen [[92]](https://paperpile.com/c/Be81W5/t3Lxq). It is known that mutations in genes whose products are known to be involved in transcription antitermination (*nusG*) and Sec translocon subunit E (*secE*) impact λ phage growth cycle [[156–158]](https://paperpile.com/c/Be81W5/bqCE9+7t17V+RDYvv), show higher fitness scores in phage 186 screen.
  - Our observation of improved fitness in the presence of gRNA targeting genes encoding tRNA related functions (*ileX, ileY, leuZ, phoU, serT, serX, thrU)* agree with our CRISPRi screen on λ phage that identified a number of tRNA genes showing higher fitness and also earlier results on phage λ [[92]](https://paperpile.com/c/Be81W5/t3Lxq). How the downregulation of host tRNA regions and genes encoding aminoacyl-tRNA synthetases impact host fitness, phage growth and infection cycle is not clear. We speculate that the downregulation of genes involved in protein synthesis compromises the efficient production of phage particles [[36]](https://paperpile.com/c/Be81W5/xUECw)[[37–39]](https://paperpile.com/c/Be81W5/qe6oe+fCjXn+ck6BE) (Joy Yang, Statistically inferring the mechanisms of phage-host interactions, PhD thesis, (2019) MIT).
  - Among gRNAs targeting promoters, we found gRNAs targeting promoters of LPS biosynthetic clusters (*waaP, waaAp2, rfaDp1, waaQp*), tRNAs (*thrU, serX, serT, ileX, ilvG*), and intergenic region of *cusC*-*cusF.*
- ***Dub-seq hits: phage 186***
  - ***E. coli* K12-phage 186:** Dub-seq fragments encoding *rcsA* showed stronger fitness in the presence of 186 phage. Strains with overexpression of *rcsA* are known to exhibit mucoidy phenotype and enhanced colanic acid production. Overexpression of *rcsA* activates overproduction of capsule polysaccharide synthesis [[40,41]](https://paperpile.com/c/Be81W5/bQxjA+mb8vP) and probably prevents phage adsorption [[42]](https://paperpile.com/c/Be81W5/4iv6S). Early observations were made that *E. coli* K-12 strains when exposed to different phages display a mucoid phenotype [[43]](https://paperpile.com/c/Be81W5/pVSzy). RcsA is a positive regulator of capsular polysaccharide synthesis, and is a core member of the Rcs stress response [[24]](https://paperpile.com/c/Be81W5/JmSYX)[[13,22,25–29]](https://paperpile.com/c/Be81W5/bR2te+ajpGU+7exEh+eXB6b+lsVZX+bCtLU+WvDsP).
  - In addition to *rcsA*, we observed a high fitness score for Dub-seq fragments encoding *micF* (small RNA downregulates translation of *ompF* porin), *waaS*-*waaP* (LPS biosynthesis), *gltP* (proton dependent transporter for glutamate and aspartate), *ybfC* (unknown function), *yedJ* (unknown function), *serX* (serine tRNAs)*, xapR* (transcriptional activator) and *ansP* (probable L-asparagine:proton symporter). Though, the key contribution of these top hits on the growth of phage 186 is unclear, both CRISPRi data and Dub-seq data showed high fitness score serine tRNA, *serX*, and therefore *serX* loci seems to be important for phage 186 infection. In addition to these top hits, the complete list of significant fitness hits is given in Supplementary Table S5.

**Phage CEV1**

Phage CEV1 is a recently discovered T4-like phage [[216]](https://paperpile.com/c/Be81W5/qkUFp). Phage CEV1 was kindly shared by Dr. Kutter, The evergreen state College, Olympia.

- ***Literature Summary:***
  - CEV1 phage is a T4-like myovirus that was recently isolated from sheep resistant to *E. coli* O157:H7 colonization [[216]](https://paperpile.com/c/Be81W5/qkUFp). Early characterization of CEV1 using isogenic E. coli K-12 mutants found that CEV1 uses OmpA as its receptor [[216]](https://paperpile.com/c/Be81W5/qkUFp).
  - Both *E. coli* K-12 and B strains show sensitivity to CEV1 phage. It would be interesting to see if CEV1 phage, like T4 phage, can recognize two different receptors.
  - Outer membrane protein *ompC* and LPS of *E. coli*-K12 are known T4 phage receptors [[82–84]](https://paperpile.com/c/Be81W5/ZCqOk+vd82I+a86UE). *E coli* B strain has deletion in *ompC* and T4 phage is known to use only terminal glucose of truncated LPS [[53,88]](https://paperpile.com/c/Be81W5/X47wS+bNiK8); [[217]](https://paperpile.com/c/Be81W5/1xW9P)[[48]](https://paperpile.com/c/Be81W5/Y71H5). That is, T4 shows two distinct modes of recognition, one is OmpC dependent and another one is OmpC independent.
  - Here, we used CEV1 phage to screen for key host factors important in infection using RB-TnSeq, CRISPRi and Dub-seq libraries of *E. coli*-K12, and RB-TnSeq and Dub-seq library of *E. coli* BL21
- ***RB-TnSeq hits: CEV1 phage***
  - ***E. coli* K12-phage CEV1:** RB-TnSeq mutants of *ompF*, *envZ, ompR, surA, gmhA, pgm, galU, waaE, rfaD, waaF, waaC, waaP, waaG* and *rfaH* showed strongest fitness in the presence of CEV1 phage.
  - We also observed stronger fitness for *igaA* mutants (that participates in the Rcs phosphorelay pathway).
  - *ompF* encodes major non-specific outer membrane porin (OMP) and is receptor for a number of phages.
  - EnvZ-OmpR system regulates the expression of OmpF, and therefore loss of either one of them shows high fitness. SurA is a periplasmic peptidyl-prolyl isomerase aids in proper folding of outer membrane proteins, and mutations decrease levels of OMPs including OmpA, OmpF and LamB.
  - In addition to *ompF* and its regulator the *envZ/ompR* two component signaling system, a number of genes involved in the LPS core biosynthesis pathway (*waaC*, *waaD*, *waaE*, *waaF*, *waaG*, *waaP, galU, surA* and *lpcA/gmhA*) and a regulator of genes involved in biosynthesis, assembly, and export of LPS core (*rfaH*) all showed high fitness scores (> 10) in the presence of CEV1 phage. Pgm (phosphoglucomutase) and GalU (UTP—glucose-1-phosphate uridylyltransferase) catalyzes the synthesis of UDP-D-glucose, a central precursor for synthesis of cell envelope components, including LPS core, colanic acid and membrane-derived oligosaccharides [[2,16,181]](https://paperpile.com/c/Be81W5/pQEBo+lQTbX+VrIv4).
  - Our validation experiments using CRISPRi (below) suggested that CEV1 infection proceeds by recognizing both OmpF and LPS core, and loss of either *ompF* or any component of LPS leads to a resistance phenotype.
  - Our RB-TnSeq and CRISPRi results contradict earlier observations that OmpA is CEV1 phage’s receptor [[216]](https://paperpile.com/c/Be81W5/qkUFp).
  - We propose that both OmpF and LPS core are needed for efficient CEV1 infection of *E. coli* K-12. Reports on how disruptions in LPS, LPS transport pathways and OM protein biogenesis activate diverse stress responses, activate global network of small RNAs and transcription factors, and how that regulates the level of OM porins have been reviewed in detail [[24]](https://paperpile.com/c/Be81W5/JmSYX)
  - ***E. coli* BL21-phage CEV1:** *E. coli* BL21 RB-TnSeq mutants of *ompF*, *envZ* and *ompR* showed strongest fitness in the presence of CEV1 phage and genes coding for LPS core biosynthesis.
  - Early literature on T4 phage growth on *E. coli B* strains indicated that T4 binds to LPS as BL21 has disruption in *ompC*.
  - Similarly, CEV1 phage, which showed strict requirement for OmpF and full length LPS in the K-12 screens (Fig. 2), seems to require only OmpF in BL21 infection cycle.
  - This suggests that CEV1 phage can tolerate truncated LPS of BL21 but not that of K-12.
  - Because of the OmpF requirement, CEV1 growth on BL21 also showed strict dependence on the EnvZ/OmpR two-component system, a key regulator of *ompF* expression.
- ***E. coli* K12 *CRISPRi hits: Phage CEV1***
  - gRNAs targeting *ompF* and *igaA* showed the highest fitness scores in the presence of CEV1 phages in the K-12 CRISPRi screen.
  - In addition, *yejL* (encodes (domain of unknown function) DUF1414 containing protein) and *yejM* (encodes putative cardiolipin transport protein) showed higher fitness in the presence of CEV1 phage though their role in CEV1 phage (or T4 phage) infection cycle is unclear.
  - gRNAs targeting genes encoding components of LPS transport system (for example *lptA*, *lptB, lptC*) also showed higher fitness.
  - We also find high score for *kdsC* (catalyzes synthesis of Kdo sugar that is an integral part of LPS, providing the link between lipid A and the outer core of LPS)*, and lpxA-lpxC* (LpxA and LpxC catalyze the first step in lipid A biosynthesis) indicating LPS is crucial for CEV1 binding to K-12 strain.
  - We also identify the following top scoring hits, though their direct role in imparting CEV1 phage resistance needs further study: *sokB* (small RNA blocking mokB and hokB expression) and *mdtN* (putative multidrug efflux pump).
  - gRNAs targeting promoters of *ompF* and *igaA* showed the highest fitness scores in the presence of CEV1 phage in the K-12 CRISPRi screen.
- ***Dub-seq hits: phage CEV1***
  - ***E. coli* K12-phage CEV1:**  Among the top scoring candidates, Dub-seq fragments encoding *rcsA, micF, zapE/yhcM, yhdZ, lit and kdpD* showed strongest fitness in the presence of CEV1 phage.
  - Strains with overexpression of *rcsA* are known to exhibit mucoidy phenotype and enhanced colanic acid production. Overexpression of *rcsA* activates overproduction of capsule polysaccharide synthesis [[40,41]](https://paperpile.com/c/Be81W5/bQxjA+mb8vP) and probably prevents phage adsorption [[42]](https://paperpile.com/c/Be81W5/4iv6S). Early observations were made that *E. coli* K-12 strains when exposed to different phages display a mucoid phenotype [[43]](https://paperpile.com/c/Be81W5/pVSzy). RcsA is a positive regulator of capsular polysaccharide synthesis, and is a core member of the Rcs stress response [[24]](https://paperpile.com/c/Be81W5/JmSYX)[[13,22,25–29]](https://paperpile.com/c/Be81W5/bR2te+ajpGU+7exEh+eXB6b+lsVZX+bCtLU+WvDsP).
  - In addition to *rcsA*, we observed a high fitness score for Dub-seq fragments encoding *micF*, a small RNA that downregulates translation of *ompF* porin. The role of overexpression of cell division protein ZapE and *YhdZ,* ATP-binding subunit of a putative ABC transporter on CEV1 infection cycle is unclear.
  - Another top scoring hit *kdpD* codes for the sensor member of the KdpDE two component system that contributes to K^+^ homeostasis by regulating transcription of the *kdpFABC* operon encoding a high-affinitypotassium transporting P-type ATPase [[2]](https://paperpile.com/c/Be81W5/lQTbX). Importance of potassium on T4 and T4-like phages is well documented [[218,219]](https://paperpile.com/c/Be81W5/bUtI4+gU0Ry).
  - The Dub-seq fragments encoding *lit* showed fitness score in the presence of CEV1 phage, where *lit* is part of the defective prophage element e14 [[2]](https://paperpile.com/c/Be81W5/lQTbX). Constitutive expression of *lit* is known to block T4 phage infection [[111,112]](https://paperpile.com/c/Be81W5/shQT3+aGhKW), and appears that *lit* also blocks CEV1 phage infection.
  - Earlier studies have shown alteration in outer membrane composition in *E coli* strains that lack histone-like proteins HupA and HupB. Specifically, these mutants showed the well-characterized deep-rough phenotype characterized by hypersensitivity to diverse antibiotics and also showed enhanced OmpF levels, and decreased level of small RNA *micF* [*[75]*](https://paperpile.com/c/Be81W5/lAzs8). We speculate by overexpressing hupA and hupB, these effects are reversed and there might be overall downregulation of outer membrane porins leading phage resistance. More studies are needed to understand the connection between bacterial chromosomal protein and the outer membrane composition
  - ***E. coli* BL21-phage CEV1:** Dub-seq fragments encoding *macB, ravA and mdtB* showed increased fitness in the presence of CEV1 phages. Overexpression of *macB* that codes for ATPase component of a tripartite efflux complex - MacAB-TolC showed high fitness in the presence of CEV1 phage via unknown mechanism.
  - We observed similar fitness in the presence of T4 phage. The role of ravA (regulatory ATPase) and mdtB (multidrug efflux resistance-nodulation division dubunit) on CEV1 phage infection is unclear.

**Phage CEV2**

Phage CEV2 is a recently discovered T5-like phage [[216]](https://paperpile.com/c/Be81W5/qkUFp). Phage CEV2 was kindly shared by Dr. Kutter, The evergreen state College, Olympia.

- ***Literature Summary:***
  - CEV2 phage is a T5-like myovirus that was recently isolated from sheep resistant to *E. coli* O157:H7 colonization [[216]](https://paperpile.com/c/Be81W5/qkUFp). Early characterization of CEV2 using isogenic E. coli K-12 mutants found that CEV1 uses FhuA as its receptor [[216]](https://paperpile.com/c/Be81W5/qkUFp).
  - Both *E. coli* K-12 and B strains show sensitivity to CEV2 phage. There are no reports on how gene dosage impacts CEV1 phage infection and growth cycle.
- ***RB-TnSeq hits: CEV2 phage***
  - ***E. coli* K12-phage CEV2:** *fhuA* mutants showed strong fitness in the presence of CEV2. In addition, we also observed improved fitness *igaA* mutants (that activates the colanic acid production via Rcs phosphorelay pathway).
  - Reports on how disruptions in LPS, LPS transport pathways and OM protein biogenesis activate diverse stress responses, activate global network of small RNAs and transcription factors, and how that regulates the level of OM porins have been reviewed in detail [[24]](https://paperpile.com/c/Be81W5/JmSYX)
  - **BL21:** *fhuA* mutants showed strong fitness in the presence of CEV2.
- ***E. coli* K12 *CRISPRi hits: CEV2 phage***
  - gRNAs targeting *fhuA*, *igaA* and a number of tRNA related genes and genes encoding function of membrane homeostasis showed the highest fitness scores in the presence of T5 phages in the K-12 CRISPRi screen.
  - *yejL* (encodes (domain of unknown function) DUF1414 containing protein) and *yejM* (encodes putative cardiolipin transport protein) showed higher fitness in the presence of CEV2 phage though their role in CEV2 phage (or T5 phage) infection cycle is unclear.
  - gRNAs targeting promoters of *fhuA,* *igaA* and the intergenic region between *igaA* and *nudE* showed the highest fitness scores in the presence of CEV2 phages in the K-12 CRISPRi screen.
- ***Dub-seq hits: CEV2 phage***
  - ***E. coli* K12-phage CEV2:** *rcsA* showed stronger fitness in the presence of CEV2 phage. We also observed a high fitness for Dub-seq fragments encoding *rcsA* loci including *dsrB* that codes for downstream (from *rcsA*) region B. Strains with overexpression of *rcsA* are known to exhibit mucoidy phenotype and enhanced colanic acid production. Overexpression of *rcsA* activates overproduction of capsule polysaccharide synthesis [[40,41]](https://paperpile.com/c/Be81W5/bQxjA+mb8vP) and probably prevents phage adsorption [[42]](https://paperpile.com/c/Be81W5/4iv6S). Early observations were made that *E. coli* K-12 strains when exposed to different phages display a mucoid phenotype [[43]](https://paperpile.com/c/Be81W5/pVSzy). RcsA is a positive regulator of capsular polysaccharide synthesis, and is a core member of the Rcs stress response [[24]](https://paperpile.com/c/Be81W5/JmSYX)[[13,22,25–29]](https://paperpile.com/c/Be81W5/bR2te+ajpGU+7exEh+eXB6b+lsVZX+bCtLU+WvDsP).
  - Earlier studies have shown alteration in outer membrane composition in *E coli* strains that lack histone-like proteins HupA and HupB. Specifically, these mutants showed the well-characterized deep-rough phenotype characterized by hypersensitivity to diverse antibiotics and also showed enhanced OmpF levels, and decreased level of small RNA *micF* [*[75]*](https://paperpile.com/c/Be81W5/lAzs8). We speculate by overexpressing hupA and hupB, these effects are reversed and there might be overall downregulation of outer membrane porins leading phage resistance. More studies are needed to understand the connection between bacterial HupAB and the outer membrane composition
  - ***E. coli* BL21-phage CEV2:** Dub-seq fragments encoding the ferrous iron uptake system (FeoB) and heme trafficking protein (YdiE) yield strong fitness in the presence of CEV2 phage (and also T5 phage).
  - Both *feoB* and *ydiE* belong to Fur regulon [[103]](https://paperpile.com/c/Be81W5/hTIhc). Induction of *feoB* is known to increase ferrous iron uptake and leads to repression of Fur regulon. This *feoB* overexpression leading to increased Fur-ferrous iron occupancy and further Fur-mediated repression of *fhuA* is known [*[104–108]*](https://paperpile.com/c/Be81W5/TS9sS+lNyD6+k8XXk+a5m9w+anqTv).
  - Our data indicate, overexpression of *feoB* or *ydiE* increases Fur-ferrous iron occupancy and Fur-mediated repression of *fhuA* yielding stronger fitness in the presence of T5-like phages. As mentioned earlier, we speculate that these top-scoring candidates were missing in our K-12 Dub-seq dataset, probably because of stronger selection of *rcsA* overexpressing strains in all of our K-12 Dub-seq experiments.

**Phage LZ4**

This T-even phage LZ4 was isolated from Denver Zoo from Hyena by Sean Eddy (Introns in the T-even bacteriophages (PhD thesis, (1991) University of Colorado). [OCLC](https://en.wikipedia.org/wiki/OCLC) [28253022](https://www.worldcat.org/oclc/28253022). [ProQuest](https://en.wikipedia.org/wiki/ProQuest) [303935681](https://search.proquest.com/docview/303935681). Phage LZ4 was kindly shared by Dr. Kutter, The evergreen state College, Olympia.

- ***Literature Summary:***
  - LZ4 phage is a predicted T6-like phage [[220]](https://paperpile.com/c/Be81W5/6E8W1). This phage and its host receptor remained uncharacterized in detail except that it was indicated that LZ4 might be binding to Tsx (Sean Eddy thesis, (Introns in the T-even bacteriophages (PhD thesis, (1991) University of Colorado). [OCLC](https://en.wikipedia.org/wiki/OCLC) [28253022](https://www.worldcat.org/oclc/28253022). [ProQuest](https://en.wikipedia.org/wiki/ProQuest) [303935681](https://search.proquest.com/docview/303935681), Table 3.2 and 3.3). T6 phage specifically binds to Tsx involved in the uptake of nucleosides and deoxynucleosides [[109,110]](https://paperpile.com/c/Be81W5/0XfX4+hgdPr).
  - LZ4 phage is the least characterized phage in our dataset.
- ***RB-TnSeq hits: phage LZ4***
  - ***E. coli* K12-phage LZ4:** RB-TnSeq fitness experiments showed that *tsx* mutants show strong fitness in the presence of LZ4. Stronger fitness was also observed for genes coding for enzymes involved in the LPS biosynthesis (*galU, lpcA, waaC, waaD, waaE, waaF*) and Rcs pathway activating *igaA* mutants.
  - These results indicate that the LZ4 phage specifically binds to Tsx, and its interaction with core LPS probably increases the efficiency of infection.
  - Reports on how disruptions in LPS, LPS transport pathways and OM protein biogenesis activate diverse stress responses, activate global network of small RNAs and transcription factors, and how that regulates the level of OM porins have been reviewed in detail [[24]](https://paperpile.com/c/Be81W5/JmSYX)
  - ***E. coli* BL21-phage LZ4:** *tsx* and *waaG* mutants showed strong fitness in the presence of LZ4 phages.
- ***E. coli* K12 *CRISPRi hits: Phage LZ4***
  - gRNAs targeting *tsx*, *igaA, ompF and mdtN* showed the highest fitness scores in the presence of LZ4 phages in the K-12 CRISPRi screen.
  - OmpF and MdtN role in imparting higher fitness in the presence of LZ4 is unclear.
  - In addition, *yejL* (encodes (domain of unknown function) DUF1414 containing protein), *yejM* (encodes putative cardiolipin transport protein) showed higher fitness in the presence of LZ4 phages with unknown mechanism.
  - gRNAs targeting promoters of *igaA* showed the highest fitness scores in the presence of LZ4 phages in the K-12 CRISPRi screen.
- ***Dub-seq hits: phage LZ4***
  - ***E. coli* K12-phage LZ4:** *rcsA* showed stronger fitness in the presence of LZ4 phages. Strains with overexpression of *rcsA* are known to exhibit mucoidy phenotype and enhanced colanic acid production. Overexpression of *rcsA* activates overproduction of capsule polysaccharide synthesis [[40,41]](https://paperpile.com/c/Be81W5/bQxjA+mb8vP) and probably prevents phage adsorption [[42]](https://paperpile.com/c/Be81W5/4iv6S). Early observations were made that *E. coli* K-12 strains when exposed to different phages display a mucoid phenotype [[43]](https://paperpile.com/c/Be81W5/pVSzy). RcsA is a positive regulator of capsular polysaccharide synthesis, and is a core member of the Rcs stress response [[24]](https://paperpile.com/c/Be81W5/JmSYX)[[13,22,25–29]](https://paperpile.com/c/Be81W5/bR2te+ajpGU+7exEh+eXB6b+lsVZX+bCtLU+WvDsP).
  - We also observed increased fitness for *lit* gene that is part of the defective prophage element e14. Lit overexpression is known to show resistance to T4 phages [[111,112]](https://paperpile.com/c/Be81W5/shQT3+aGhKW). We also observed a high score for *lit* in the presence of T6 and CEV1 phages in the K-12 Dub-seq screen, though its interference with LZ4 adsorption and infection is not clear. This result indicates that Lit plays an important role in the T-even phage infection cycle.
  - ***E. coli* BL21-phage LZ4:** Dub-seq fragments encoding *dgsA* (*mlc*), yfeC (unknown function) and ompX show increased fitness in the presence of LZ4 phage. DgsA controls the expression of a number of genes encoding enzymes of the *Escherichia coli* phosphotransferase and phosphoenolpyruvate systems [[2,47]](https://paperpile.com/c/Be81W5/N9PF3+lQTbX). We speculate Dub-seq fragments encoding *ompX* probably stimulates sigma E response and downregulates the tsx expression.

**REFERENCES**

1. [Hussein NA, Cho S-H, Laloux G, Siam R, Collet J-F. Distinct domains of Escherichia coli IgaA connect envelope stress sensing and down-regulation of the Rcs phosphorelay across subcellular compartments. PLoS Genet. 2018;14: e1007398.](http://paperpile.com/b/Be81W5/ISh1H)

2. [Keseler IM, Mackie A, Santos-Zavaleta A, Billington R, Bonavides-Martínez C, Caspi R, et al. The EcoCyc database: reflecting new knowledge about Escherichia coli K-12. Nucleic Acids Res. 2017;45: D543–D550.](http://paperpile.com/b/Be81W5/lQTbX)

3. [Bak G, Lee J, Suk S, Kim D, Young Lee J, Kim K-S, et al. Identification of novel sRNAs involved in biofilm formation, motility, and fimbriae formation in Escherichia coli. Sci Rep. 2015;5: 15287.](http://paperpile.com/b/Be81W5/X7221)

4. [Demerec M, Fano U. Bacteriophage-Resistant Mutants in Escherichia Coli. Genetics. 1945;30: 119–136.](http://paperpile.com/b/Be81W5/CNLPc)

5. [Bradley DE. Ultrastructure of bacteriophage and bacteriocins. Bacteriological Reviews. 1967. pp. 230–314. doi:](http://paperpile.com/b/Be81W5/NWySM)[10.1128/mmbr.31.4.230-314.1967](http://dx.doi.org/10.1128/mmbr.31.4.230-314.1967)

6. [Davison J. Pre-early functions of bacteriophage T5 and its relatives. Bacteriophage. 2015;5: e1086500.](http://paperpile.com/b/Be81W5/zwlTS)

7. [Abedon ST. The murky origin of Snow White and her T-even dwarfs. Genetics. 2000;155: 481–486.](http://paperpile.com/b/Be81W5/TiQj4)

8. [Hantke K. Major outer membrane proteins of E. coli K12 serve as receptors for the phages T2 (protein Ia) and 434 (protein Ib). Mol Gen Genet. 1978;164: 131–135.](http://paperpile.com/b/Be81W5/VC5rt)

9. [Lenski RE. Two-step resistance by Escherichia coli B to bacteriophage T2. Genetics. 1984;107: 1–7.](http://paperpile.com/b/Be81W5/tENak)

10. [Morona R, Henning U. New locus (ttr) in Escherichia coli K-12 affecting sensitivity to bacteriophage T2 and growth on oleate as the sole carbon source. J Bacteriol. 1986;168: 534–540.](http://paperpile.com/b/Be81W5/WT26h)

11. [Black PN. The fadL gene product of Escherichia coli is an outer membrane protein required for uptake of long-chain fatty acids and involved in sensitivity to bacteriophage T2. J Bacteriol. 1988;170: 2850–2854.](http://paperpile.com/b/Be81W5/BZxLI)

12. [Kortright KE, Chan BK, Turner PE. High-throughput discovery of phage receptors using transposon insertion sequencing of bacteria. Proceedings of the National Academy of Sciences. 2020. p. 202001888. doi:](http://paperpile.com/b/Be81W5/XqEX)[10.1073/pnas.2001888117](http://dx.doi.org/10.1073/pnas.2001888117)

13. [Wall E, Majdalani N, Gottesman S. The Complex Rcs Regulatory Cascade. Annu Rev Microbiol. 2018;72: 111–139.](http://paperpile.com/b/Be81W5/WvDsP)

14. [Baba T, Ara T, Hasegawa M, Takai Y, Okumura Y, Baba M, et al. Construction of Escherichia coli K-12 in-frame, single-gene knockout mutants: the Keio collection. Mol Syst Biol. 2006;2: 2006.0008.](http://paperpile.com/b/Be81W5/1XB9k)

15. [Allen MD, Christie M, Jones P, Porebski BT, Roome B, Freund SMV, et al. Solution structure of a soluble fragment derived from a membrane protein by shotgun proteolysis. Protein Eng Des Sel. 2015;28: 445–450.](http://paperpile.com/b/Be81W5/ikeSa)

16. [Brooke JS, Valvano MA. Biosynthesis of inner core lipopolysaccharide in enteric bacteria identification and characterization of a conserved phosphoheptose isomerase. J Biol Chem. 1996;271: 3608–3614.](http://paperpile.com/b/Be81W5/VrIv4)

17. [Karow M, Raina S, Georgopoulos C, Fayet O. Complex phenotypes of null mutations in the htr genes, whose products are essential for Escherichia coli growth at elevated temperatures. Res Microbiol. 1991;142: 289–294.](http://paperpile.com/b/Be81W5/zjtKi)

18. [Beher MG, Schnaitman CA. Regulation of the OmpA outer membrane protein of Escherichia coli. J Bacteriol. 1981;147: 972–985.](http://paperpile.com/b/Be81W5/trHhW)

19. [Frirdich E, Whitfield C. Lipopolysaccharide inner core oligosaccharide structure and outer membrane stability in human pathogens belonging to the Enterobacteriaceae. J Endotoxin Res. 2005;11: 133–144.](http://paperpile.com/b/Be81W5/UaDKv)

20. [Pagnout C, Sohm B, Razafitianamaharavo A, Caillet C, Offroy M, Leduc M, et al. Pleiotropic effects of rfa-gene mutations on Escherichia coli envelope properties. Sci Rep. 2019;9: 9696.](http://paperpile.com/b/Be81W5/EwXfl)

21. [Yethon JA, Vinogradov E, Perry MB, Whitfield C. Mutation of the lipopolysaccharide core glycosyltransferase encoded by waaG destabilizes the outer membrane of Escherichia coli by interfering with core phosphorylation. J Bacteriol. 2000;182: 5620–5623.](http://paperpile.com/b/Be81W5/O7gxo)

22. [Klein G, Lindner B, Brabetz W, Brade H, Raina S. Escherichia coli K-12 Suppressor-free Mutants Lacking Early Glycosyltransferases and Late Acyltransferases: minimal lipopolysaccharide structure and induction of envelope stress response. J Biol Chem. 2009;284: 15369–15389.](http://paperpile.com/b/Be81W5/7exEh)

23. [Ahmed N. Faculty of 1000 evaluation for Evidence for an autonomous 5’ target recognition domain in an Hfq-associated small RNA. F1000 - Post-publication peer review of the biomedical literature. 2011. doi:](http://paperpile.com/b/Be81W5/r4elt)[10.3410/f.6951956.7741055](http://dx.doi.org/10.3410/f.6951956.7741055)

24. [Bertani B, Ruiz N. Function and Biogenesis of Lipopolysaccharides. EcoSal Plus. 2018;8. doi:](http://paperpile.com/b/Be81W5/JmSYX)[10.1128/ecosalplus.ESP-0001-2018](http://dx.doi.org/10.1128/ecosalplus.ESP-0001-2018)

25. [Mitchell AM, Silhavy TJ. Envelope stress responses: balancing damage repair and toxicity. Nat Rev Microbiol. 2019;17: 417–428.](http://paperpile.com/b/Be81W5/bR2te)

26. [Lima S, Guo MS, Chaba R, Gross CA, Sauer RT. Dual molecular signals mediate the bacterial response to outer-membrane stress. Science. 2013;340: 837–841.](http://paperpile.com/b/Be81W5/ajpGU)

27. [Klein G, Stupak A, Biernacka D, Wojtkiewicz P, Lindner B, Raina S. Multiple Transcriptional Factors Regulate Transcription of the rpoE Gene in Escherichia coli under Different Growth Conditions and When the Lipopolysaccharide Biosynthesis Is Defective. J Biol Chem. 2016;291: 22999–23019.](http://paperpile.com/b/Be81W5/eXB6b)

28. [Klein G, Raina S. Small regulatory bacterial RNAs regulating the envelope stress response. Biochem Soc Trans. 2017;45: 417–425.](http://paperpile.com/b/Be81W5/lsVZX)

29. [Klein G, Raina S. Regulated Assembly of LPS, Its Structural Alterations and Cellular Response to LPS Defects. Int J Mol Sci. 2019;20. doi:](http://paperpile.com/b/Be81W5/bCtLU)[10.3390/ijms20020356](http://dx.doi.org/10.3390/ijms20020356)

30. [Washizaki A, Yonesaki T, Otsuka Y. Characterization of the interactions between Escherichia coli receptors, LPS and OmpC, and bacteriophage T4 long tail fibers. Microbiologyopen. 2016;5: 1003–1015.](http://paperpile.com/b/Be81W5/att6I)

31. [Shibuya I. Metabolic regulations and biological functions of phospholipids in Escherichia coli. Prog Lipid Res. 1992;31: 245–299.](http://paperpile.com/b/Be81W5/RfZKj)

32. [Dalebroux ZD, Edrozo MB, Pfuetzner RA, Ressl S, Kulasekara BR, Blanc M-P, et al. Delivery of cardiolipins to the Salmonella outer membrane is necessary for survival within host tissues and virulence. Cell Host Microbe. 2015;17: 441–451.](http://paperpile.com/b/Be81W5/gPso1)

33. [Nepper JF, Lin YC, Weibel DB. Rcs phosphorelay activation in cardiolipin-deficient Escherichia coli reduces biofilm formation. bioRxiv. 2019. p. 522219. doi:](http://paperpile.com/b/Be81W5/61Sul)[10.1101/522219](http://dx.doi.org/10.1101/522219)

34. [Shibuya I. Metabolic regulations and biological functions of phospholipids in Escherichia coli. Prog Lipid Res. 1992;31: 245–299.](http://paperpile.com/b/Be81W5/bbMaL)

35. [Dalebroux ZD, Edrozo MB, Pfuetzner RA, Ressl S, Kulasekara BR, Blanc M-P, et al. Delivery of cardiolipins to the Salmonella outer membrane is necessary for survival within host tissues and virulence. Cell Host Microbe. 2015;17: 441–451.](http://paperpile.com/b/Be81W5/Tf0VK)

36. [Cowe E, Sharp PM. Molecular evolution of bacteriophages: Discrete patterns of codon usage in T4 genes are related to the time of gene expression. Journal of Molecular Evolution. 1991. pp. 13–22. doi:](http://paperpile.com/b/Be81W5/xUECw)[10.1007/bf02100191](http://dx.doi.org/10.1007/bf02100191)

37. [Albers S, Czech A. Exploiting tRNAs to Boost Virulence. Life. 2016;6. doi:](http://paperpile.com/b/Be81W5/qe6oe)[10.3390/life6010004](http://dx.doi.org/10.3390/life6010004)

38. [Littauer UZ, Daniel V. The induction of tRNA synthesis following T4 phage infection. Journal of Cellular Physiology. 1969. pp. 71–80. doi:](http://paperpile.com/b/Be81W5/fCjXn)[10.1002/jcp.1040740406](http://dx.doi.org/10.1002/jcp.1040740406)

39. [Neidhardt FC, Marchin GL, McClain WH, Boyd RF, Earhart CF. Phage-Induced modification of valyl-tRNA synthetase. Journal of Cellular Physiology. 1969. pp. 87–101. doi:](http://paperpile.com/b/Be81W5/ck6BE)[10.1002/jcp.1040740408](http://dx.doi.org/10.1002/jcp.1040740408)

40. [Stout V, Torres-Cabassa A, Maurizi MR, Gutnick D, Gottesman S. RcsA, an unstable positive regulator of capsular polysaccharide synthesis. J Bacteriol. 1991;173: 1738–1747.](http://paperpile.com/b/Be81W5/bQxjA)

41. [Ebel W, Trempy JE. Escherichia coli RcsA, a positive activator of colanic acid capsular polysaccharide synthesis, functions To activate its own expression. J Bacteriol. 1999;181: 577–584.](http://paperpile.com/b/Be81W5/mb8vP)

42. [Qimron U, Marintcheva B, Tabor S, Richardson CC. Genomewide screens for Escherichia coli genes affecting growth of T7 bacteriophage. Proceedings of the National Academy of Sciences. 2006. pp. 19039–19044. doi:](http://paperpile.com/b/Be81W5/4iv6S)[10.1073/pnas.0609428103](http://dx.doi.org/10.1073/pnas.0609428103)

43. [Hancock RE, Reeves P. Bacteriophage resistance in Escherichia coli K-12: general pattern of resistance. J Bacteriol. 1975;121: 983–993.](http://paperpile.com/b/Be81W5/pVSzy)

44. [Chaudhry W, Lee E, Worthy A, Weiss Z, Grabowicz M, Vega N, et al. Mucoidy, a general mechanism for maintaining lytic phage in populations of bacteria. doi:](http://paperpile.com/b/Be81W5/CtXpi)[10.1101/775056](http://dx.doi.org/10.1101/775056)

45. [Ross TK, Achberger EC, Braymer HD. Identification of a second polypeptide required for McrB restriction of 5-methylcytosine-containing DNA in Escherichia coli K12. Mol Gen Genet. 1989;216: 402–407.](http://paperpile.com/b/Be81W5/iRuyP)

46. [Douchin V, Bohn C, Bouloc P. Down-regulation of porins by a small RNA bypasses the essentiality of the regulated intramembrane proteolysis protease RseP in Escherichia coli. J Biol Chem. 2006;281: 12253–12259.](http://paperpile.com/b/Be81W5/zS8Oe)

47. [Baker CS, Morozov I, Suzuki K, Romeo T, Babitzke P. CsrA regulates glycogen biosynthesis by preventing translation of glgC in Escherichia coli. Mol Microbiol. 2002;44: 1599–1610.](http://paperpile.com/b/Be81W5/N9PF3)

48. [Lindberg AA. Bacteriophage receptors. Annu Rev Microbiol. 1973;27: 205–241.](http://paperpile.com/b/Be81W5/Y71H5)

49. [Wright A, McConnell M, Kanegasaki S. Lipopolysaccharide as a Bacteriophage Receptor. Virus Receptors. 1980. pp. 27–57. doi:](http://paperpile.com/b/Be81W5/XYLmz)[10.1007/978-94-011-6918-9_3](http://dx.doi.org/10.1007/978-94-011-6918-9_3)

50. [Plumbridge J, Vimr E. Convergent pathways for utilization of the amino sugars N-acetylglucosamine, N-acetylmannosamine, and N-acetylneuraminic acid by Escherichia coli. J Bacteriol. 1999;181: 47–54.](http://paperpile.com/b/Be81W5/Mzc5Q)

51. [Plumbridge J. Control of the expression of the manXYZ operon in Escherichia coli: Mlc is a negative regulator of the mannose PTS. Mol Microbiol. 1998;27: 369–380.](http://paperpile.com/b/Be81W5/Af05I)

52. [Jeckelmann J-M, Erni B. Carbohydrate Transport by Group Translocation: The Bacterial Phosphoenolpyruvate: Sugar Phosphotransferase System. Subcell Biochem. 2019;92: 223–274.](http://paperpile.com/b/Be81W5/Ra26Y)

53. [Prehm P, Jann B, Jann K, Schmidt G, Stirm S. On a bacteriophage T3 and T4 receptor region within the cell wall lipopolysaccharide of escherichia coli B. J Mol Biol. 1976;101: 277–281.](http://paperpile.com/b/Be81W5/X47wS)

54. [Ando H, Lemire S, Pires DP, Lu TK. Engineering Modular Viral Scaffolds for Targeted Bacterial Population Editing. Cell Syst. 2015;1: 187–196.](http://paperpile.com/b/Be81W5/PBGXH)

55. [Molineux I. T7 Bacteriophages. Encyclopedia of Molecular Biology. 2002. doi:](http://paperpile.com/b/Be81W5/pGZwS)[10.1002/047120918x.emb1510](http://dx.doi.org/10.1002/047120918x.emb1510)

56. [Perry EB, Barrick JE, Bohannan BJM. The Molecular and Genetic Basis of Repeatable Coevolution between Escherichia coli and Bacteriophage T3 in a Laboratory Microcosm. PLoS One. 2015;10: e0130639.](http://paperpile.com/b/Be81W5/mQjYZ)

57. [Keseler IM, Mackie A, Santos-Zavaleta A, Billington R, Bonavides-Martínez C, Caspi R, et al. The EcoCyc database: reflecting new knowledge aboutEscherichia coliK-12. Nucleic Acids Research. 2017. pp. D543–D550. doi:](http://paperpile.com/b/Be81W5/SY1TE)[10.1093/nar/gkw1003](http://dx.doi.org/10.1093/nar/gkw1003)

58. [Ren G, Wang Z, Li Y, Hu X, Wang X. Effects of Lipopolysaccharide Core Sugar Deficiency on Colanic Acid Biosynthesis in Escherichia coli. J Bacteriol. 2016;198: 1576–1584.](http://paperpile.com/b/Be81W5/O6KRR)

59. [Pradel E, Parker CT, Schnaitman CA. Structures of the rfaB, rfaI, rfaJ, and rfaS genes of Escherichia coli K-12 and their roles in assembly of the lipopolysaccharide core. J Bacteriol. 1992;174: 4736–4745.](http://paperpile.com/b/Be81W5/pjqJk)

60. [Nakao R, Ramstedt M, Wai SN, Uhlin BE. Enhanced biofilm formation by Escherichia coli LPS mutants defective in Hep biosynthesis. PLoS One. 2012;7: e51241.](http://paperpile.com/b/Be81W5/s460O)

61. [Genevaux P, Bauda P, DuBow MS, Oudega B. Identification of Tn 10 insertions in the rfaG , rfaP , and galU genes involved in lipopolysaccharide core biosynthesis that affect Escherichia coli adhesion. Archives of Microbiology. 1999. pp. 1–8. doi:](http://paperpile.com/b/Be81W5/aJewe)[10.1007/s002030050732](http://dx.doi.org/10.1007/s002030050732)

62. [Danese PN, Pratt LA, Kolter R. Exopolysaccharide production is required for development of Escherichia coli K-12 biofilm architecture. J Bacteriol. 2000;182: 3593–3596.](http://paperpile.com/b/Be81W5/NlGbv)

63. [Casjens SR, Hendrix RW. Bacteriophage lambda: Early pioneer and still relevant. Virology. 2015;479-480: 310–330.](http://paperpile.com/b/Be81W5/UfcLn)

64. [Grodberg J, Dunn JJ. ompT encodes the Escherichia coli outer membrane protease that cleaves T7 RNA polymerase during purification. J Bacteriol. 1988;170: 1245–1253.](http://paperpile.com/b/Be81W5/hFGcl)

65. [Torres-Cabassa AS, Gottesman S. Capsule synthesis in Escherichia coli K-12 is regulated by proteolysis. J Bacteriol. 1987;169: 981–989.](http://paperpile.com/b/Be81W5/3oHoP)

66. [Danese PN, Pratt LA, Kolter R. Exopolysaccharide production is required for development of Escherichia coli K-12 biofilm architecture. J Bacteriol. 2000;182: 3593–3596.](http://paperpile.com/b/Be81W5/M9cr0)

67. [Eydallin G, Morán-Zorzano MT, Muñoz FJ, Baroja-Fernández E, Montero M, Alonso-Casajús N, et al. An Escherichia coli mutant producing a truncated inactive form of GlgC synthesizes glycogen: further evidences for the occurrence of various important sources of ADPglucose in enterobacteria. FEBS Lett. 2007;581: 4417–4422.](http://paperpile.com/b/Be81W5/trP2H)

68. [Baker CS, Morozov I, Suzuki K, Romeo T, Babitzke P. CsrA regulates glycogen biosynthesis by preventing translation of glgC in Escherichia coli. Mol Microbiol. 2002;44: 1599–1610.](http://paperpile.com/b/Be81W5/LEFO1)

69. [Romeo T, Gong M, Liu MY, Brun-Zinkernagel AM. Identification and molecular characterization of csrA, a pleiotropic gene from Escherichia coli that affects glycogen biosynthesis, gluconeogenesis, cell size, and surface properties. J Bacteriol. 1993;175: 4744–4755.](http://paperpile.com/b/Be81W5/bCSw7)

70. [Wang X, Dubey AK, Suzuki K, Baker CS, Babitzke P, Romeo T. CsrA post-transcriptionally represses pgaABCD, responsible for synthesis of a biofilm polysaccharide adhesin of Escherichia coli. Mol Microbiol. 2005;56: 1648–1663.](http://paperpile.com/b/Be81W5/ZbwJC)

71. [Jackson DW, Suzuki K, Oakford L, Simecka JW, Hart ME, Romeo T. Biofilm formation and dispersal under the influence of the global regulator CsrA of Escherichia coli. J Bacteriol. 2002;184: 290–301.](http://paperpile.com/b/Be81W5/E2Fso)

72. [Romeo T, Babitzke P. Global Regulation by CsrA and Its RNA Antagonists. Microbiol Spectr. 2018;6. doi:](http://paperpile.com/b/Be81W5/Rlcr2)[10.1128/microbiolspec.RWR-0009-2017](http://dx.doi.org/10.1128/microbiolspec.RWR-0009-2017)

73. [Palaniyandi S, Mitra A, Herren CD, Lockatell CV, Johnson DE, Zhu X, et al. BarA-UvrY two-component system regulates virulence of uropathogenic E. coli CFT073. PLoS One. 2012;7: e31348.](http://paperpile.com/b/Be81W5/3o0BT)

74. [Klein G, Raina S. Regulated Control of the Assembly and Diversity of LPS by Noncoding sRNAs. Biomed Res Int. 2015;2015: 153561.](http://paperpile.com/b/Be81W5/ZQ0JW)

75. [Painbeni E, Caroff M, Rouviere-Yaniv J. Alterations of the outer membrane composition in Escherichia coli lacking the histone-like protein HU. Proc Natl Acad Sci U S A. 1997;94: 6712–6717.](http://paperpile.com/b/Be81W5/lAzs8)

76. [Miller ES, Kutter E, Mosig G, Arisaka F, Kunisawa T, Rüger W. Bacteriophage T4 genome. Microbiol Mol Biol Rev. 2003;67: 86–156, table of contents.](http://paperpile.com/b/Be81W5/WZB9c)

77. [Karam JD, Drake JW. Molecular biology of bacteriophage. American Society for Microbiology; 1994.](http://paperpile.com/b/Be81W5/iR1tV)

78. [Calendar R. The Bacteriophages. Springer Science & Business Media; 2012.](http://paperpile.com/b/Be81W5/DutHj)

79. [Bryan D, El-Shibiny A, Hobbs Z, Porter J, Kutter EM. Bacteriophage T4 Infection of Stationary Phase E. coli: Life after Log from a Phage Perspective. Frontiers in Microbiology. 2016. doi:](http://paperpile.com/b/Be81W5/xFwOI)[10.3389/fmicb.2016.01391](http://dx.doi.org/10.3389/fmicb.2016.01391)

80. [Schwartz M. Interaction of Phages with their Receptor Proteins. Virus Receptors. 1980. pp. 59–94. doi:](http://paperpile.com/b/Be81W5/SchVT)[10.1007/978-94-011-6918-9_4](http://dx.doi.org/10.1007/978-94-011-6918-9_4)

81. [Kutter E, Bryan D, Ray G, Brewster E, Blasdel B, Guttman B. From Host to Phage Metabolism: Hot Tales of Phage T4’s Takeover of. Viruses. 2018;10. doi:](http://paperpile.com/b/Be81W5/AlFT2)[10.3390/v10070387](http://dx.doi.org/10.3390/v10070387)

82. [Yu F, Mizushima S. Roles of lipopolysaccharide and outer membrane protein OmpC of Escherichia coli K-12 in the receptor function for bacteriophage T4. J Bacteriol. 1982;151: 718–722.](http://paperpile.com/b/Be81W5/ZCqOk)

83. [Montag D, Riede I, Eschbach ML, Degen M, Henning U. Receptor-recognizing proteins of T-even type bacteriophages. Constant and hypervariable regions and an unusual case of evolution. J Mol Biol. 1987;196: 165–174.](http://paperpile.com/b/Be81W5/vd82I)

84. [Islam MZ, Fokine A, Mahalingam M, Zhang Z, Garcia-Doval C, van Raaij MJ, et al. Molecular Anatomy of the Receptor Binding Module of a Bacteriophage Long Tail Fiber. PLoS Pathog. 2019;15: e1008193.](http://paperpile.com/b/Be81W5/a86UE)

85. [Furukawa H, Yamada H, Mizushima S. Interaction of bacteriophage T4 with reconstituted cell envelopes of Escherichia coli K-12. J Bacteriol. 1979;140: 1071–1080.](http://paperpile.com/b/Be81W5/poN19)

86. [Prehm P, Schmidt G, Jann B, Jann K. The cell-wall lipopolysaccharide of Escherichia coli K-12. Structure and acceptor site for O-antigen and other substituents. Eur J Biochem. 1976;70: 171–177.](http://paperpile.com/b/Be81W5/G3M2m)

87. [Montag D, Hashemolhosseini S, Henning U. Receptor-recognizing proteins of T-even type bacteriophages. The receptor-recognizing area of proteins 37 of phages T4 TuIa and TuIb. J Mol Biol. 1990;216: 327–334.](http://paperpile.com/b/Be81W5/KVoC4)

88. [Hattman S, Fukasawa T. HOST-INDUCED MODIFICATION OF T-EVEN PHAGES DUE TO DEFECTIVE GLUCOSYLATION OF THEIR DNA. Proc Natl Acad Sci U S A. 1963;50: 297–300.](http://paperpile.com/b/Be81W5/bNiK8)

89. [Adams MH. Bacteriophages. With chapters by E. S. Anderson [and others] Electron micrographs by E. Kellenberger. 1959. doi:](http://paperpile.com/b/Be81W5/vtTQV)[10.5962/bhl.title.6966](http://dx.doi.org/10.5962/bhl.title.6966)

90. [Murata A, Odaka M, Mukuno S. The Bacteriophage-Inactivating Effect of Basic Amino Acids; Arginine, Histidine, and Lysine. Agricultural and Biological Chemistry. 1974. pp. 477–478. doi:](http://paperpile.com/b/Be81W5/60HN2)[10.1080/00021369.1974.10861180](http://dx.doi.org/10.1080/00021369.1974.10861180)

91. [Kim MS, Kim YD, Hong SS, Park K, Ko KS, Myung H. Phage-encoded colanic acid-degrading enzyme permits lytic phage infection of a capsule-forming resistant mutant Escherichia coli strain. Appl Environ Microbiol. 2015;81: 900–909.](http://paperpile.com/b/Be81W5/vSxPk)

92. [Rousset F, Cui L, Siouve E, Becavin C, Depardieu F, Bikard D. Genome-wide CRISPR-dCas9 screens in E. coli identify essential genes and phage host factors. PLoS Genet. 2018;14: e1007749.](http://paperpile.com/b/Be81W5/t3Lxq)

93. [Cowley LA, Low AS, Pickard D, Boinett CJ, Dallman TJ, Day M, et al. Transposon Insertion Sequencing Elucidates Novel Gene Involvement in Susceptibility and Resistance to Phages T4 and T7 inEscherichia coliO157. mBio. 2018. doi:](http://paperpile.com/b/Be81W5/Wu2mh)[10.1128/mbio.00705-18](http://dx.doi.org/10.1128/mbio.00705-18)

94. [Tamaki S, Sato T, Matsuhashi M. Role of lipopolysaccharides in antibiotic resistance and bacteriophage adsorption of Escherichia coli K-12. J Bacteriol. 1971;105: 968–975.](http://paperpile.com/b/Be81W5/gdynZ)

95. [Weidel W. Bacterial viruses; with particular reference to adsorption/penetration. Annu Rev Microbiol. 1958;12: 27–48.](http://paperpile.com/b/Be81W5/xqjem)

96. [Yan A, Guan Z, Raetz CRH. An undecaprenyl phosphate-aminoarabinose flippase required for polymyxin resistance in Escherichia coli. J Biol Chem. 2007;282: 36077–36089.](http://paperpile.com/b/Be81W5/sikSh)

97. [Wang X, Kim Y, Ma Q, Hong SH, Pokusaeva K, Sturino JM, et al. Cryptic prophages help bacteria cope with adverse environments. Nat Commun. 2010;1: 147.](http://paperpile.com/b/Be81W5/Y1Txa)

98. [Vakulskas CA, Pannuri A, Cortés-Selva D, Zere TR, Ahmer BM, Babitzke P, et al. Global effects of the DEAD-box RNA helicase DeaD (CsdA) on gene expression over a broad range of temperatures. Molecular Microbiology. 2014. pp. 945–958. doi:](http://paperpile.com/b/Be81W5/tOaJi)[10.1111/mmi.12606](http://dx.doi.org/10.1111/mmi.12606)

99. [Zere TR, Vakulskas CA, Leng Y, Pannuri A, Potts AH, Dias R, et al. Genomic Targets and Features of BarA-UvrY (-SirA) Signal Transduction Systems. PLoS One. 2015;10: e0145035.](http://paperpile.com/b/Be81W5/la5Y5)

100. [Hantke K, Braun V. Functional interaction of the tonA/tonB receptor system in Escherichia coli. J Bacteriol. 1978;135: 190–197.](http://paperpile.com/b/Be81W5/wAllY)

101. [Coulton JW, Mason P, Cameron DR, Carmel G, Jean R, Rode HN. Protein fusions of beta-galactosidase to the ferrichrome-iron receptor of Escherichia coli K-12. Journal of Bacteriology. 1986. pp. 181–192. doi:](http://paperpile.com/b/Be81W5/YpGfV)[10.1128/jb.165.1.181-192.1986](http://dx.doi.org/10.1128/jb.165.1.181-192.1986)

102. [Heller KJ. Molecular interaction between bacteriophage and the gram-negative cell envelope. Archives of Microbiology. 1992. pp. 235–248. doi:](http://paperpile.com/b/Be81W5/fnwPr)[10.1007/bf00245239](http://dx.doi.org/10.1007/bf00245239)

103. [McHugh JP, Rodríguez-Quinoñes F, Abdul-Tehrani H, Svistunenko DA, Poole RK, Cooper CE, et al. Global iron-dependent gene regulation in Escherichia coli. A new mechanism for iron homeostasis. J Biol Chem. 2003;278: 29478–29486.](http://paperpile.com/b/Be81W5/hTIhc)

104. [Bagg A, Neilands JB. Ferric uptake regulation protein acts as a repressor, employing iron(II) as a cofactor to bind the operator of an iron transport operon in Escherichia coli. Biochemistry. 1987. pp. 5471–5477. doi:](http://paperpile.com/b/Be81W5/TS9sS)[10.1021/bi00391a039](http://dx.doi.org/10.1021/bi00391a039)

105. [Hantke K. Regulation of ferric iron transport in Escherichia coli K12: isolation of a constitutive mutant. Mol Gen Genet. 1981;182: 288–292.](http://paperpile.com/b/Be81W5/lNyD6)

106. [Carpenter BM, Whitmire JM, Merrell DS. This Is Not Your Mother’s Repressor: the Complex Role of Fur in Pathogenesis. Infection and Immunity. 2009. pp. 2590–2601. doi:](http://paperpile.com/b/Be81W5/k8XXk)[10.1128/iai.00116-09](http://dx.doi.org/10.1128/iai.00116-09)

107. [Kammler M, Schön C, Hantke K. Characterization of the ferrous iron uptake system of Escherichia coli. Journal of Bacteriology. 1993. pp. 6212–6219. doi:](http://paperpile.com/b/Be81W5/a5m9w)[10.1128/jb.175.19.6212-6219.1993](http://dx.doi.org/10.1128/jb.175.19.6212-6219.1993)

108. [Lau CKY, Krewulak KD, Vogel HJ. Bacterial ferrous iron transport: the Feo system. FEMS Microbiol Rev. 2016;40: 273–298.](http://paperpile.com/b/Be81W5/anqTv)

109. [Hantke K. Phage T6 - colicin K receptor and nucleoside transport inEscherichia coli. FEBS Letters. 1976. pp. 109–112. doi:](http://paperpile.com/b/Be81W5/0XfX4)[10.1016/0014-5793(76)80737-5](http://dx.doi.org/10.1016/0014-5793(76)80737-5)

110. [Bremer E, Middendorf A, Martinussen J, Valentin-Hansen P. Analysis of the tsx gene, which encodes a nucleoside-specific channel-forming protein (Tsx) in the outer membrane of Escherichia coli. Gene. 1990;96: 59–65.](http://paperpile.com/b/Be81W5/hgdPr)

111. [Cooley W, Sirotkin K, Green R, Synder L. A new gene of Escherichia coli K-12 whose product participates in T4 bacteriophage late gene expression: interaction of lit with the T4-induced polynucleotide 5’-kinase 3'-phosphatase. J Bacteriol. 1979;140: 83–91.](http://paperpile.com/b/Be81W5/shQT3)

112. [Kao C, Snyder L. The lit gene product which blocks bacteriophage T4 late gene expression is a membrane protein encoded by a cryptic DNA element, e14. J Bacteriol. 1988;170: 2056–2062.](http://paperpile.com/b/Be81W5/aGhKW)

113. [Studier FW. Bacteriophage T7. Science. 1972. pp. 367–376. doi:](http://paperpile.com/b/Be81W5/QRfc4)[10.1126/science.176.4033.367](http://dx.doi.org/10.1126/science.176.4033.367)

114. [Krüger DH, Schroeder C. Bacteriophage T3 and bacteriophage T7 virus-host cell interactions. Microbiol Rev. 1981;45: 9–51.](http://paperpile.com/b/Be81W5/bK5yq)

115. [Chamberlin M. Isolation and characterization of prototrophic mutants of Escherichia coli unable to support the intracellular growth of T7. J Virol. 1974;14: 509–516.](http://paperpile.com/b/Be81W5/l1jYW)

116. [Scholl D, Adhya S, Merril C. Escherichia coli K1’s Capsule Is a Barrier to Bacteriophage T7. Applied and Environmental Microbiology. 2005. pp. 4872–4874. doi:](http://paperpile.com/b/Be81W5/FmdM3)[10.1128/aem.71.8.4872-4874.2005](http://dx.doi.org/10.1128/aem.71.8.4872-4874.2005)

117. [Eydallin G, Morán-Zorzano MT, Muñoz FJ, Baroja-Fernández E, Montero M, Alonso-Casajús N, et al. An Escherichia coli mutant producing a truncated inactive form of GlgC synthesizes glycogen: further evidences for the occurrence of various important sources of ADPglucose in enterobacteria. FEBS Lett. 2007;581: 4417–4422.](http://paperpile.com/b/Be81W5/PeGEK)

118. [Kiino DR, Rothman-Denes LB. Bacteriophage N4. The Bacteriophages. 1988. pp. 457–474. doi:](http://paperpile.com/b/Be81W5/sqB9P)[10.1007/978-1-4684-5490-1_7](http://dx.doi.org/10.1007/978-1-4684-5490-1_7)

119. [Schito GC. The genetics and physiology of coliphage N4. Virology. 1973;55: 254–265.](http://paperpile.com/b/Be81W5/9B0Jz)

120. [Kiino DR, Rothman-Denes LB. Genetic analysis of bacteriophage N4 adsorption. J Bacteriol. 1989;171: 4595–4602.](http://paperpile.com/b/Be81W5/jpMbz)

121. [Kiino DR, Singer MS, Rothman-Denes LB. Two overlapping genes encoding membrane proteins required for bacteriophage N4 adsorption. J Bacteriol. 1993;175: 7081–7085.](http://paperpile.com/b/Be81W5/xRfyu)

122. [McPartland J, Rothman-Denes LB. The tail sheath of bacteriophage N4 interacts with the Escherichia coli receptor. J Bacteriol. 2009;191: 525–532.](http://paperpile.com/b/Be81W5/pMYyW)

123. [Kiino DR, Licudine R, Wilt K, Yang DH, Rothman-Denes LB. A cytoplasmic protein, NfrC, is required for bacteriophage N4 adsorption. J Bacteriol. 1993;175: 7074–7080.](http://paperpile.com/b/Be81W5/p7m22)

124. [Hall BG. The rtn gene of Proteus vulgaris is actually from Escherichia coli. Journal of Bacteriology. 1997. pp. 2433–2434. doi:](http://paperpile.com/b/Be81W5/XWZXI)[10.1128/jb.179.7.2433-2434.1997](http://dx.doi.org/10.1128/jb.179.7.2433-2434.1997)

125. [Chae KS, Yoo OJ. Cloning of the lambda resistant genes from Brevibacterium albidum and Proteus vulgaris into Escherichia coli. Biochem Biophys Res Commun. 1986;140: 1101–1105.](http://paperpile.com/b/Be81W5/O4TNu)

126. [Reinders A, Hee C-S, Ozaki S, Mazur A, Boehm A, Schirmer T, et al. Expression and Genetic Activation of Cyclic Di-GMP-Specific Phosphodiesterases in Escherichia coli. J Bacteriol. 2016;198: 448–462.](http://paperpile.com/b/Be81W5/UGNb0)

127. [Park JH, Lee KH, Kim TY, Lee SY. Metabolic engineering of Escherichia coli for the production of L-valine based on transcriptome analysis and in silico gene knockout simulation. Proc Natl Acad Sci U S A. 2007;104: 7797–7802.](http://paperpile.com/b/Be81W5/zIM2K)

128. [Pesavento C, Becker G, Sommerfeldt N, Possling A, Tschowri N, Mehlis A, et al. Inverse regulatory coordination of motility and curli-mediated adhesion in Escherichia coli. Genes Dev. 2008;22: 2434–2446.](http://paperpile.com/b/Be81W5/WTLyw)

129. [Girgis HS, Liu Y, Ryu WS, Tavazoie S. A comprehensive genetic characterization of bacterial motility. PLoS Genet. 2007;3: 1644–1660.](http://paperpile.com/b/Be81W5/n3G1g)

130. [Sarenko O, Klauck G, Wilke FM, Pfiffer V, Richter AM, Herbst S, et al. More than Enzymes That Make or Break Cyclic Di-GMP—Local Signaling in the Interactome of GGDEF/EAL Domain Proteins of Escherichia coli. mBio. 2017. doi:](http://paperpile.com/b/Be81W5/NvIP6)[10.1128/mbio.01639-17](http://dx.doi.org/10.1128/mbio.01639-17)

131. [Sommerfeldt N, Possling A, Becker G, Pesavento C, Tschowri N, Hengge R. Gene expression patterns and differential input into curli fimbriae regulation of all GGDEF/EAL domain proteins in Escherichia coli. Microbiology. 2009;155: 1318–1331.](http://paperpile.com/b/Be81W5/RCtb0)

132. [Ptashne M. A Genetic Switch: Phage Lambda Revisited. CSHL Press; 2004.](http://paperpile.com/b/Be81W5/VYHv4)

133. [Grodzicker T, Arditti RR, Eisen H. Establishment of repression by lambdoid phage in catabolite activator protein and adenylate cyclase mutants of Escherichia coli. Proc Natl Acad Sci U S A. 1972;69: 366–370.](http://paperpile.com/b/Be81W5/DXnDz)

134. [Herman C, Ogura T, Tomoyasu T, Hiraga S, Akiyama Y, Ito K, et al. Cell growth and lambda phage development controlled by the same essential Escherichia coli gene, ftsH/hflB. Proc Natl Acad Sci U S A. 1993;90: 10861–10865.](http://paperpile.com/b/Be81W5/9Gljv)

135. [Parua PK, Mondal A, Parrack P. HflD, an Escherichia coli protein involved in the lambda lysis-lysogeny switch, impairs transcription activation by lambdaCII. Arch Biochem Biophys. 2010;493: 175–183.](http://paperpile.com/b/Be81W5/2bomO)

136. [Liberek K, Georgopoulos C, Zylicz M. Role of the Escherichia coli DnaK and DnaJ heat shock proteins in the initiation of bacteriophage lambda DNA replication. Proceedings of the National Academy of Sciences. 1988. pp. 6632–6636. doi:](http://paperpile.com/b/Be81W5/SLeHX)[10.1073/pnas.85.18.6632](http://dx.doi.org/10.1073/pnas.85.18.6632)

137. [Randall LL. Quantitation of the loss of the bacteriophage lambda receptor protein from the outer membrane of lipopolysaccharide-deficient strains of Escherichia coli. J Bacteriol. 1975;123: 41–46.](http://paperpile.com/b/Be81W5/7uFMd)

138. [Das A. How the phage lambda N gene product suppresses transcription termination: communication of RNA polymerase with regulatory proteins mediated by signals in nascent RNA. J Bacteriol. 1992;174: 6711–6716.](http://paperpile.com/b/Be81W5/Rnbxg)

139. [Lederberg EM, Lederberg J. Genetic Studies of Lysogenicity in Escherichia Coli. Genetics. 1953;38: 51–64.](http://paperpile.com/b/Be81W5/l1efl)

140. [Gottesman ME, Weisberg RA. Little lambda, who made thee? Microbiol Mol Biol Rev. 2004;68: 796–813.](http://paperpile.com/b/Be81W5/dmSbh)

141. [Wang YF, Dutzler R, Rizkallah PJ, Rosenbusch JP, Schirmer T. Channel specificity: structural basis for sugar discrimination and differential flux rates in maltoporin. J Mol Biol. 1997;272: 56–63.](http://paperpile.com/b/Be81W5/hjaHf)

142. [Schwartz M. Phage lambda receptor (lamB protein) in Escherichia coli. Methods Enzymol. 1983;97: 100–112.](http://paperpile.com/b/Be81W5/XWSVO)

143. [de Vries GE, Raymond CK, Ludwig RA. Extension of bacteriophage lambda host range: selection, cloning, and characterization of a constitutive lambda receptor gene. Proc Natl Acad Sci U S A. 1984;81: 6080–6084.](http://paperpile.com/b/Be81W5/QfOyl)

144. [Débarbouillé M, Shuman HA, Silhavy TJ, Schwartz M. Dominant constitutive mutations in malT, the positive regulator gene of the maltose regulon in Escherichia coli. J Mol Biol. 1978;124: 359–371.](http://paperpile.com/b/Be81W5/RcS01)

145. [Reidl J, Boos W. The malX malY operon of Escherichia coli encodes a novel enzyme II of the phosphotransferase system recognizing glucose and maltose and an enzyme abolishing the endogenous induction of the maltose system. J Bacteriol. 1991;173: 4862–4876.](http://paperpile.com/b/Be81W5/zS5Vr)

146. [Yokota T, Kasuga T. Requirement of adenosine 3’,5'-cyclic phosphate for formation of the phage lambda receptor in Escherichia coli. J Bacteriol. 1972;109: 1304–1306.](http://paperpile.com/b/Be81W5/llYmK)

147. [Pearson ML. The role of adenosine 3′,5′-cyclic monophosphate in the growth of bacteriophage lambda. Virology. 1972. pp. 605–609. doi:](http://paperpile.com/b/Be81W5/SOWlZ)[10.1016/0042-6822(72)90513-2](http://dx.doi.org/10.1016/0042-6822(72)90513-2)

148. [Blasche S, Wuchty S, Rajagopala SV, Uetz P. The protein interaction network of bacteriophage lambda with its host, Escherichia coli. J Virol. 2013;87: 12745–12755.](http://paperpile.com/b/Be81W5/50A2B)

149. [Maynard ND, Birch EW, Sanghvi JC, Chen L, Gutschow MV, Covert MW. A forward-genetic screen and dynamic analysis of lambda phage host-dependencies reveals an extensive interaction network and a new anti-viral strategy. PLoS Genet. 2010;6: e1001017.](http://paperpile.com/b/Be81W5/53tT0)

150. [Ragunathan PT, Vanderpool CK. Cryptic-Prophage-Encoded Small Protein DicB Protects Escherichia coli from Phage Infection by Inhibiting Inner Membrane Receptor Proteins. Journal of Bacteriology. 2019. doi:](http://paperpile.com/b/Be81W5/Mkx2c)[10.1128/jb.00475-19](http://dx.doi.org/10.1128/jb.00475-19)

151. [Yamada H, Nogami T, Mizushima S. Arrangement of bacteriophage lambda receptor protein (LamB) in the cell surface of Escherichia coli: a reconstitution study. J Bacteriol. 1981;147: 660–669.](http://paperpile.com/b/Be81W5/emvSP)

152. [Yamada H, Nogami T, Mizushima S. Arrangement of bacteriophage lambda receptor protein (LamB) in the Escherichia coli cell surface. Ann Microbiol . 1982;133A: 43–47.](http://paperpile.com/b/Be81W5/xJpst)

153. [Hong JS, Smith GR, Ames BN. Adenosine 3’:5'-cyclic monophosphate concentration in the bacterial host regulates the viral decision between lysogeny and lysis. Proc Natl Acad Sci U S A. 1971;68: 2258–2262.](http://paperpile.com/b/Be81W5/OLfy7)

154. [Kimata K, Tanaka Y, Inada T, Aiba H. Expression of the glucose transporter gene, ptsG, is regulated at the mRNA degradation step in response to glycolytic flux in Escherichia coli. EMBO J. 2001;20: 3587–3595.](http://paperpile.com/b/Be81W5/CZbEr)

155. [Cumby N, Reimer K, Mengin-Lecreulx D, Davidson AR, Maxwell KL. The phage tail tape measure protein, an inner membrane protein and a periplasmic chaperone play connected roles in the genome injection process ofE. coliphage HK97. Molecular Microbiology. 2015. pp. 437–447. doi:](http://paperpile.com/b/Be81W5/MpYAH)[10.1111/mmi.12918](http://dx.doi.org/10.1111/mmi.12918)

156. [Henthorn KS. Multilevel regulation of gene expression in lambda and related bacteriophages. 1994.](http://paperpile.com/b/Be81W5/RDYvv)

157. [Zhou Y, Filter JJ, Court DL, Gottesman ME, Friedman DI. Requirement for NusG for transcription antitermination in vivo by the lambda N protein. J Bacteriol. 2002;184: 3416–3418.](http://paperpile.com/b/Be81W5/bqCE9)

158. [Sullivan SL, Ward DF, Gottesman ME. Effect of Escherichia coli nusG function on lambda N-mediated transcription antitermination. Journal of Bacteriology. 1992. pp. 1339–1344. doi:](http://paperpile.com/b/Be81W5/7t17V)[10.1128/jb.174.4.1339-1344.1992](http://dx.doi.org/10.1128/jb.174.4.1339-1344.1992)

159. [Crane JM, Randall LL. The Sec System: Protein Export in Escherichia coli. EcoSal Plus. 2017. doi:](http://paperpile.com/b/Be81W5/xNEoH)[10.1128/ecosalplus.esp-0002-2017](http://dx.doi.org/10.1128/ecosalplus.esp-0002-2017)

160. [Katz C, Ron EZ. Dual role of FtsH in regulating lipopolysaccharide biosynthesis in Escherichia coli. J Bacteriol. 2008;190: 7117–7122.](http://paperpile.com/b/Be81W5/XQGwZ)

161. [Potts AH, Vakulskas CA, Pannuri A, Yakhnin H, Babitzke P, Romeo T. Global role of the bacterial post-transcriptional regulator CsrA revealed by integrated transcriptomics. Nature Communications. 2017. doi:](http://paperpile.com/b/Be81W5/83PNw)[10.1038/s41467-017-01613-1](http://dx.doi.org/10.1038/s41467-017-01613-1)

162. [Schakermann M, Langklotz S, Narberhaus F. FtsH-Mediated Coordination of Lipopolysaccharide Biosynthesis in Escherichia coli Correlates with the Growth Rate and the Alarmone (p)ppGpp. Journal of Bacteriology. 2013. pp. 1912–1919. doi:](http://paperpile.com/b/Be81W5/6muC0)[10.1128/jb.02134-12](http://dx.doi.org/10.1128/jb.02134-12)

163. [Decker K, Plumbridge J, Boos W. Negative transcriptional regulation of a positive regulator: the expression of malT, encoding the transcriptional activator of the maltose regulon of Escherichia coli, is negatively controlled by Mlc. Mol Microbiol. 1998;27: 381–390.](http://paperpile.com/b/Be81W5/duVND)

164. [Lee SJ, Boos W, Bouché JP, Plumbridge J. Signal transduction between a membrane-bound transporter, PtsG, and a soluble transcription factor, Mlc, of Escherichia coli. EMBO J. 2000;19: 5353–5361.](http://paperpile.com/b/Be81W5/0YJIS)

165. [Joly N, Danot O, Schlegel A, Boos W, Richet E. The Aes protein directly controls the activity of MalT, the central transcriptional activator of the Escherichia coli maltose regulon. J Biol Chem. 2002;277: 16606–16613.](http://paperpile.com/b/Be81W5/Yrqp4)

166. [Schlegel A, Danot O, Richet E, Ferenci T, Boos W. The N Terminus of the Escherichia coli Transcription Activator MalT Is the Domain of Interaction with MalY. Journal of Bacteriology. 2002. pp. 3069–3077. doi:](http://paperpile.com/b/Be81W5/rhZ52)[10.1128/jb.184.11.3069-3077.2002](http://dx.doi.org/10.1128/jb.184.11.3069-3077.2002)

167. [Lengsfeld C, Schönert S, Dippel R, Boos W. Glucose- and glucokinase-controlled mal gene expression in Escherichia coli. J Bacteriol. 2009;191: 701–712.](http://paperpile.com/b/Be81W5/utMDp)

168. [Johansen J, Rasmussen AA, Overgaard M, Valentin-Hansen P. Conserved small non-coding RNAs that belong to the sigmaE regulon: role in down-regulation of outer membrane proteins. J Mol Biol. 2006;364: 1–8.](http://paperpile.com/b/Be81W5/aYSmP)

169. [Yarmolinsky MB, Sternberg N. Bacteriophage P1. In: Calendar R, editor. The Bacteriophages. Boston, MA: Springer US; 1988. pp. 291–438.](http://paperpile.com/b/Be81W5/ezQ4q)

170. [Yarmolinsky MB, Sternberg N. Bacteriophage P1. The Bacteriophages. 1988. pp. 291–438. doi:](http://paperpile.com/b/Be81W5/WBTTD)[10.1007/978-1-4684-5424-6_9](http://dx.doi.org/10.1007/978-1-4684-5424-6_9)

171. [Kutter E, Sulakvelidze A. Bacteriophages: Biology and Applications. CRC Press; 2004.](http://paperpile.com/b/Be81W5/8NU36)

172. [Lobocka MB, Rose DJ, Plunkett G, Rusin M, Samojedny A, Lehnherr H, et al. Genome of Bacteriophage P1. Journal of Bacteriology. 2004. pp. 7032–7068. doi:](http://paperpile.com/b/Be81W5/2iboa)[10.1128/jb.186.21.7032-7068.2004](http://dx.doi.org/10.1128/jb.186.21.7032-7068.2004)

173. [Yarmolinsky MB. Bacteriophage P1 in retrospect and in prospect. Journal of bacteriology. 2004. pp. 7025–7028.](http://paperpile.com/b/Be81W5/YgQH0)

174. [Sternberg N. The P1 cloning system: past and future. Mammalian Genome. 1994. pp. 397–404. doi:](http://paperpile.com/b/Be81W5/0zxzi)[10.1007/bf00356999](http://dx.doi.org/10.1007/bf00356999)

175. [Lehnherr H. P1-like Viruses‡. The Springer Index of Viruses. 2011. pp. 915–920. doi:](http://paperpile.com/b/Be81W5/3UMki)[10.1007/978-0-387-95919-1_141](http://dx.doi.org/10.1007/978-0-387-95919-1_141)

176. [Franklin NC. Mutation in gal U gene of E. coli blocks phage P1 infection. Virology. 1969. pp. 189–191. doi:](http://paperpile.com/b/Be81W5/DA68H)[10.1016/0042-6822(69)90144-5](http://dx.doi.org/10.1016/0042-6822(69)90144-5)

177. [Sandulache R, Prehm P, Kamp D. Cell wall receptor for bacteriophage Mu G(+). J Bacteriol. 1984;160: 299–303.](http://paperpile.com/b/Be81W5/JimCz)

178. [Sandulache R, Prehm P, Expert D, Toussaint A, Kamp D. The cell wall receptor for bacteriophage Mu G(â) inErwiniaandEscherichia coliC. FEMS Microbiology Letters. 1985. pp. 307–310. doi:](http://paperpile.com/b/Be81W5/xOmFP)[10.1111/j.1574-6968.1985.tb00811.x](http://dx.doi.org/10.1111/j.1574-6968.1985.tb00811.x)

179. [Ornellas EP, Stocker BA. Relation of lipopolysaccharide character to P1 sensitivity in Salmonella typhimurium. Virology. 1974;60: 491–502.](http://paperpile.com/b/Be81W5/QAs2O)

180. [Ho TD, Waldor MK. Enterohemorrhagic Escherichia coli O157:H7 gal mutants are sensitive to bacteriophage P1 and defective in intestinal colonization. Infect Immun. 2007;75: 1661–1666.](http://paperpile.com/b/Be81W5/Zseza)

181. [Nikaido H. Molecular basis of bacterial outer membrane permeability revisited. Microbiol Mol Biol Rev. 2003;67: 593–656.](http://paperpile.com/b/Be81W5/pQEBo)

182. [Meredith TC, Mamat U, Kaczynski Z, Lindner B, Holst O, Woodard RW. Modification of lipopolysaccharide with colanic acid (M-antigen) repeats in Escherichia coli. J Biol Chem. 2007;282: 7790–7798.](http://paperpile.com/b/Be81W5/pC3mj)

183. [Studier FW, Daegelen P, Lenski RE, Maslov S, Kim JF. Understanding the differences between genome sequences of Escherichia coli B strains REL606 and BL21(DE3) and comparison of the E. coli B and K-12 genomes. J Mol Biol. 2009;394: 653–680.](http://paperpile.com/b/Be81W5/gPYXx)

184. [Palaniyandi S, Mitra A, Herren CD, Lockatell CV, Johnson DE, Zhu X, et al. BarA-UvrY two-component system regulates virulence of uropathogenic E. coli CFT073. PLoS One. 2012;7: e31348.](http://paperpile.com/b/Be81W5/bCYQK)

185. [Sode K, Sugimoto S, Watanabe M, Tsugawa W. Effect of PQQ glucose dehydrogenase overexpression in Escherichia coli on sugar-dependent respiration. J Biotechnol. 1995;43: 41–44.](http://paperpile.com/b/Be81W5/15YwA)

186. [Jackson DW, Suzuki K, Oakford L, Simecka JW, Hart ME, Romeo T. Biofilm formation and dispersal under the influence of the global regulator CsrA of Escherichia coli. J Bacteriol. 2002;184: 290–301.](http://paperpile.com/b/Be81W5/4nadk)

187. [Klein G, Raina S. Regulated Control of the Assembly and Diversity of LPS by Noncoding sRNAs. Biomed Res Int. 2015;2015: 153561.](http://paperpile.com/b/Be81W5/50Taw)

188. [Romeo T, Gong M, Liu MY, Brun-Zinkernagel AM. Identification and molecular characterization of csrA, a pleiotropic gene from Escherichia coli that affects glycogen biosynthesis, gluconeogenesis, cell size, and surface properties. J Bacteriol. 1993;175: 4744–4755.](http://paperpile.com/b/Be81W5/3s1K1)

189. [Wang X, Dubey AK, Suzuki K, Baker CS, Babitzke P, Romeo T. CsrA post-transcriptionally represses pgaABCD, responsible for synthesis of a biofilm polysaccharide adhesin of Escherichia coli. Mol Microbiol. 2005;56: 1648–1663.](http://paperpile.com/b/Be81W5/pSdx2)

190. [Bertani G. STUDIES ON LYSOGENESIS I. Journal of Bacteriology. 1951. pp. 293–300. doi:](http://paperpile.com/b/Be81W5/KiErV)[10.1128/jb.62.3.293-300.1951](http://dx.doi.org/10.1128/jb.62.3.293-300.1951)

191. [Nilsson AS, Karlsson JL, Haggård-Ljungquist E. Site-specific recombination links the evolution of P2-like coliphages and pathogenic enterobacteria. Mol Biol Evol. 2004;21: 1–13.](http://paperpile.com/b/Be81W5/BhNi7)

192. [Nilsson H, Cardoso-Palacios C, Haggård-Ljungquist E, Nilsson AS. Phylogenetic structure and evolution of regulatory genes and integrases of P2-like phages. Bacteriophage. 2011;1: 207–218.](http://paperpile.com/b/Be81W5/l92cq)

193. [Christie GE, Calendar R. Bacteriophage P2. Bacteriophage. 2016. p. e1145782. doi:](http://paperpile.com/b/Be81W5/76Xhx)[10.1080/21597081.2016.1145782](http://dx.doi.org/10.1080/21597081.2016.1145782)

194. [Bertani LE, Elizabeth Bertani L, Six EW. The P2-like Phages and Their Parasite, P4. The Bacteriophages. 1988. pp. 73–143. doi:](http://paperpile.com/b/Be81W5/3Ee2w)[10.1007/978-1-4684-5490-1_2](http://dx.doi.org/10.1007/978-1-4684-5490-1_2)

195. [Grundy FJ. Involvement of the invertible G segment genes in bacteriophage Mu tail fiber biosynthesis. 1984.](http://paperpile.com/b/Be81W5/14ERa)

196. [Haggård-Ljungquist E, Halling C, Calendar R. DNA sequences of the tail fiber genes of bacteriophage P2: evidence for horizontal transfer of tail fiber genes among unrelated bacteriophages. J Bacteriol. 1992;174: 1462–1477.](http://paperpile.com/b/Be81W5/AQ22x)

197. [Bertani LE, Six EW. The P2-like phages and their parasite, P4. The bacteriophages. 1988;2: 73–143.](http://paperpile.com/b/Be81W5/VAKdo)

198. [Bertani LE, Elizabeth Bertani L, Bertani G. Genetics of P2 and Related Phages. Advances in Genetics. 1971. pp. 199–237. doi:](http://paperpile.com/b/Be81W5/gB8B3)[10.1016/s0065-2660(08)60359-4](http://dx.doi.org/10.1016/s0065-2660(08)60359-4)

199. [Christie GE, Calendar RL. P2-like viruses. The Springer Index of Viruses. pp. 559–564. doi:](http://paperpile.com/b/Be81W5/YlX6j)[10.1007/3-540-31042-8_93](http://dx.doi.org/10.1007/3-540-31042-8_93)

200. [Xue Q, Egan JB. DNA sequence of tail fiber genes of coliphage 186 and evidence for a common ancestor shared by dsDNA phage fiber genes. Virology. 1995;212: 128–133.](http://paperpile.com/b/Be81W5/7o4uq)

201. [Woods WH, Egan JB. Prophage induction of noninducible coliphage 186. J Virol. 1974;14: 1349–1356.](http://paperpile.com/b/Be81W5/ImMyQ)

202. [Pradel E, Schnaitman CA. Effect of rfaH (sfrB) and temperature on expression of rfa genes of Escherichia coli K-12. J Bacteriol. 1991;173: 6428–6431.](http://paperpile.com/b/Be81W5/twUnU)

203. [Creeger ES, Schulte T, Rothfield LI. Regulation of membrane glycosyltransferases by the sfrB and rfaH genes of Escherichia coli and Salmonella typhimurium. J Biol Chem. 1984;259: 3064–3069.](http://paperpile.com/b/Be81W5/fQAUa)

204. [Nikaido H. Biosynthesis of Cell Wall Lipopolysaccharide in Gram-Negative Enteric Bacteria. Advances in Enzymology - and Related Areas of Molecular Biology. 2006. pp. 77–124. doi:](http://paperpile.com/b/Be81W5/HUQ7J)[10.1002/9780470122761.ch3](http://dx.doi.org/10.1002/9780470122761.ch3)

205. [Schnaitman CA, Klena JD. Genetics of lipopolysaccharide biosynthesis in enteric bacteria. Microbiol Rev. 1993;57: 655–682.](http://paperpile.com/b/Be81W5/B8J7h)

206. [Polissi A, Goffin L, Georgopoulos C. The Escherichia coli heat shock response and bacteriophage lambda development. FEMS Microbiol Rev. 1995;17: 159–169.](http://paperpile.com/b/Be81W5/l8dAO)

207. [Champ S, Puvirajesinghe TM, Perrody E, Menouni R, Genevaux P, Ansaldi M. Chaperone-assisted excisive recombination, a solitary role for DnaJ (Hsp40) chaperone in lysogeny escape. J Biol Chem. 2011;286: 38876–38885.](http://paperpile.com/b/Be81W5/Lg9EH)

208. [Hoffmann HJ, Lyman SK, Lu C, Petit MA, Echols H. Activity of the Hsp70 chaperone complex--DnaK, DnaJ, and GrpE--in initiating phage lambda DNA replication by sequestering and releasing lambda P protein. Proceedings of the National Academy of Sciences. 1992. pp. 12108–12111. doi:](http://paperpile.com/b/Be81W5/SZZvI)[10.1073/pnas.89.24.12108](http://dx.doi.org/10.1073/pnas.89.24.12108)

209. [Odegrip R, Schoen S, Haggård-Ljungquist E, Park K, Chattoraj DK. The interaction of bacteriophage P2 B protein with Escherichia coli DnaB helicase. J Virol. 2000;74: 4057–4063.](http://paperpile.com/b/Be81W5/1V95F)

210. [Skowyra D, McKenney K, Wickner SH. Function of molecular chaperones in bacteriophage and plasmid DNA replication. Seminars in Virology. 1995. pp. 43–51. doi:](http://paperpile.com/b/Be81W5/8es8W)[10.1016/s1044-5773(05)80008-3](http://dx.doi.org/10.1016/s1044-5773(05)80008-3)

211. [Foster PL, Marinus MG. Levels of epsilon, an essential replication subunit of Escherichia coli DNA polymerase III, are controlled by heat shock proteins. J Bacteriol. 1992;174: 7509–7516.](http://paperpile.com/b/Be81W5/swt39)

212. [Sell SM, Eisen C, Ang D, Zylicz M, Georgopoulos C. Isolation and characterization of dnaJ null mutants of Escherichia coli. Journal of Bacteriology. 1990. pp. 4827–4835. doi:](http://paperpile.com/b/Be81W5/RVPbW)[10.1128/jb.172.9.4827-4835.1990](http://dx.doi.org/10.1128/jb.172.9.4827-4835.1990)

213. [Calendar R, Lindqvist B, Sironi G, Clark AJ. Characterization of REP− mutants and their interaction with P2 phage. Virology. 1970. pp. 72–83. doi:](http://paperpile.com/b/Be81W5/ZrHug)[10.1016/0042-6822(70)90380-6](http://dx.doi.org/10.1016/0042-6822(70)90380-6)

214. [Guarneros G. Retroregulation of Bacteriophage λ int Gene Expression. Current Topics in Microbiology and Immunology. 1988. pp. 1–19. doi:](http://paperpile.com/b/Be81W5/NorSl)[10.1007/978-3-642-73115-0_1](http://dx.doi.org/10.1007/978-3-642-73115-0_1)

215. [Birenbaum M, Schlessinger D, Ohnishi Y. Altered bacteriophage T4 ribonucleic acid metabolism in a ribonuclease II-deficient mutant of Escherichia coli. J Bacteriol. 1980;142: 327–330.](http://paperpile.com/b/Be81W5/lKWb9)

216. [Raya RR, Varey P, Oot RA, Dyen MR, Callaway TR, Edrington TS, et al. Isolation and characterization of a new T-even bacteriophage, CEV1, and determination of its potential to reduce Escherichia coli O157:H7 levels in sheep. Appl Environ Microbiol. 2006;72: 6405–6410.](http://paperpile.com/b/Be81W5/qkUFp)

217. [Montag D, Hashemolhosseini S, Henning U. Receptor-recognizing proteins of T-even type bacteriophages. Journal of Molecular Biology. 1990. pp. 327–334. doi:](http://paperpile.com/b/Be81W5/1xW9P)[10.1016/s0022-2836(05)80324-9](http://dx.doi.org/10.1016/s0022-2836(05)80324-9)

218. [Cohen PS, Ennis HL. The requirement for potassium for bacteriophage T4 protein and deoxyribonucleic acid synthesis. Virology. 1965;27: 282–289.](http://paperpile.com/b/Be81W5/bUtI4)

219. [Silver S, Levine E, Spielman PM. Cation fluxes and permeability changes accompanying bacteriophage infection of Escherichia coli. J Virol. 1968;2: 763–771.](http://paperpile.com/b/Be81W5/gU0Ry)

220. [Kutter E, Gachechiladze K, Poglazov A, Marusich E, Shneider M, Aronsson P, et al. Evolution of T4-related phages. Virus Genes. 1995;11: 285–297.](http://paperpile.com/b/Be81W5/6E8W1)
