# Supplementary material for: High-throughput mapping of the phage resistance landscape in E. coli
Source: PLoS Biol. 2020 Oct 13;18(10):e3000877. doi: 10.1371/journal.pbio.3000877 (PMC7553319; doi:10.1371/journal.pbio.3000877)
Supplement: Attachment — Submitted filename: Mutalik_PlosBio_Response to Reviewers.docx [file pbio.3000877.s024.docx]

**Author’s replies to reviewer comments (in Blue):**

REVIEWERS' COMMENTS:

Reviewer #1:

*Overview: The resistance strategies used by E. coli to prevent bacteriophage infection have previously been characterized for several phage:bacteria pairs by using labor-intensive methods that are difficult to scale. To develop an unbiased and comprehensive screen compatible with many bacterial and phage strains, Mutalik and colleagues utilized three previously published E. coli libraries, two for loss of function (RbTn-Seq and CRISPRi) and one for gain of function (DubSeq). With these libraries in two strains of E. coli and 14 diverse phages across three families, the authors were able to validate many existing phage receptors and uncover unexpected pathways involved in phage infection, including colonic acid synthesis and cyclic-di-GMP. Impressively, many hits were validated through plaque assays, and RNA-sequencing was performed to further determine mechanism for previously undescribed hits.*

*All in all, this paper is an incredible amount of work, that I believe will be of interest to a broad readership as it opens up a lot of avenues for future research and adaptation to other phage pairs.*

We would like to thank the reviewer for their kind words and comments on our work.

*Before I can fully recommend for publication, I only have a few concerns to address:*

*Major points:*

**	The mention of resistance mechanisms throughout the introduction primes the reader to think about classic bacteria defense mechanisms such as Restriction/Modification, Methylation, CRISPR etc. Besides CRISPR, which is not expressed in these strains, these are all likely categories of genes that would appear as hits in the DubSeq, overexpression experiments. Why do the authors think that restriction or methylation enzymes are not strong hits?*

In this work, we used the genomic DNA from *E. coli* K-12 BW25113 and BL21 for creating two independent Dub-seq libraries and assayed them in the presence of diverse WT phages. Both *E. coli* strain backgrounds have a disruption in the type I restriction-modification (R-M) system (PMID: [10839821](https://www.ncbi.nlm.nih.gov/pubmed/10839821)), (EcoKI in K-12 and EcoBI in Bl21; encoded by the *hsdRMS* genes have disruptions, and therefore were not screened in our assays), whereas these strains have functional McrA, McrBC, and Mrr restriction systems, encoded by *mcrA*, *mcrBC*, and *mrr* (therefore, these are screened in our assays, but do not show high fitness scores). We believe two key points, gene dosage/expression challenge and competitive ability of strains limit our ability to uncover these host restriction modification (R-M) systems in our Dub-seq assays.

Phage resistance mechanisms such as restriction/modification systems depend on the right balance between the number of phages and expression of phage silencing R-M systems encoded on the host genome (PMID: [22419077](https://www.ncbi.nlm.nih.gov/pubmed/22419077), 32167561, 27319741). Phages encode proteins that interfere with host restriction systems with high affinity (PMID: 20348932, 19691505), and it is possible that higher gene dosage of these restriction systems alone may not be sufficient to fully prevent phage infection (PMID: 32167561, 17135349, 18067538).

Our Dub-seq libraries have randomly sheared host genomic fragments, and we depend on the endogenous native promoters and translation initiation regions for expressing the genes encoded on these fragments. In essence, for strains with Dub-seq fragments encoding R-M systems to show high fitness score, the expression of restriction enzymes has to be at optimum level to combat highly efficient WT phages and also compete with high fitness strains (such as with higher RcsA expression) in the pooled fitness assays. We would like to note here that, *mcrB* (encodes subunit of McrBC system) fragments display high fitness score in the presence of T2 phage on a less competitive solid plate assay (Supplementary Note 1, Supplementary Fig. S3).

We also note that, to reliably uncover R-M systems across different phages, Dub-seq screens can eventually be adopted to assay phage mutants/deletions in genes that encode anti-restriction factors. For example, a similar approach was used earlier (PMID: 17135349) to identify EcoKI (*hsdR*) by screening ASKA library (*E. coli* ORF library) with T7 phage encompassing deletion in gene 0.3 (that silences EcoKI). Similarly, a phage gene-deletion collection can be employed to uncover additional genetic barriers in phage infection by screening a Dub-seq library. For example, a genetic screen using *E. coli* K‐12 and B strains with T7 phage lacking gene 0.7 (T7Δ0.7 ) showed that *udk* (encoding uridine/cytidine kinase) overexpression halted the growth of T7 phage, while the growth of wild‐type T7 phage on these strains is only slightly affected (PMID: 18067538).

To clarify these points we have added a few lines in the summary:

Line #840: “In addition, we also did not identify strong effects for bacterial defense mechanisms (for example, restriction-modification (R-M) and CRISPR systems). In both *E. coli* K-12 BW25113 and BL21, CRISPR and type I R-M systems are disrupted but other R-M systems such as McrA, McrBC, and Mrr are present [[87,89,104]](https://paperpile.com/c/OMS7P0/xQDIP+NvYAK+3MikI). Other than strains with high scoring *mcrB* (encodes subunit of McrBC system, Supplementary note 1) in the presence of T2 phage, we did not see high fitness scores for other restriction systems in our Dub-seq fitness screens. We speculate that the expression of restriction enzyme subunit has to be at optimum level to counter the phage infectivity cycle and also to compete with high fitness strains (such as strains with higher RcsA expression) in the pooled fitness assays. One way to improve the detection of overexpression gene phenotypes is to use phage mutants that lack anti-R-M systems and nonessential regions. For example, T7 phage mutants lacking gene 0.3, gene 1.2 and region 0.3-1.4 regions were used earlier to uncover the overexpression phenotype of *hsdR* (constitutes the restriction subunit of EcoKI), *dgt* (dGTPase) and *udk* (encodes for uridine/cytidine kinase) on T7 phage growth respectively [[50,205]](https://paperpile.com/c/OMS7P0/qyn2p+vmsn).”

We also note the genotypes of strains used in the Methods section:

Line #917: “The genotypes of *E. coli* strains used in the assays include BW25113 (K-12 lacI+rrnBT14 Δ(araB–D)567 Δ(rhaD–B)568 ΔlacZ4787(::rrnB-3) hsdR514 rph-1), *E. coli* MG1655 (K-12 F– λ– ilvG– rfb-50 rph-1) and *E. coli* BL21 (B F– ompT gal dcm lon hsdSB(rB–mB–) [malB+]K-12(λS)). The genetic and phenotypic differences between these strains are well documented [[101,173–175,240]](https://paperpile.com/c/zUN7JX/wCtyM+t4X4M+jvDpv+dHgd7+7s9QZ). We note here that all 3 strains lack an active CRISPR system. Both *E. coli* K-12 BW25113 and BL21 have disruption in the type I restriction-modification (R-M) system (known as EcoKI in *E. coli* K-12 BW25113 and EcoBI in *E. coli* Bl21) encoded by the *hsdRMS* genes, but have functional McrA, McrBC, and Mrr restriction systems, encoded by *mcrA*, *mcrBC*, and *mrr*. *E. coli* MG1655 has functional EcoKI, McrA, McrBC, and Mrr restriction systems.”

*Along these lines, the conclusion section would benefit from a further discussion into the conditions under which a bacterium would actively overexpress the colanic acid synthesis pathway as a phage-defense mechanism.*

To address the reviewer’s comment we have added the following in the conclusion section of the main text:

Line # 820: “We also observed strain specific similarities and differences in the phage infectivity patterns. These results indicate the importance of studying phage resistance across a diverse set of biotic and abiotic conditions, and how they impact the cross-resistance and cross-sensitivity in closely and distantly related organisms. For example, gaining insights into conditions under which a bacterium would actively overexpress the colanic acid synthesis pathway may help us to develop methods to overcome the generalized phage-defense mechanisms”

We also note that we already have the following text in the Results and Discussion section on the colanic acid biosynthesis and Rcs signaling pathways:

Line #590: “Recent studies have highlighted that the activation of the Rcs pathway and capsular biosynthesis are essential to survive in diverse environmental contexts, membrane damage and stress-inducing conditions, including those by antibiotic treatment [[106,166]](https://paperpile.com/c/zUN7JX/azcBT+rNoIs). A number of earlier studies have also highlighted the generation of the mucoid phenotype that probably provides a fitness advantage by blocking phage adsorption [[16,162–165]](https://paperpile.com/c/zUN7JX/0EfVU+vCQco+2zqAG+dea3Y+TfdYM). Our results suggest this might be a generalized mechanism that provides cross-resistance to diverse phages.”

Line #300: “It is known that the Rcs signal transduction pathway functions as an envelope stress response system that monitors cell surface composition and regulates a large number of genes involved in diverse functions including colanic acid synthesis and biofilm formation [[106]](https://paperpile.com/c/zUN7JX/azcBT).”

Line #559: “The Rcs signaling pathway is one of the well-studied signaling pathways in bacteria and is known to regulate a large number of genes involved in diverse functions, including synthesis of colanic acid and biofilm formation in response to perturbations in the cell envelope [[106]](https://paperpile.com/c/OMS7P0/1yS7w)”

**	Most of the phage strains in the study are lytic, as suggested by the choice of using lambda cI857 and p1vir. However, P2 and 186 are known to be lysogenic phages. Whether lytic phages were targeted in this study, and how the lysogenic nature of P2 and 186 could have affected the results is not addressed. Could certain bacterial mutants bias lysogeny of those phages leading to "resistance" as indicated by growth of those lysogens? Also, why is phage P2 excluded from the K-12 DubSeq study?*

We agree with the reviewer that both temperate phages P2 and 186 can convert strains to lysogens, conferring ‘resistance’ against additional P2 and 186 virion predation. The individual strains that underwent lysogen conversion in early rounds of pooled infection would appear fit in the context of P2 or 186 phage growth. Thus, we anticipated enrichment of host factors that increase the rate of lysogeny when either repressed or overexpressed in our RB-TnSeq and Dub-seq assays. However, if lysogeny were to occur at low rates and randomly, we would unlikely see multiple strains or barcodes with conferred fitness (in the same gene) that can compete with highly fit strains that lack either the phage receptor or have enhanced mucoidy phenotype. We (re-)investigated the strain-level fitness scores in these P2 and 186 phage experiments and did not observe any significant high scoring hits that indicate their role in lysogen formation in *E. coli* K-12 and BL21 strains.

We have included this observation on line # 424

“We also did not observe significant fitness scores for host factors that may bias the rate of lysogeny of the two temperate phages (186 and P2) leading to resistance of those lysogens.”

The *E. coli* K-12 Dub-seq data for phage P2 was not included in our analysis because of a low number of reads in these experiments (presumably because none of the overexpressed fragments conferred resistance). We mention this in the methods section, line #1122:

“Phage P2 assays were excluded from the *E. coli* K-12 Dub-seq study as the sequencing runs did not pass the data analysis filters because of low sequencing reads per assay.”

*Minor Points:*

**	Line 156 - "As described later, for comparative purposes, we also performed high-throughput genetic assays in the E. coli BL21 strain background…. Different serotypes of E. coli are also important pathogens with significant global threat and are crucial players in specific human-relevant ecologies [84-86], leading to the question of whether strain variation is also important in predicting the response to phage-mediated selection or whether the mechanisms are likely to be conserved."*

*o	This study does not address pathogenic strains of E. coli and gives an unclear motivation for the use of strain BL21. The organization of this paragraph guides the reader think that non-pathogenic and pathogenic strains will be compared, whereas this study characterizes two similar non-pathogenic strains. These strains may be expected to behave more similarly than a comparison with a pathogenic strain.* ***T****herefore, this paragraph would benefit from a stronger introduction to why strain BL21 is used.*

*Thank you for the suggestion. We have edited and modified the paragraph to highlight the choice of BL21 strain, line #155:*

“Despite *E. coli* being a well-studied model organism [[80,81]](https://paperpile.com/c/OMS7P0/6iY3x+BtLvg), there are significant knowledge gaps regarding gene function [[82]](https://paperpile.com/c/OMS7P0/6cGHo) and phage interaction mechanisms [[26,27,47,83,84]](https://paperpile.com/c/OMS7P0/ucxVE+DVwzW+v05jW+Dm79C+4gCM1). Different serotypes of *E. coli* are also important pathogens with significant global threat and are crucial players in specific human-relevant ecologies [[85–87]](https://paperpile.com/c/OMS7P0/eqTAV+8AaTF+w8593), leading to the question of whether strain variation is also important in predicting the response to phage-mediated selection or whether the mechanisms are likely to be conserved. Such mechanistic information is unavailable for not only pathogenic *E. coli* strains, but also for widely used laboratory non-pathogenic strains such as *E. coli* K-12 and BL21 [[88–90]](https://paperpile.com/c/OMS7P0/NvYAK+shSUx+xQDIP). Historically, both *E. coli* K-12 and B (BL21 ancestor) strains have been used in disparate phage studies and have provided foundational knowledge on phage physiology and growth [[10,91–93]](https://paperpile.com/c/OMS7P0/mNG5I+t6cX6+Emsc0+e3OGT), though phage studies on *E. coli* BL21 are limited relative to those in K-12. Both K-12 and BL21 lack functional CRISPR machinery and type I restriction-modification system that functions as common anti-phage systems, and both strains are unique in their genomic content, physiology, and growth characteristics [[88–90]](https://paperpile.com/c/OMS7P0/NvYAK+shSUx+xQDIP), thereby serve as a valuable reference for comparing closely related host responses to the same phage selection pressures. To demonstrate the efficacy of our approaches to characterize phage resistance mechanisms, and to compare their similarity and differences between two closely related laboratory model strains, we first applied high-throughput genetic screens to *E. coli* K-12 strains (BW25113 and MG1655) and then extended it to *E. coli* BL21 strain (Fig.1)”

We also note that, we have in addition provided more rationale for choosing BL21 strain in BL21 fitness assay section, line #692:

“The genomes of BL21 and K-12 strains have 99% base pair identity over 92% of their genomes, interrupted by deletions or disruptions (by mobile elements) [[88–90]](https://paperpile.com/c/OMS7P0/xQDIP+NvYAK+shSUx). For example, in comparison to K-12, BL21 has a disruption or deletions in the colanic acid pathway, biofilm formation, flagella formation (a 21 gene cluster including flagella-specific *fliA* sigma factor), genes involved in Rcs signaling pathway (*rcsA*, *rcsB*, *rcsC*), the LPS core gene cluster (BL21 forms truncated LPS core with only 2 hexose units compared to normal five hexose units in K-12, Fig. 2B), and is deficient in Lon and OmpT proteases, which are important components of the protein homeostasis network [[88–90]](https://paperpile.com/c/OMS7P0/xQDIP+NvYAK+shSUx). In addition, BL21 also lacks *ompC* and *nfrAB* genes that code for T4 phage and N4 phage receptors in *E. coli* K-12 respectively [[90,178,179]](https://paperpile.com/c/OMS7P0/LZN0Z+m0mHG+xQDIP)”

*o	The use of two novel shiga-toxin phages in this study is very intriguing and can link to the pathogen point made above. The conclusion could benefit from a discussion of how the genes responsible for preventing infection of these two pathogen associated phages differ from non-pathogenic phages of similar structure.*

We agree with the reviewer and have highlighted this in the conclusion section, line #799:

“In addition, extending the screening methods to closely related but different strains would be highly valuable. For example, we used two laboratory *E. coli* strains that have rough LPS architecture where core oligosaccharide is the terminal part of the LPS. However, it is known that the genetic and structural diversity of LPS and the repeat structure O-polysaccharide attached to LPS (to form smooth LPS) is very large in pathogenic and environmental isolates of Enterobacteriaceae, and may impact phage infectivity [[48,58,202–205]](https://paperpile.com/c/OMS7P0/buIcB+b6CK5+XzX20+neM9o+vIqm7+1SjrZ). Although we used non-pathogenic *E. coli* in this work, we did study two phages (CEV1 and CEV2) that infect pathogenic O157:H7 strains, and identified key host factors important for growth of these phages. Our study provides an opportunity to compare these host factor requirements for CEV1 and CEV2 with their structurally similar non-pathogen associated phages T4 and T5 phages respectively. The genetic screens presented in this work and future extension to diverse *E. coli* serogroups may aid in filling the knowledge gap on phage interaction with different antigens and its impact on phage infectivity and resistance”

**	Line 178 - Figure 1.*

*o	The organization of Figure 1 appears like Myoviridae is used with RB-TnSeq, Podoviridae is used with CRISPRi and Siphoviridae is used with GOF. This figure could benefit from either moving the bacterial strains to "Technology" box and then having one arrow from technology to "Phage Strains" or removing the Bacterial Strain information.*

Thank you for the suggestion. We have incorporated this suggestion in our revised figure.

**	Figure 2 - Where are the MOIs described for each phage? The methods section does not describe the MOIs used.*

We have improved Figure 2 with a defined MOI colored bar. We have included a new Supplementary Table S13 with MOIs for each assay. We also have MOIs defined in the Supplementary tables S1, S4 and S5.

**	Figure 3 - In the CRISPRi screen, igaA is such a strong hit for all phages besides 186. In RbTn-Seq, however, igaA is a strong hit for 186. Why do the authors think that this result was not repeatable with CRISPRi? Does phage 186 contain a depolymerase to degrade the capsule?*

As *igaA* scores high in other CRISPRi assays (for other phages), the gRNA is functional in downregulating *igaA*. However, we do not currently understand the variability in fitness scores; we propose that it could relate to competition with other strains.

**	Line 495 - "This is the first systematic analysis of how gene overexpression impacts phage resistance"*

*o	This is overstated given that Qimron 2006 use the entire ASKA collection to probe T7 resistance.*

We agree with the reviewer and have edited the text accordingly, Line #505.

“Following earlier work on phage T7 [[51]](https://paperpile.com/c/OMS7P0/qyn2p), this is the first global survey of how host gene overexpression/dosage impacts resistance to diverse phages belonging to 3 families.”

**	Lines 1010 and 1035 - Should this should be -80°C?*

Thanks. We have corrected the error.

**	Supplementary Figure S5 - There are no 0.1mM IPTG pictures for T5 and T6 phages*

We do have these pictures (just above 0.1 mM IPTG panel). We used No-IPTG or 0.1 mM IPTG for inducing expression of genes from ASKA plasmid depending upon the toxicity observed.

*Reviewers #2:*

*[identify themselves as Sankar Adhya and Shayla Hesse]*

*Mutalik et al apply the array of specific and tractable genome-wide perturbations present in RB-TnSeq, Dub-Seq, and CRISPRi E. coli libraries to broadly probe the phage resistance landscape for multiple phage-bacteria pairs. Their work highlights the key advantages of such an approach: overcoming the limitations of complete LOF screens and venturing into the largely uncharted territory of GOF and intergenic screens - all while maintaining a relatively high degree of technical efficiency. Their work reveals novel insights on an age-old subject (coliphage-host interaction and evolution) and is well-presented in both written word and graphics. The degree to which non-phage-receptor- related resistance genes varied for the 14 different phages was particularly interesting, as well as the central role of Rcs signalling (igaA downregulation and rcsA upregulation) in modulating bacterial sensitivity to phage infection more generally.*

We would like to thank the reviewers for their kind words and comments on our work.

*I recommend publication of the manuscript after authors satisfactorily respond to some comments listed below.*

*Comments:*

A RB-TnSeq E.coli library seems fairly straightforward, but I'm sure that CRISPRi and Dub-Seq library generation are trickier propositions. One cannot easily assess the overall quality of sgRNAs (In terms of target affinity and avoiding off-target effects). Can you control gene dosage in Dub-Seq library generation? If not, what is the range of variability for different plasmids?

We have an accompanying paper on the overall quality of sgRNAs used in the CRISPRi assays (doi: <https://doi.org/10.1101/2020.03.04.975888>). The issue of off-target effects has been addressed in numerous CRISPR-Cas papers (for example, PMID: 30166482, PMID: [29765036](https://www.ncbi.nlm.nih.gov/pubmed/29765036)). Typically, two approaches are used (to deal with off-target effects of CRISPRi): (1) have multiple guides per target and discard outliers or make some composite score that can be trusted (2) have a validation data set with known essential targets for a condition and negative control guides that shouldn't have an effect as that will give an idea of true positive, and false positive rates. We have used both of these approaches to filter out any off-target effects in our CRISPRi assays.

We have included this line in the method section, Line 1127: “The design and construction of *E. coli* MG1655 CRISPRi library and the overall quality of sgRNAs used are detailed elsewhere [[69]](https://paperpile.com/c/OMS7P0/56gHq)”

There are ways to control the dosage (both in CRISPRi and Dub-seq plasmid based tools) - one can either use different plasmid systems or put the library on conditional origins of replication where one can titrate the abundance of the plasmids (e.g. PMID: 21787416). The copy number of plasmids used in this work for Dub-seq is ~15-20 and CRISPRi is ~100 (PMID: 21933410). However, we acknowledge that the plasmid copy number varies depending upon the cloned region on the plasmid, associated stress effects on cellular physiology and growth conditions (e.g. PMID: 21647376).

We have included the copy number of Dub-seq plasmids in the results section, Line #437: “This library is made up of randomly sheared 3 kb genomic DNA of *E. coli* K-12 BW25113 cloned into a dual barcoded vector with the copy number of ~15-20 “

*Specific Comments*

*Strain-specific differences between K-12 and BL21: Side-by-side comparison of Fig. 2a and 7 reveal some notable differences in RB-TnSeq results, while there were many more that were observed with the Dub-Seq data - do you think most of them are artifacts? What do you think are the most likely explanations?*

BL21 Dub-seq data is based on a different Dub-seq vector than that used in our previously constructed BW25113 library (PMID: [30659179](https://www.ncbi.nlm.nih.gov/pubmed/30659179)). We acknowledge that BL21 data is noisier than the BW25113 Dub-seq library and observed a higher number of high scoring single fragments in BL21 library. Therefore we applied stringent data filters for identifying reliable positive hits. Nevertheless, the data in Figure 7(B) Dub-seq data does show a higher number of hits than compared to Figure 4(A). Some of these high scoring genes for phage Lambda assay in Figure 7 (B) for BL21 Dub-seq library seem genuine based on known information (detailed in Supplementary Note 9). Nevertheless, there are some y-genes for which we do not have sufficient information to label them as false positives.

*Dub-Seq: What is the average increase in gene copy number on a per cell basis?*

Our Dub-seq libraries are constructed with a pBBR1 vector backbone with ~15-20 copies per cell. Considering that the libraries are an ensemble of different constructs and different size genomic regions cloned, we do not know the gene copy number variation on a per cell basis.

*Figure 5: consider moving to Supplemental, not sure how much visualization of those raw data enhances the reader's understanding of the key points of the paper*

Thank you for the suggestion. We would like to retain Figure 5 in the main text to illustrate that some of the top scoring candidates we uncover in our genetic screens are genuinely positive and show strong phage resistance phenotype.

*Page 22, Lines 850-855: The authors dutifully point out the potential for phage-resistant strains with significantly lower fitness to be outcompeted to the point of being obscured. It may also be worth pointing out that the relative fitness of these lab-generated strains is distinct from the likelihood that their cognate mutant will naturally evolve under phage selection pressure. (A more obvious limitation, yes, but also more consequential. And particularly relevant given the authors' claim in the abstract that an upshot to the application of their methods may be the generation of "datasets that allow predictive models of how phage-mediated selection will shape bacterial phenotype and evolution.")*

We agree with the reviewer. It is possible that the relative fitness of these lab-generated strains is distinct from the naturally evolved mutants under phage selection pressure. That being said, a number of reviews and published data on naturally evolved phage resistance genotypes have indicated that the most frequently observed resistance determining factor is the receptor deletion (for example, Lenski RE, Adv. Microbial Eco., 1988, pp 1-44; and others such as PMID: 30281587; PMID: 31992617; PMID: [22927964](https://www.ncbi.nlm.nih.gov/pubmed/22927964); PMID: 13680205). Studies are ongoing in our laboratories to compare genotypes of LOF and GOF screen candidates reported in this work with naturally evolved bacterial strains under phage selection.

To clarify this point, we have included this sentence in the results section, line #838:

“We note here that the relative fitness of these lab-generated strains may be distinct from the likelihood that their cognate mutant will naturally evolve under phage selection pressure.”

*Page 30, Line 183-184:* ***The igaA mutant map weblink returned an error message.***

Thank you for pointing this out. We have corrected the weblink to the igaA mutant map. We have edited this in the revised manuscript.

https://benchling.com/s/seq-pZEspfGx8K9tYzixzMoJ

*Supplementary table S12: recommend addition of phage genome accession numbers/links for the non-canonical phages*

We have included genome accession numbers in the Supplementary Table S12. The genome sequences of novel phages (LZ4, CEV1, CEV2) used in this work will be reported elsewhere (In prep, Kutter lab, Evergreen State College).

Reviewer #3:

[identifies himself as Jeremy J Barr]

*The manuscript by Mutalik et. al., presents a tour de force in mapping the phage resistance landscape across two E. coli strains. Using a combination of LOF (Tn-Seq & CRISPRi) and GOF (DubSeq) techniques, they screened for host factors associated with phage resistance across 14 E. coli phages, many of which are well established model phages, along with novel and less-well characterised phages, demonstrating the broad applicability of the technique. Using these techniques, they confirm established phage-receptor mutants, while also uncovering novel resistance mechanisms, some of which were validated using conventional knock-out and complementation.*

*The amount of work invested into this manuscript is both impressive and staggering, and this could have easily been split across multiple manuscripts. Yet the amount of data and analysis presented did make parts of the manuscript very detailed and difficult to read, with parts that could be reduced to improve readability. Overall, I have no major concerns with the experimental approach and analysis conducted and believe this is a very important paper to the field. Most of my comments address readability and accessibility of the manuscript.*

We would like to thank the reviewer for his kind words and comments on our work.

*I had two major comments with the paper that I felt should be addressed.*

*1)	MOIs and both host and phage concentrations for these most experiments are not reported in text or methods. The authors simply state 'varying MOIs' and provide a qualitative scale for this (with exception of CRISPRi where MOI 1 was used). It is important that both the MOIs and concentrations used in each assay are report to confirm their validity. Please report all MOIs along with actual concentrations of phage and host used.*

We agree with the reviewer. We have updated the manuscript and now provide host and phage concentrations and MOI numbers in Supplementary Table S13 for each phage/per experiment. In addition, we have included a colored bar for MOIs in Fig. 2 and Fig. 4. These MOI numbers were provided in the Supplementary Tables S1, S4, S5, S8 and S9 in our earlier version, but not very obvious to the reader. By including the MOI colored bar in figures and including a separate Supplementary Table S13 detailing every MOI data should be helpful. Because the assays reported in the manuscript used different batches of phage preparation, these MOIs are different between RB-TnSeq assays, Dub-seq assays and CRISPRi assays.

*2) I felt that the authors ignored the limitations of their approach, particularly the time and costs associated with establishing these libraries coupled with the limited phage that may target these library strains. Suggestion is to add an extra section to the discussion on this, and speculating how these limitations may be overcome in future developments of the technology.*

Thanks for this comment. We have included additional text that highlights the limitations of these technologies and new methods that can address some of these limitations, Line #828:

*“*The strong positive fitness scores we observe due to the selection pressure during these pooled fitness experiments is both a strength and limitation of our methods. The strength of these screens is that we can rapidly identify host factors crucial in phage infectivity cycle because they display a very strong fitness score when disrupted (for example, a phage receptor and its regulators in LOF screen). This strong positive selection, however, limits the detection of intermediate phage resistance routes (for example, effect of *trxA and cmk* deletion on T7 phage growth) whose disruption may lead to strains with relatively poor or neutral fitness and will be swept from the population. We addressed this limitation by using solid agar plate assays, where direct competition among resistant types may be reduced. Future fitness assays can be extended to droplet microfluidics platforms [[207]](https://paperpile.com/c/OMS7P0/Swop) to uncover intermediate fitness phenotypes and retain the high-throughput scalability of these approaches. We note here that the relative fitness of these lab-generated strains may be distinct from the likelihood that their cognate mutant will naturally evolve under phage selection pressure [[208]](https://paperpile.com/c/OMS7P0/ZVzWC). In addition, we also did not identify strong effects for bacterial defense mechanisms (for example, restriction-modification (R-M) and CRISPR systems). In both *E. coli* K-12 BW25113 and BL21, CRISPR and type I R-M systems are disrupted but other R-M systems such as McrA, McrBC, and Mrr are intact [[88,90,105]](https://paperpile.com/c/OMS7P0/NvYAK+3MikI+xQDIP). Other than strains with high scoring *mcrB* (encodes subunit of McrBC system, Supplementary note 1, Supplementary Fig. S3) in the presence of T2 phage, we did not see high fitness scores for other restriction systems in our Dub-seq fitness screens. We speculate that the expression of restriction enzyme subunit has to be at optimum level to counter the phage infectivity cycle and also to compete with high fitness strains (such as strains with higher RcsA expression) in the pooled fitness assays. One way to improve the detection of overexpression gene phenotypes is to use phage mutants that lack anti-R-M systems and nonessential regions. For example, T7 phage mutants lacking gene 0.3, gene 1.2 and region 0.3-1.4 regions were used earlier to uncover the overexpression phenotype of *hsdR* (constitutes the restriction subunit of EcoKI), *dgt* (dGTPase) and *udk* (encodes for uridine/cytidine kinase) on T7 phage growth respectively [[51,209]](https://paperpile.com/c/OMS7P0/vmsn+qyn2p).

The screening technologies presented in this work are scalable to study phage resistance mechanisms in diverse organisms. Two key considerations in this regard that need to be accounted for are (1) the availability and extendability of genetic tools to the organisms of interest, and (2) the cost and time in creating the RB-Tnseq, CRISPRi and Dub-seq libraries. Based on our experience, the cost of creation of these libraries falls in the range of $4000 to $8000 per library and takes about 3 months on an average for building fully characterized libraries. The newly developed sequencing technologies and extendibility of genetic tools to non-model organisms can further reduce the time and cost of generating the LOF and GOF libraries [[70,210,211]](https://paperpile.com/c/OMS7P0/Hr9u+Qn0N+xFq8). Once the libraries are built, the running cost of these genome-wide assays across 96 conditions cost about $10 per assay and serve as a standardized reagent tool for in-house experiments or for sharing and comparing data across different laboratories. The ease and economics of these genetic screens enables extendibility of phage resistance assays in diverse conditions that simulate the natural environment and may provide valuable insights on host fitness and host-phage co-evolutionary dynamics under more ecologically relevant conditions.”

*3)*	***At points the manuscript was overly descriptive and long.*** *The authors should consider reducing the amount of text in sections to improve readability and focus of each section, some specific examples are below.*

We have made an effort to improve the readability of the work in the revised submission, including removing some repetitive text (see below).

*Lines 154-159 & 164-166 - Sections describing the libraries and hosts were repetitive, please combine points/sections*

Based on the reviewer’s recommendation, we have removed repetitive text and combined the points in this section. We also addressed a couple of comments made by other reviewers for this section, especially highlighting the motivation for choosing BL21 strain in our work. The new text reads, line #155:

“Despite *E. coli* being a well-studied model organism [[80,81]](https://paperpile.com/c/OMS7P0/6iY3x+BtLvg), there are significant knowledge gaps regarding gene function [[82]](https://paperpile.com/c/OMS7P0/6cGHo) and phage interaction mechanisms [[26,27,47,83,84]](https://paperpile.com/c/OMS7P0/ucxVE+DVwzW+v05jW+Dm79C+4gCM1). Different serotypes of *E. coli* are also important pathogens with significant global threat and are crucial players in specific human-relevant ecologies [[85–87]](https://paperpile.com/c/OMS7P0/eqTAV+8AaTF+w8593), leading to the question of whether strain variation is also important in predicting the response to phage-mediated selection or whether the mechanisms are likely to be conserved. Such mechanistic information is unavailable for not only pathogenic *E. coli* strains, but also for widely used laboratory non-pathogenic strains such as *E. coli* K-12 and BL21 [[88–90]](https://paperpile.com/c/OMS7P0/NvYAK+shSUx+xQDIP). Historically, both *E. coli* K-12 and B (BL21 ancestor) strains have been used in disparate phage studies and have provided foundational knowledge on phage physiology and growth [[10,91–93]](https://paperpile.com/c/OMS7P0/mNG5I+t6cX6+Emsc0+e3OGT), though phage studies on *E. coli* BL21 are limited relative to those in K-12. Both K-12 and BL21 lack functional CRISPR machinery and type I restriction-modification system that functions as common anti-phage systems, and both strains are unique in their genomic content, physiology, and growth characteristics [[88–90]](https://paperpile.com/c/OMS7P0/NvYAK+shSUx+xQDIP), thereby serve as a valuable reference for comparing closely related host responses to the same phage selection pressures. To demonstrate the efficacy of our approaches to characterize phage resistance mechanisms, and to compare their similarity and differences between two closely related laboratory model strains, we first applied high-throughput genetic screens to *E. coli* K-12 strains (BW25113 and MG1655) and then extended it to *E. coli* BL21 strain (Fig.1)”

*Lines 171-172 - Please highlight in text the novel and STEC targeting phages*

We revised the text to read, line #190: “These phages include 11 canonical and well-studied coliphages each having overlapping but distinct mechanisms of host recognition, entry, replication and host lysis [[90]](https://paperpile.com/c/OMS7P0/onjZ9), and two recently reported phages (CEV1 and CEV2), which are known to kill pathogenic shiga-toxin producing *E. coli* (STEC, O157:H7), and one novel coliphage (LZ4).”

*Line 188 - term 'are' please change to past tense*

We revised to:: *“*The barcoded LOF or GOF libraries were grown competitively in a single-pot assay and challenged with phages.”

*Lines 194-200 - the descriptions of positive fitness scores were confusingly written and long, I understand the rationale, but recommend simplifying this section*

We have revised the text to read, Line #196: “The barcoded LOF or GOF libraries were grown competitively in a single-pot assay and placed under phage-mediated selection. Any variation in fitness resulting from disruption or over-expression of specific genes in the presence of phage is therefore exposed to selection, allowing for evolutionary change within the population. Wwe monitor these changes by quantifying the abundance of the DNA barcode associated with each strain. The individual strain abundances are then converted to gene fitness scores, which we define as the normalized log2 change in the abundance of mutants in that gene (Methods). A positive gene fitness score (“positive hit”) indicates the strain with deletion or overexpression of a gene(s) realized an increase in relative fitness in the presence of a particular phage (i.e. strains with these genetic changes are more resistant to the phage and are enriched in our phage selection assay). A positive fitness score for a gene in our LOF assay indicates that its encoded product (for example, a phage receptor) is needed for successful phage infection, while a positive fitness score for a gene in our GOF assay indicates that its encoded product (for example, a repressor of phage receptor) prevents the phage infection cycle. Negative fitness values, which suggest reduced relative fitness, indicate that the gene(s) disruption or overexpression result in these strains being more sensitive to the phage than the typical strain in the library.”

*Lines 228-229 - See comment above, please report both your MOIs and phage/host concentrations used.*

As mentioned above, we have now included MOI numbers in the Supplementary Table S13 for each phage/per experiment. We have also included a colored bar for MOI in the Fig. 2 and Fig. 4 heatmaps.

*Line 236 - should read 'more than one'*

We made this change.

*Lines 243-244 - This section reads like either the LPS or proteinaceous receptors are required for phage infection, this may be the case for your 14 phages studied, but not as a general rule - please rewrite to highlight the large diversity of potential phage receptors*

We revised the text to, Line #259: “Phage infection initiates through an interaction of the phage with any cell-surface exposed molecules (‘receptors’), which could be lipopolysaccharide (LPS) moieties, teichoic acids, capsules and proteinaceous components.”

*Figure 2 - Recommend adding darker border lines between phages as it is difficult to interpret inner parts of the graph. Also recommend adding something to the figure panel C to highlight that phages affected are colored purple.*

Thanks. We have included this suggestion in our revised submission. We have not added darker border lines between phages in the heatmap, as it became too dense to read. We have instead made an effort to improve the readability of heatmap data by including phage names on the top axis.

*Lines 363 - briefly re-iterate what a high fitness score implies in this assay*

To address this reviewer comment,. we included this text, Line #347, “During these pooled CRISPRi assays, strains that carry an sgRNA targeting a feature on the *E. coli* genome important for phage infection (for example, a phage receptor) will increase in abundance and will have a positive fitness score.”

*Lines 446 - Is not clear where this genomic DNA comes from, assuming this is taken from the same K-12 host, but this should be briefly explained here.*

To make this clearer, we revised to, Line #437: “This library is made up of randomly sheared 3 kb genomic DNA of *E. coli* K-12 BW25113 cloned into a dual barcoded vector”

*Lines 453 - "67 genome wide GOF competition" - Can you clarify what this means? Does one genome-wide GOF assay encompass a single library containing the whole K12 genome of sheared 3kb DNA?*

Yes, we have clarified it as below, Line #445:

“In total, we performed 67 genome-wide GOF fitness assays in the presence of 13 different phages at varying MOIs, both in planktonic and solid plate assay format and 4 no-phage control experiments (Methods). Here, one genome-wide GOF fitness assay encompasses an ensemble of barcoded vectors with sheared 3 kb genomic region of the whole *E. coli* K-12 BW25113.”

*Lines 457-458 - Again re-iterate what positive-growth implies in this experiment - multiple other points in manuscript this could be explained (lines 517,*

To clarify this, we have included this line #454 “A positive fitness score for a gene in our Dub-seq assay indicates that the overexpression of that gene interferes with successful phage infection.”

And line #474 “Overexpression or higher dosage of these genes interferes with the phage infectivity cycle and impart fitness benefits to the host.”

*Lines 485-488 - Further explanation needed here, does the increased expression of PDEs lead to degradation of cyclic-di-GMP and thereby inhibition of biofilm formation? How does this confer broad resistance to phages?*

We have included this sentence to clarify this point, line #497: “The fitness benefit of increased dosage of PDEs is presumably mediated by their degradation of cyclic-di-GMP. We infer that high levels of cyclic-di-GMP are required for phage N4 infection, although the mechanism is unclear.”

*Lines 550-561 - reduce complexity here*

We have edited this section, and new text reads, Line 559:

“The Rcs signaling pathway is one of the well studied signaling pathways in bacteria and is known to regulate a large number of genes involved in diverse functions including synthesis of colanic acid and biofilm formation in response to perturbations in the cell envelope [[105]](https://paperpile.com/c/OMS7P0/1yS7w). Downregulation of *igaA, a* negative regulator of the Rcs signaling pathway, causes resistance to infection by T4, λ and 186 phages [[51]. Overexpression of RcsA, the regulator at the end of the pathway, confers](https://paperpile.com/c/OMS7P0/5lcA8) resistance to T7 phage [[50]](https://paperpile.com/c/OMS7P0/qyn2p). We found that activation of the Rcs pathway (by either disruption in *igaA* (*yrfF*) or overexpression of *rcsA*) confers resistance to all of the phage we studied. We also observed a high fitness score for sgRNA targeting *dsrB* (a gene of unknown function located downstream of *rcsA*) in CRISPRi screens in the presence of N4 phage (Fig. 3A). In agreement with our Dub-seq screen data, we observed the EOP of T2, T3, T4, T5, T6, 186, λ, CEV1, CEV2, LZ4, and N4 phages is compromised when *rcsA* is overexpressed (Fig. 5B). Similarly, a *dsrB* deletion strain showed severe N4 phage plating defects, confirming the high fitness scores in our CRISPRi screen (Fig. 5A).”

*Lines 566-567 ¬- regarding Rcs pathway, useful to also state what this pathway activates (e.g., colonic acid)*

We changed the text to read, Line #574: “To validate that this domain is indeed dispensable for strain viability and also important in phage resistance, we successfully reconstructed the igaA insertion mutant (Methods). This mutant strain displayed a mucoidy phenotype indicative of increased activation of colanic acid biosynthesis via the Rcs signaling pathway [[105,162]](https://paperpile.com/c/OMS7P0/1yS7w+eLUgf).”

*Lines 601-637 - In general this section was difficult to read and a lot of detail could be cut to focus on the main outcomes*

Based on the reviewer’s comment we have shortened and edited this section to improve the message. The new text reads, Line #630:

*“*Cyclic-di-GMP is a key bacterial secondary signaling molecule involved in regulation of diverse cellular functions. The top 8 hits in our LOF and GOF screens for N4 phage resistance included enzymes that catalyze synthesis and degradation of cyclic-di-GMP. Diguanylate cyclase-J (encoded by *dgcJ*) is involved in the biosynthesis of cyclic-di-GMP and is one of the top scorers in both RB-TnSeq and CRISPRi LOF screens, whereas the seven c-di-GMP-specific phosphodiesterases (PDEs) that are involved in degradation of c-di-GMP showed the highest fitness scores in the Dub-seq screen. Though the signaling network of cyclic-di-GMP is complex, deletion of diguanylate cyclases (DGCs) or overexpression of PDEs is known to reduce cyclic-di-GMP levels, inhibit biofilm formation, reduce biosynthesis of curli, and increase motility [[148–150,170]](https://paperpile.com/c/OMS7P0/TGfDI+2fTlW+DUAV2+UbqRH). The high fitness scores for *dgcJ* in RB-TnSeq and CRISPRi screens is intriguing considering it is one of the 12 DGCs encoded on *E. coli* K-12 genome [[171,172]](https://paperpile.com/c/OMS7P0/SDqHf+4Go7o), and none of the other DGCs show phenotypes in our screens. Similarly, *E. coli* K-12 genome codes for 13 PDEs in total [[171,172]](https://paperpile.com/c/OMS7P0/SDqHf+4Go7o), and we find 6 of these PDEs show a phage resistance phenotype when overexpressed. Our EOP estimations with N4 phage showed severe plating defect (EOP <8E-8) on *pdeO (dosP)*, *pdeR (gmr)*, *pdeN (rtn)*, *pdeL* (*yahA)*, *pdeB (ylaB)*, *pdeI (yliE)* overexpressing strains, while minor plating defect on *dgcJ* mutant (EOP of 0.8) (Figs. 5A, 5B). The plating defect of N4 phage on *dgcJ::kan* could be reverted back when the *dgcJ* was provided *in trans* (Supplementary Fig. S5).

To gain insight into how the overexpression of specific genes might affect phage infection pathways, we performed differential RNA-seq experiments (standard growth conditions, in the absence of phage) on five c-di-GMP phosphodiesterases (*pdeL*, *pdeB*, *pdeC*, *pdeN* and *pdeO*) overexpressing strains, each of which shows resistance to N4 phage (Methods). RNA-seq experiments on the *pdeL* overexpression strain revealed large changes in the *E. coli* transcriptome relative to the wild-type BW25113 strain, with 103 genes significantly upregulated (log2FC > 2, q < 0.001) and 109 genes significantly downregulated (log2FC < -2, q < 0.001) (Fig. 6E, 6F, Supplementary Table S6 and S7). N4 phage receptor genes *nfrA* and *nfrB* were not differentially expressed. Similar to *pdeL* overexpression, overexpression of the other four PDEs also did not show substantially different expression of *nfrA* and *nfrB*, suggesting that N4 resistance phenotype in these instances does not appear to be via transcriptional regulation of the N4 phage receptor genes (Supplementary Table S6 and S7). Further studies are underway to gain more insights on how c-di-GMP levels influence N4 phage infection cycle.“

*Section starting 638 - This section seems entirely focused on N4 phage infectivity - which is needed, but the heading doesnt convey this. please reword*

We agree with the reviewer. The revised heading reads, Line #662:

“Validation of other top scoring genes displaying N4 phage resistance phenotype”

*Figure 6 - Panel B: could briefly state the mechanism of igaA knock down and how it leads to colanic acid and EPS activation. Panel D: Similar comment, briefly explain mechanism of up and down regulation*

Thank you. We have edited the figure to include more details on regulation. The edited caption reads, Line #600:

“(B) Schematic of the Rcs phosphorelay pathway. Mutations in *igaA* (shown as stars) identified in this work activate Rcs signaling pathway and induce *rcsA* expression, colonic acid and EPS biosynthesis pathway. Disruption in *igaA* or overexpression of *rcsA* show a mucoidy phenotype and broad resistance to different phages.

(D) Schematic of *ompF* and *ompC* expression regulation via EnvZ-OmpR and YgbE. Overexpression of *ygbE* downregulates *ompC* expression (via unknown mechanism) and upregulates genes involved in lipid-A modification, probably the reason for resistance to phage T4. “

*Lines 775-776 - include brief description on which host library was made from - assuming BL21*

We revised the text to, Line #743:: “Finally, to investigate whether increased gene copy number of host factors interferes with phage growth in BL21, we constructed a BL21 Dub-seq library. This library is made up of randomly sheared BL21 genomic DNA cloned into a dual barcoded vector and is made up of a total 65,788 unique 3 kb fragments (Methods, Supplementary Fig. S8).”

*Figure 7 - Why not highlight yellow boxes for confirmed phage receptors again?*

We have edited the Figure to show agreements with K-12 dataset.

Finally, While this manuscript was under review, a new paper from the Turner lab came out in *PNAS* using INseq approach for screening phage receptors (PMID: 32675236). We have included this following note in our revised results section, Line #323.

“While this manuscript was under review, a new work using transposon sequencing to screen receptors for T2, T4, T6 and T7 phages came out [[119]](https://paperpile.com/c/OMS7P0/7oDQ). Our results are largely in agreement with this new study, with few differences that point to difference in the mutant library size (17,100 insertions in 3,253 bacterial genes compared to our RB-TnSeq library with 152,018 barcoded insertions in 3,728 genes).”
